# Age differences in immunity to human seasonal coronaviruses and the immunogenicity of ChAdOx1 nCoV-19 (AZD1222)

**DOI:** 10.1016/j.ebiom.2025.105847

**Published:** 2025-07-16

**Authors:** Sandra Belij-Rammerstorfer, Emma Sheehan, Grace Li, Sagida Bibi, Daniel Wright, Merryn Voysey, Cameron Bissett, Ninisha Barman, Susana Camara, Audrey Au Yong, Sue Ann Costa Clemens, Mae Harris, Amy Flaxman, Jordan Barrett, Khiyam Hussain, Gareth Lipunga, Robert H. Shaw, Holly Smith, Stanley Cheruiyot, John N. Gitonga, Daisy Mugo, Henry K. Karanja, George M. Warimwe, Mainga M. Hamaluba, Lily Y. Weckx, Andrew J. Pollard, Teresa Lambe

**Affiliations:** a Oxford Vaccine Group, Department of Paediatrics, University of Oxford, UK; b Institute for Global Health, Centro Servizi di Ateneo Santa Chiara Lab, University of Siena, Siena, 53100, Italy; c Jenner Institute, University of Oxford, Oxford, UK; d Department of Biochemistry, University of Oxford, Oxford, UK; e KEMRI-Wellcome Trust Research Programme, Kilifi, Kenya; f Centre for Tropical Medicine and Global Health, University of Oxford, UK; g Department of Paediatrics, Universidade Federal de São Paulo, São Paulo, Brazil; h Oxford NIHR Biomedical Research Centre, Oxford, UK; i Chinese Academy of Medical Sciences Oxford Institute, Oxford, UK

**Keywords:** SARS-CoV-2, ChAdOx1 nCoV-19, Age groups, Human seasonal coronaviruses, FcγR

## Abstract

**Background:**

ChAdOx1 nCoV-19 (AZD1222) vaccine was widely deployed to protect against severe COVID-19 in adults, but the relationship between pre-existing immunity to human seasonal coronaviruses (HCoVs) and vaccine-induced SARS-CoV-2 (SCoV2) response across age groups remains unclear.

**Methods:**

We analysed SCoV2 and HCoVs antibody profiles in UK volunteers (aged 6–≥70), assessing antibody levels, avidity, and FcγR binding after receiving one or two doses of ChAdOx1 nCoV-19. Adult cohorts from trials in Brazil and Kenya were also included to evaluate geographical impacts on baseline HCoVs and SCoV2 induced response.

**Findings:**

In the UK cohort, younger individuals had higher SCoV2 IgG, avidity, FcγR binding and cross-reactivity, particularly towards OC43 and HKU1. The greatest differences were seen after the first dose of ChAdOx1 nCoV-19, but these effects diminished after the second dose. Although baseline HCoVs IgG varied geographically, similar trends were observed across adult cohorts with younger adults showing higher SCoV2 IgG compared to older adults (UK and Brazil).

**Interpretation:**

These findings contribute to a better understanding of the immunogenicity of ChAdOx1-based vaccines in various age groups. Determining whether this applies across other vaccines using same platform is essential for evaluating the viability of one-dose regimens in outbreak responses.

**Funding:**

The clinical trials COV002, COV003, COV004, and COV006 were made possible by funding from 10.13039/100004325Astra Zeneca, the NIHR and the University of Oxford, 10.13039/501100000276UK Department of Health and Social Care, through the 10.13039/501100000272UK National Institute for Health and Care Research, the 10.13039/100010269Wellcome Trust (220991), and 10.13039/501100006041Innovate UK (project 971614).

**Research in context**

**Evidence before this study**

Previous publications have extensively described the relationship between pre-existing immunity to human coronaviruses (HCoVs) and immune responses to SARS-CoV-2 (SCoV2) infection. Several studies reported on mRNA vaccines and viral-vectored vaccines like ChAdOx1 nCoV-19 to induce neutralising and binding antibodies against SCoV2 across different age groups. However, limited research has focused on the role of pre-existing HCoVs immunity in shaping SCoV2 vaccine-induced responses across different age groups. While the antibody response profile to SCoV2 vaccination has been described across different age groups after mRNA vaccine (BNT162b2), a multifunctional antibody response to ChAdOx1 nCoV-19 has previously been demonstrated in adults only.

**Added value of this study**

This study is the first to systematically evaluate the relationship of pre-existing HCoVs immunity and quantitative antibody response to ChAdOx1 nCoV-19 immunisation across different age groups and geographical regions. This study also provides a comparison of the in-depth SCoV2 specific functional antibody profile between age groups in terms of antibody avidity, subclass and isotype, and FcγR binding. This has not been previously described for the ChAdOx1 nCoV-19 vaccine.

**Implications of all the available evidence**

The results from this study show age-related differences in the immune response to ChAdOx1 nCoV-19. Younger individuals have highly functional and a more cross-reactive antibody pool after a single dose, while older individuals require additional doses to enhance their functional antibody responses. These differences may originate from different exposure histories to HCoVs. The study has important implications for vaccine development and deployment strategies, particularly for viral-vectored platforms such as ChAdOx1 in pandemic and outbreak settings.

## Introduction

The ChAdOx1 nCoV-19 (AZD1222) adenoviral-vectored vaccine was widely deployed to protect adults against severe COVID-19 during the pandemic in both homologous and heterologous regimens.^1^, ^2^, ^3^ In addition, a phase II trial undertaken in children and adolescents demonstrated immunogenicity with a good safety profile.^4^ As worldwide vaccination against SARS-CoV-2 (SCoV2) continues, delineating functional characteristics of the humoural response is of high importance especially amongst older age groups. Understanding age-related differences in vaccine-induced immune response is critical to ensure the use of best vaccine platforms for both routine and outbreak immunisation schedules.

Neutralising antibodies have been shown to be a correlate of protection against SCoV2.^5^ However, a considerable body of data also suggest key roles of other non-neutralising, Fc-dependent antibody effector functions^6^, ^7^, ^8^ and it has been shown that these can vary between age groups.^9^ Whilst the antibody response profile to SCoV2 vaccination has been described across different age groups after mRNA vaccine (BNT162b2),^10^ a multifunctional antibody response to ChAdOx1 nCoV-19 has previously been demonstrated in adults only.^11^

Sero-epidemiology studies indicate that seroprevalence to seasonal coronaviruses (HCoVs), betacoronaviruses OC43 and HKU1 and alphacoronaviruses 229E and NL63, increases rapidly in early childhood and is maintained at high level in adulthood.^12^^,^^13^ Throughout life, infections with HCoVs are frequent, with young children generally showing higher HCoVs prevalence rates compared to adults.^14^, ^15^, ^16^ The resulting non-sterilising immunity is transient and cross-reactive with other HCoVs.^17^

Older adults, who are likely to have encountered multiple strains of HCoVs, may exhibit altered responses to SCoV2 due to prior immune exposures.^18^^,^^19^ There is an ongoing discussion as to whether pre-existing antibody responses against HCoVs as well as pre-existing memory T cells have a beneficial role in reducing SCoV2 disease severity.^20^, ^21^, ^22^, ^23^ However, understanding the role of pre-existing HCoVs immunity and its cross-reactivity with vaccine-induced antibodies is less known and could reveal how age modulates immune responses to vaccines. Whilst some published data supports existence of immune imprinting in the context of SCoV2 vaccination,^24^ there has been no formal comparison of how variation in seasonal coronavirus immunity may be associated with differences in response to COVID-19 vaccination between adults and children. We therefore assessed if ChAdOx1 nCoV-19 vaccination induced a different quantitative and qualitative antibody response in different age groups in the context of their previous exposure to HCoVs.

Other factors can influence viral infection rates resulting in variability in immune status such as genetics and differing geographical regions.^25^, ^26^, ^27^ HCoVs prevalence rates are known to vary between regions, with lowest rates reported in Southeast & East Asia (2–3%), compared to Africa (6–14%) and Europe and South America (6–7%).^14^ Climatic and socio-economic factors further contribute to this variability.^14^^,^^15^ We therefore examined whether profiles of antibody responses to HCoVs differed between countries (UK, Brazil, and Kenya) before and after ChAdOx1 nCoV-19 vaccination.

## Methods

### Study design

This study presents an exploratory immunological analysis using stored serum samples from healthy volunteers enrolled into single-blind, randomised controlled trials of ChAdOx1 nCoV-19. Volunteers were aged 6–≥70 in the United Kingdom (COV002, study registration number: NCT04400838 and COV006, study registration number: ISRCTN15638344), 18–69 in Brazil (COV003, study registration number: ISRCTN89951424) and 18–55 in Kenya (COV004, study registration number: PACTR202005681895696). Briefly, participants were randomised using full allocation concealment with an online randomisation system, to receive either 2 doses of ChAdOx1 nCoV-19 or a control vaccine. This was carried out using REDCap v12.0.19 which was used for the electronic case report form. Randomisation lists were generated by the study statisticians, using block randomisation to take into account study group and site. Study staff enrolled volunteers and administered the vaccinations, however volunteers were blinded to their allocations. CONSORT diagrams for each trial as well as study protocols have previously been published (links provided).^4^^,^^28^, ^29^, ^30^

For this analysis, serum samples were selected from participants who received a standard dose (5 × 10^10^ viral particles) of ChAdOx1 nCoV-19 intramuscularly, four weeks apart, at D0 and D28 and had blood sampling conducted on D0, D28 and D56. A subset of samples came from volunteers who received two standard doses 3 months apart (6–11 years, COV006), as this was the only dosing interval at which this age group received vaccinations in the trial setting. The following numbers of volunteers from each trial contributed samples that were available for inclusion to our analyses in this paper: COV002: 128/10,811, COV003: 99/10,416, COV004: 92/400, COV006: 100/262. Final numbers analysed varied by assay and are detailed in Table 1, including volunteer demographics.

**Table 1:** Healthy volunteer demographics for all assays involved in the study.

Age group comparisons within the UK							
**Assay**	**Time points**	Age group	6–11	12–17	18–55	56–69	≥70
		Age (years): median (IQR)	9 (8–10)	15 (13–16)	40 (31–48)	60 (57–62)	73 (71–75)
		Total N	30	37	40	30	40
		Sex: Male N (%)	13 (43.3)	21 (56.8)	22 (55.0)	14 (46.7)	21 (52.5)
		Sex: Female N (%)	17 (56.7)	16 (43.2)	18 (45.0)	16 (53.3)	19 (47.5)
**Antibody response assays**							
SCoV2 IgG	D0, D28: all; D56: all except 6–11	Age (years): median (IQR)	9 (8–10)	15 (13–16)	41 (34–48)	60 (57–62)	73 (71–75)
		Total N	30	37	39	29	40
		Sex: Male N (%)	13 (43.3)	21 (56.8)	21 (53.2)	13 (44.8)	21 (52.5)
		Sex: Female N (%)	17 (56.7)	16 (43.2)	18 (46.2)	16 (51.2)	19 (47.5)
SCoV2 IgG1	D28 and D56	Age (years): median (IQR)	NA	14 (13–16)	41 (34–48)	60 (57–62)	73 (71–75)
		Total N		29	39	29	40
		Sex: Male N (%)		16 (55.2)	21 (53.2)	13 (44.8)	21 (52.5)
		Sex: Female N (%)		13 (44.8)	18 (46.2)	16 (51.2)	19 (47.5)
SCoV2 IgG3	D28 and D56	Age (years): median (IQR)	NA	15 (13–16)	41 (34–48)	59 (57–61)	73 (71–75)
		Total N		24	39	27	40
		Sex: Male N (%)		16 (66.7)	21 (53.2)	11 (40.7)	21 (52.5)
		Sex: Female N (%)		8 (33.3)	18 (46.2)	16 (59.3)	19 (47.5)
SCoV2 IgA	D28 and D56	Age (years): median (IQR)	NA	14 (13–16)	41 (34–48)	60 (57–62)	73 (71–75)
		Total N		29	39	29	40
		Sex: Male N (%)		16 (55.2)	21 (53.2)	13 (44.8)	21 (52.5)
		Sex: Female N (%)		13 (44.8)	18 (46.2)	16 (51.2)	19 (47.5)
SCoV2 IgM	D28 and D56	Age (years): median (IQR)	NA	15 (13–16)	41 (34–48)	60 (57–62)	73 (71–75)
		Total N		30	39	29	40
		Sex: Male N (%)		16 (53.3)	21 (53.2)	13 (44.8)	21 (52.5)
		Sex: Female N (%)		14 (46.7)	18 (46.2)	16 (51.2)	19 (47.5)
HCoVs IgG (OC43, HKU1, 229E, NL63)	D0, D28: all; D56: all except 6–11	Age (years): median (IQR)	9 (8–10)	15 (13–16)	41 (34–48)	60 (57–62)	73 (71–75)
		Total N	30	37	39	29	40
		Sex: Male N (%)	13 (43.3)	21 (56.8)	21 (53.2)	13 (44.8)	21 (52.5)
		Sex: Female N (%)	17 (56.7)	16 (43.2)	18 (46.2)	16 (51.2)	19 (47.5)
**Antibody functionality assays**							
SCoV2 IgG avidity	D28 and D56	Age (years): median (IQR)	NA	14 (13–16)	41 (32–48)	59 (57–61)	73 (71–75)
		Total N		22	37	23	35
		Sex: Male N (%)		10 (45.5)	20 (54.1)	8 (34.8)	20 (57.1)
		Sex: Female N (%)		12 (54.5)	17 (45.9)	15 (65.2)	15 (42.9)
SCoV2 IgG FcR binding (FcgRIIa, IIb, IIIa, IIIb)	D28 and D56	Age (years): median (IQR)		15 (13–16)	43 (38–50)	61 (57–63)	72 (71–75)
		Total N		23	20	20	20
		Sex: Male N (%)		12 (52.2)	11 (55.0)	8 (40.0)	8 (40.0)
		Sex: Female N (%)		11 (47.8)	9 (45.0)	12 (60.0)	12 (60.0)
HCoV IgG FcR binding (FcgRIIa, IIb, IIIa, IIIb)	D0	Age (years): median (IQR)		15 (13–16)	43 (37–50)	61 (60–64)	73 (71–75)
		Total N		23	10	10	10
		Sex: Male N (%)		12 (52.2)	3 (30.0)	4 (40.0)	2 (20.0)
		Sex: Female N (%)		11 (47.8)	7 (70.0)	6 (60.0)	8 (80.0)
HCoV IgG FcR binding (FcgRIIa, IIb, IIIa, IIIb)	D28	Age (years): median (IQR)		15 (13–16)	44 (37–50)	61 (57–63)	72 (71–75)
		Total N		23	19	20	20
		Sex: Male N (%)		12 (52.2)	10 (52.6)	8 (40.0)	8 (40.0)
		Sex: Female N (%)		11 (47.8)	9 (47.4)	12 (60.0)	12 (60.0)
HCoV IgG FcR binding (FcgRIIa, IIb, IIIa, IIIb)	D56	Age (years): median (IQR)		15 (13–16)	44 (37–50)	61 (57–63)	72 (71–75)
		Total N		up to 23^a^	19	20	20
		Sex: Male N (%)		12 (52.2)	10 (52.6)	8 (40.0)	8 (40.0)
		Sex: Female N (%)		11 (47.8)	9 (47.4)	12 (60.0)	12 (60.0)

Age group comparisons in different countries							
**Assay**	**Time points**	Country	UK		Brazil		Kenya
		Age group	18–55	56–69	18–55	56–69	18–55
		Age (years): median (IQR)	40 (31–48)	60 (57–62)	32 (27–42)	63 (61–67)	27 (24–34)
		Total N	40	30	82	17	92
		Sex: Male N (%)	22 (55.0)	14 (46.7)	32 (39.0)	5 (29.4)	78 (84.8)
		Sex: Female N (%)	18 (45.0)	16 (53.3)	50 (61.0)	12 (70.6)	14 (15.2)
**Antibody response assays**							
SCoV2 IgG	D0 and D28	Age (years): median (IQR)	40 (31–48)	60 (57–62)	32 (27–42)	63 (61–67)	27 (24–34)
		Total N	40	30	82	17	92
		Sex: Male N (%)	22 (55.0)	14 (46.7)	32 (39.0)	5 (29.4)	78 (84.8)
		Sex: Female N (%)	18 (45.0)	16 (53.3)	50 (61.0)	12 (70.6)	14 (15.2)
HCoVs IgG (OC43, HKU1, 229E, NL63)	D0 and D28	Age (years): median (IQR)	40 (31–48)	60 (57–62)	32 (27–42)	63 (61–67)	27 (24–34)
		Total N	40	30	82	17	92
		Sex: Male N (%)	22 (55.0)	14 (46.7)	32 (39.0)	5 (29.4)	78 (84.8)
		Sex: Female N (%)	18 (45.0)	16 (53.3)	50 (61.0)	12 (70.6)	14 (15.2)
**Antibody functionality assays**							
SCoV2 IgG avidity	D28	Age (years): median (IQR)	42 (35–48)	59 (57–61)	32 (27–42)	64 (61–67)	27 (24–34)
		Total N	36	24	82	14	89
		Sex: Male N (%)	20 (55.6)	9 (37.5)	32 (39.0)	3 (21.4)	76 (85.4)
		Sex: Female N (%)	16 (44.4)	15 (62.5)	50 (61.0)	11 (78.6)	13 (14.6)

a FcR 2a: HKU1: n = 20, M (%)/F (%): 12 (60.0)/8 (40.0); 229E: n = 16, M (%)/F (%): 11 (68.8)/5 (31.2); NL63: n = 19, M (%)/F (%): 12 (63.2)/7 (36.8); FcR 2b: OC43: n = 22, M (%)/F (%): 12 (54.5)/10 (45.5); HKU1: n = 20, M (%)/F (%): 12 (60.0)/8 (40.0); 229E: n = 18, M (%)/F (%): 12 (66.7)/6 (33.3); NL63: n = 17, M (%)/F (%): 12 (70.6)/5 (29.4).

Volunteers were grouped by age in years; 6–11, 12–17, 18–55, 56–69, and ≥70 age group and samples were included based on availability across defined time points (D0, D28, and D56). This exploratory analysis aimed to characterise humoural immune responses including: 1) binding IgG against SCoV2 spike, and seasonal HCoVs spike (OC43, HKU1, 229E, and NL63); 2) IgG avidity towards SCoV2 spike; 3) isotype and subclasses profiling to SCoV2 spike and 4) low affinity FcγR (IIa, IIIa, IIb, IIIb) binding levels specific to SCoV2 IgG and HCoVs IgG. The 6–11 age group did not receive a second dose of ChAdOx1-nCoV19 at D28, therefore only limited number of readouts were obtained for this group. Similarly, volunteers from the Kenya cohort did not receive a second dose at D28 and therefore data across countries were only analysed following the first dose.

### Meso Scale Discovery V-PLEX COVID-19 serology

IgG titres against SCoV2 and HCoVs were measured using Meso Scale Discovery (MSD) V-PLEX COVID-19 serology kit (V-PLEX COVID-19 Coronavirus Panel 3 Kit, cat #: K15399U) as per manufacturer's instructions. Briefly, plates were blocked for 30 min at room temperature (RT) with shaking (700 rpm). This was followed by three washes with phosphate buffered saline + 0.05% Tween-20 (PBS-T). The serum standard and controls were plated as directed by the manufacturer while samples were plated in duplicate at dilutions between 1:500 and 1:250,000, diluted with MSD Dilution Buffer. Following a 2 h sample incubation at room temperature with shaking, plates were washed three times with PBS-T followed by the addition of MSD SULFO-TAG™ IgG detection antibody. Plates were covered with foil and incubated for a further 1 h before washing with PBS-T three times and once with PBS. MSD Gold Read Buffer was added, and plates were read immediately on the MESO QuickPlex SQ 120MM plate reader with Methodical Mind software (version™ 2.0.15). Raw plate data were exported to MSD Discovery Workbench (version 4.0) for analysis. Mean fluorescence values (MFV) were converted by the software to AU/ml by multiplying the MFV by the serum dilution.

### Isotype and subclass standardised ELISA

Standardised in-house ELISAs were used to quantify circulating SCoV2 spike-specific IgG1, IgG3, IgA, and IgM responses. Full methodological details for this assay were previously published.^11^ Briefly, 96-well flat-bottom Nunc maxisorb immuno plates (ThermoFisher Scientific, cat #: 442404) were coated overnight with either 2 μg/ml (IgG1, IgG3, IgM) or 5 μg/ml (IgA) of SCoV2 full-length spike protein (Native Antigen, cat #: REC31966). After blocking with casein (ThermoFisher Scientific, cat #: 37528) for 1 h at RT, samples (minimum 1:50 dilution) were plated in triplicate and incubated for 2 h at 37 °C with shaking (300 rpm). Standard curves and internal controls were created from reference serum using a pool of high-titre donor serum. An alkaline phosphatase-conjugated secondary antibody (IgG1, IgG3, IgA, and IgM, SouthernBiotech, cat #: 9052-04, 9210-04, 2050-04, and 2020-04, respectively) was then added and incubated for 1 h at 37 °C with shaking. Plates were developed using PNPP alkaline phosphatase substrate (Sigma–Aldrich, cat #: N2765) for 1–4 h at 37 °C with shaking and read at 405 nm when the internal control reached an OD of 1.

### Avidity ELISA

Anti-SCoV2 spike IgG avidity was assessed using an in-house ELISA. 96-well flat-bottom Nunc maxisorb immuno plates (ThermoFisher Scientific, cat #: 442404) were coated with 1 μg/ml SCoV2 spike protein (Native Antigen, cat #: REC31966) overnight. Plates were washed with PBS-T followed by blocking with casein for 1 h. Serum samples were diluted in casein so that the antibody titre in each sample was equivalent to 1 AU (as determined by our in-house anti-SCoV2 IgG ELISA^11^) to remove the influence of antibody titre on antibody avidity. Samples were plated in duplicate for each test condition and the plates were incubated at RT for 2 h. Plates were washed as above and followed by the addition of PBS or 1.6M NaSCN (Sigma–Aldrich, cat #: 80518) to the appropriate wells and left at RT for 15 min. Plates were washed as above followed by 1 h incubation with a 1:1000 dilution of anti-human IgG-AP detection antibody (Sigma–Aldrich, cat #: A3187). Following a final wash step, PNPP detection substrate was added to each well and the plate was read continuously until each samples PBS only control wells reached an OD405 of 1. Antibody avidity is described as the % of antibody binding following treatment with NaSCN and is calculated as: (average OD405 of wells with NaSCN/average OD405 of wells with PBS) × 100.

### Multiplexed immunoassay

Binding profiles of low affinity FcγR to antigen specific IgG were generated using a custom magnetic multiplexed immunoassay. Antigens, SCoV2 full-length spike protein (Native Antigen, cat #: REC31966), HCoV-OC43 full-length spike protein (Native Antigen, cat #: REC31877), HCoV-HKU1 full-length spike protein (Native Antigen, cat #: REC31867), HCoV-229E full-length spike protein (Native Antigen, cat #: REC31880), and HCoV-NL63 full-length spike protein (Native Antigen, cat #: REC31879) were coupled to Bio-Plex Pro Magnetic COOH beads (Bio-Rad, cat #: 171150600) using the Bio-Rad Bio-Plex Amine Coupling Kit (cat #: 171406001). Using a black, clear-bottom 96-well plate (Greiner, cat #: 655096), 1000 beads per bead region and samples (minimum 1:100 dilution) were added per well. Standard curves and internal controls were created from reference serum using a pool of high-titre donor serum. The plate was covered and incubated at 37 °C on a shaker at 700 rpm for 1 h and was then washed with 0.1% bovine serum albumin in phosphate buffered saline with 0.05% Tween-20. FcγR (IIa, IIIa, IIb, IIIb, SinoBiological, cat #: 10374-H27H-B, 10389-H27H-B, 10259-H27H-B, 11046-H27H-B) bound to antigen-specific IgG were detected using streptavidin-phycoerthrin (SA-PE) (Life Technologies, cat #: S866). After incubation at RT for 30 min on a shaker (700 rpm), the plate was washed before the beads were resuspended in 50 μl of sheath fluid (Life Technologies, cat #: MPXDF4PK). The plate was then incubated at RT for 10 min on a shaker (700 rpm) before being read by the MAGPIX® System with xPONENT software (version 4.3.229.0). The binding of the PE-detectors was measured to calculate the median fluorescence intensity (MFI). MFI values were fitted to a 4-Parameter logistic model standard curve. Test sera arbitrary units (AU) were calculated from their MFI values using the parameters estimated from the standard curve.

### Ethics

Studies were conducted in accordance with the principles of the Declaration of Helsinki and Good Clinical Practice. Written informed consent was obtained from all volunteers over the age of 16. Written assent was obtained from volunteers over the age of 11. Written consent was obtained from parents of guardians of volunteers aged between 6 and 15 years. Studies were approved by the following committees: COV002/COV006: Medicines and Healthcare products Regulatory Agency (MHRA) (reference 21584/0428/001-0001 and 21584/0441/001-0001) and South-Central Berkshire Research Ethics Committee (reference 20/SC/0179 and 20/SC/0054); COV003: Brazilian National Research Ethics Committee (reference 4068113), Oxford Tropical Research Ethics Committee (OxTREC Reference 36-20); COV004: Kenya Medical Research Institute (KEMRI) Scientific and Ethics Research Unit (KEMRI/SERU/CGMR-C/CSC197/4024), Kenya Pharmacy and Poisons Board (ECCT/20/05/01), National Commission for Science, Technology and Innovation (NACOSTI/P/22/20461), and University of Oxford Tropical Research Ethics Committee (Reference 33-20).

### Statistics

The datasets were tested for normality with the Shapiro–Wilk test. Data were presented as medians with interquartile ranges (IQR). Median fold changes between time points were calculated for each age group with 95% confidence intervales (CI), estimated using the binomial distribution. For comparisons between age groups median differences were calculated with 95% CI, estimated by bootstrapping with 10,000 resamples. Differences between age groups within a single time point were analysed using the Kruskal–Wallis test for multiple comparisons, or the Mann–Whitney U test for two-group comparisons. Bonferroni correction was used to control for type I errors. Linear regression was used to explore the association between age as a continuous variable and log-transformed IgG. Assumptions underlying the ordinary least squares (OLS) regression (linearity, homogeneity of variance and normality of residuals) were graphically assessed and confirmed. The normality assumption underlying correlation analysis has been graphically assessed using Q-Q plots. Pearson correlations were reported for linear relationships with normal distributions; Spearman correlations were used otherwise. Pearson and Spearman correlation coefficients asymptotically follow a Student's t-distribution with (n−2) degrees of freedom, and their 95% CI are derived from quantiles of this distribution. There was no formal sample size calculation for immunogenicity outcomes showed here, as these were considered secondary or exploratory objectives within the context of the larger clinical trials (COV002, COV003, COV004, and COV006).^4^^,^^28^, ^29^, ^30^ Although no formal sample size calculation was performed, the adequacy of the sample size was informally assessed by evaluating the precision of the estimates, focussing on the width of the 95% CI. All statistical analyses and figure generation were performed using GraphPad Prism version 10.2.23 except calculations of median difference with 95% CI that was performed in Python (version 3.12.7).

### Role of funders

Funders had no role in study design, data collection, data analyses, interpretation, or writing of this report.

## Results

### Age-related differences in antibody responses demonstrated after the first dose of ChAdOx1 nCoV-19

**Fig. 1:**
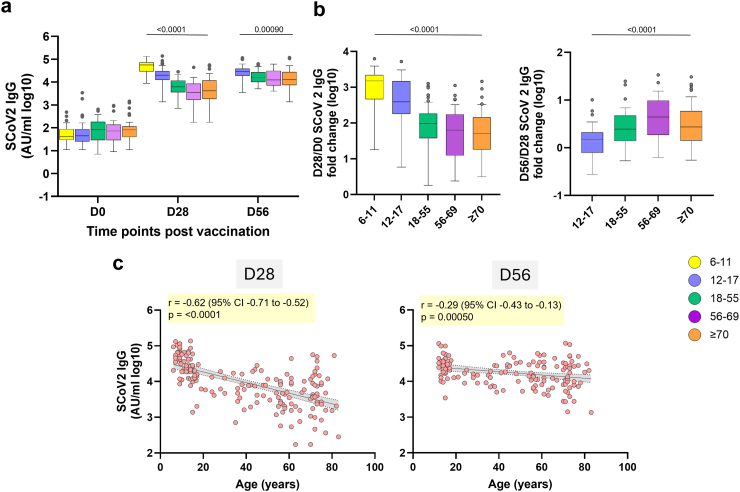
**Comparison of binding IgG to SCoV2 spike between age groups following one and two doses of ChAdOx1-nCoV19.** (a) SCoV2 IgG at baseline (D0), 28 days post first (D28), and second dose (D56) of ChAdOx1 nCoV19 was quantified using the MSD platform. (b) Fold change in IgG 28 days post-first dose (D28/D0, left) and 28 days post-second dose (D56/D28, right) of ChAdOx1 nCoV19. Box plots display the median (midline), the 25th and 75th percentiles (box), and the whiskers represent the minimum and maximum values within 1.5 times the IQR from Q1 and Q3. Outliers, defined as values exceeding 1.5 times the IQR, are shown as individual points. Kruskal–Wallis was used to assess differences between age groups at each time point (p values are shown). Age groups: 6–11, n = 30; 12–17, n = 37; 18–55, n = 39; 56–69, n = 29 and ≥70, n = 40. (c) Correlation of SCoV2 IgG with age as a continuous variable at D28 (n = 175) and D56 (n = 145). Each dot is one participant while solid lines indicate the linear regression with 95% CIs presented as the shaded area. Pearson correlation coefficients (r) are shown with 95% CIs. ∗Note: 6–11 age group did not receive a second dose of ChAdOx1-nCoV19 at D28, therefore IgG was not quantified at D56.

One dose of ChAdOx1 nCoV-19 induced higher levels of SCoV2 IgG in 6–11 years age group compared to older age groups. For example, the median difference between 6 and 11 and ≥70 years age groups was 51,785 AU/ml (95% CI: 34,416–60,803 AU/ml), and the corresponding fold change from baseline was 1512-fold (95% CI: 721–1964-fold) in 6–11, versus 51.6-fold (95% CI: 34.2–113-fold) in ≥70 years age group–representing a nearly 30-fold greater relative increase in the younger group. Similar differences were observed when comparing 6–11 year olds with the 18–55 and 56–69 age groups, both in terms of absolute antibody levels and fold increases (Fig. 1a and b, Supplementary Table S1). The 12–17 years age group also showed higher responses than adults, though to a lesser extent (e.g., vs ≥70: median difference 15,925 AU/ml, 95% CI: 10,999–22,895 AU/ml; fold change: 392-fold, 95% CI: 236–753-fold). This large magnitude of difference highlights the stronger capacity for de novo antibody generation in children and adolescents.

**Supplementary Fig. S1:**
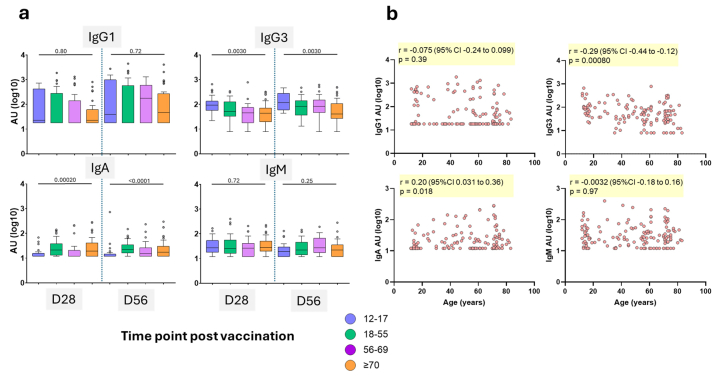
**Comparison of antibody isotype and subclass responses to SCoV2 spike between age groups following one and two doses of ChAdOx1 nCoV19**. (a) IgG1, IgG3, IgA and IgM responses to SCoV2 spike were quantified at 28 days post first (D28) and post second (D56) dose of ChAdOx1 nCoV19 using in-house ELISAs. Box plots display the median (midline), the 25th and 75th percentiles (box), and the whiskers represent the minimum and maximum values within 1.5 times the IQR from Q1 and Q3. Outliers, defined as values exceeding 1.5 times the IQR, are shown as individual points. Kruskal-Wallis was used to assess differences between age groups at each time point (p values are shown). Age group IgG1: 12–17, n = 29; 18–55, n = 39; 56–69, n = 27 and ≥70, n = 40; IgG3: 12–17, n = 24; 18–55, n = 39; 56–69, n = 27 and ≥70, n = 40; IgA: 12–17, n = 29; 18–55, n = 39; 56–69, n = 29 and ≥70, n = 40; IgM: 12–17, n = 30; 18–55, n = 39; 56–69, n = 29 and ≥70, n = 40. (b) Correlation of SCoV2 IgG1, IgG3, IgA and IgM titres with age as a continuous variable at D28. Each dot is one participant. Spearman correlation coefficients (r) are shown with 95% CIs. IgG1, IgA n = 137, IgG3 n = 130 and IgM n = 138.

After the second dose, SCoV2 IgG levels remained the highest in the 12–17 years age group compared with the older age groups (e.g., median difference vs ≥70: 16,258 AU/ml, 95% CI: 3136–23,744 AU/ml). However, the greatest increase in the D56/D28 fold change was seen in adults, particularly in the 56–69 age group (Fig. 1b, Supplementary Table S1). Consistent with the group-wise trends, SCoV2 IgG levels were negatively associated with age (D28 Pearson r = −0.62, 95% CI: −0.71 to −0.52; D56 Pearson r = −0.29, 95% CI: −0.43 to −0.13) (Fig. 1c). Vaccine-induced SCoV2 IgG response had mixed IgG1 and IgG3 profile across all volunteers (Supplementary Fig. S1a, Supplementary Table S1). SCoV2 IgG3 responses decreased with age, while SCoV2 IgA showed modest increase in older age groups (Supplementary Fig. S1b).

**Fig. 2:**
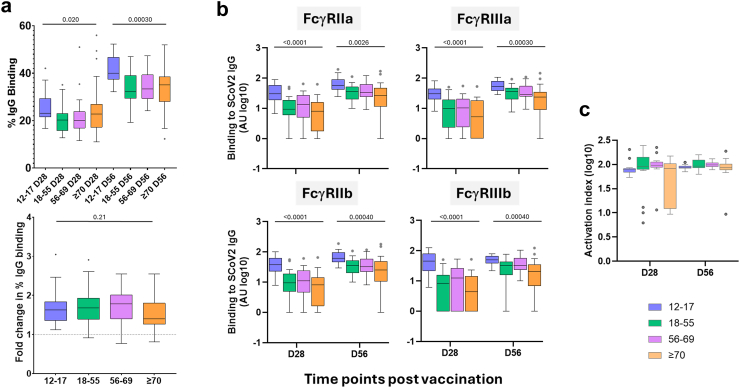
**SCoV2 IgG avidity and FcγR binding levels comparison between age groups following one and two doses of ChAdOx1 nCoV19.** (a) SCoV2 IgG avidity was assessed at D28 and D56 using an in-house avidity ELISA by calculating the percentage of antibody binding following exposure to NaSCN (top). The fold change in avidity following first and second vaccinations was calculated (bottom). Age groups: 12–17, n = 22; 18–55, n = 37; 56–69, n = 22, and ≥70, n = 35. (b) Binding levels of low affinity FcγRIIa, FcγRIIb, FcγRIIIa, and FcγRIIIb to SCoV2 IgG were determined by an in-house multiplex bead-based assay. Age groups: 12–17, n = 23; 18–55, n = 19; 56–69, n = 20 and ≥70, n = 20. (c) FcγR activation index was calculated as log transformed FcγRIIa + FcγRIIIa/FcγRIIb. Age groups: 12–17, n = 23 (both time points); 18–55, n = 17 (D28), n = 19 (D56); 56–69, n = 16 (D28), n = 20 (D56) and ≥70, n = 15 (D28), n = 18 (D56). Box plots display the median (midline), the 25th and 75th percentiles (box), and the whiskers represent the minimum and maximum values within 1.5 times the IQR from Q1 and Q3. Outliers, defined as values exceeding 1.5 times the IQR, are shown as individual points. Kruskal–Wallis was used to assess differences between age groups at each time point (p values are shown).

SCoV2 IgG avidity after one dose of ChAdOx1 nCoV-19 showed trend of higher median values in the 12–17 years age group when compared with older age groups, although these differences were modest (median difference vs 18–55: 2.8% binding, 95% CI: 0.7–9.2% binding; vs 56–69: 3.1% binding, 95% CI: −0.7 to 8.5% binding; vs ≥70: 0.3% binding, 95% CI: −2.0 to 7.7% binding) (Fig. 2a, top panel, Supplementary Table S2). After the second dose, avidity increased in all age groups, with more pronounced differences between the 12–17 age group and older adults (median difference vs 18–55: 7.8% binding, 95% CI: 4.6–11.1% binding; vs 56–69: 6.7% binding, 95% CI: 2.3–11.1% binding; vs ≥70: 4.9% binding, 95% CI: 2.4–10.0% binding) (Fig. 2a, top panel, Supplementary Table S2). The fold increase in IgG avidity (D56/D28) was similar across all age groups (Fig. 2a, bottom panel, Supplementary Table S2). This indicates that although younger individuals may have higher avidity especially after the second dose, subsequent dose lead to proportionate increases across all age groups.

After one dose of ChAdOx1 nCoV-19, binding of FcγRIIa, FcγRIIIa, FcγRIIb, and FcγRIIIb to SCoV2 IgG was highest in the 12–17 years age group (Fig. 2b, Supplementary Table S2). For FcγRIIa, the median difference between 12–17 and ≥70 years age group was 22.5 AU (95% CI: 10.4–40.2 AU), and for FcγRIIIa it was 25.3 AU (95% CI: 12.0–32.8 AU), indicating strong differences in activating receptor binding. Similarly, the 12–17 group showed higher levels of FcγRIIb binding (median difference vs ≥70: 29.0 AU, 95% CI: 19.2–43.0 AU) and FcγRIIIb binding (median difference vs ≥70: 40.8 AU, 95% CI: 18.6–76.3 AU), supporting robust overall FcγR binding in this age group. Comparable differences were also observed when comparing the 12–17 year olds with the 18–55 and 56–69 age groups across all receptors, with consistently higher FcγR binding in adolescents. In contrast, comparisons among adult age groups revealed smaller and uncertain differences across all receptors, with most confidence intervals including zero (e.g., FcγRIIIa: 18–55 vs ≥70 median difference 4.8 AU, 95% CI: −4.1 to 13.3 AU). This suggests that while FcγR binding responses are stronger in 12–17 years age group, they are relatively comparable among older adults, indicating stronger antibody effector functions in younger individuals. These trends were consistent across all four FcγR (Fig. 2b, Supplementary Table S2). The trends observed after the first dose were maintained following the second vaccination, with 12–17 year olds consistently exhibiting higher FcγR binding across all receptors. While overall FcγR engagement increased post-boost, the relative differences between age groups remained comparable (Fig. 2b, Supplementary Table S2). The FcγR activation index, which compares activating to inhibitory receptors showed greater variability in the oldest age group after the first dose. This variability was reduced following the second dose, with narrower confidence intervals across all age groups, suggesting that boosting is critical for optimal FcγR binding and antibodies effector function in older individuals (Fig. 2c, Supplementary Table S2).

### ChAdOx1 nCoV-19 vaccination induces more cross-reactive antibodies to HCoVs in youngest age group

**Fig. 3:**
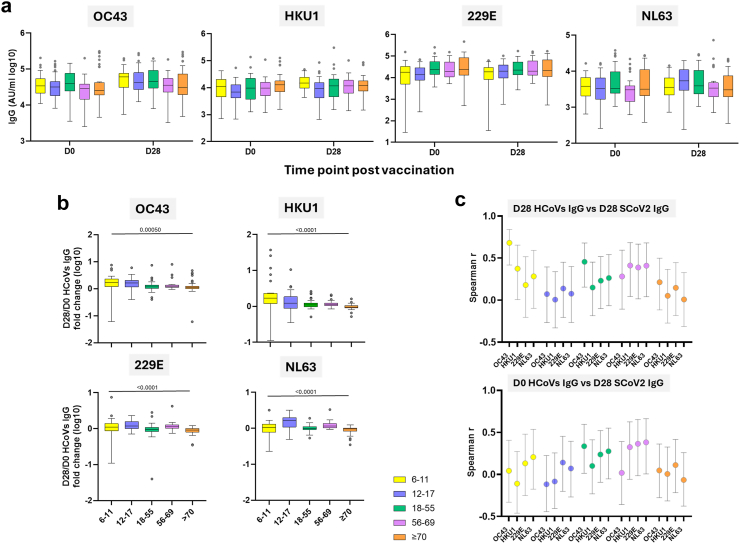
**IgG responses to HCoVs (OC43, HKU1, 229E, and NL63) between age groups following one dose of ChAdOx1 nCoV19.** (a) Comparison of IgG responses to HCoVs at baseline (D0) and post-first dose (D28) of ChAdOx1 nCoV19 between age groups. Responses were quantified using the MSD platform. (b) Fold change in HCoVs IgG following ChAdOx1 nCoV19 vaccination (D28/D0). Box plots display the median (midline), the 25th and 75th percentiles (box), and the whiskers represent the minimum and maximum values within 1.5 times the IQR from Q1 and Q3. Outliers, defined as values exceeding 1.5 times the IQR, are shown as individual points. Kruskal–Wallis was used to assess differences between age groups at each time point (p values are shown). (c) Spearman correlation coefficient (circles) of D28 HCoVs and D28 SCoV2 IgG responses (top) and baseline HCoVs and D28 SCoV2 IgG responses (bottom) with error bars presenting 95% CI. Age groups: 6–11, n = 30; 12–17, n = 37; 18–55, n = 39; 56–69, n = 29 and ≥70, n = 40.

**Supplementary Fig. S2:**
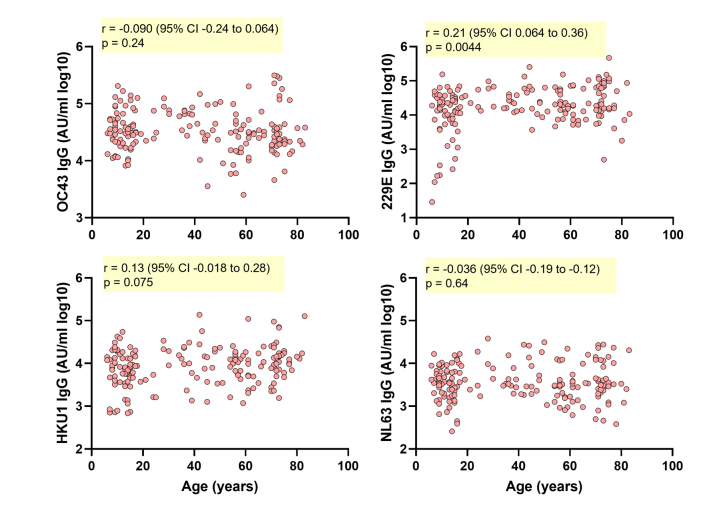
**Correlation of HCoVs at D0 with age as a continuous variable (n = 175).** Each dot is one participant. Spearman correlation coefficients (r) are shown with 95% CIs.

Age-related differences in immune response to one dose of ChAdOx1 nCoV-19 could imply variation in pre-existing immunity towards HCoVs. Before vaccination, there were only minor differences in HCoVs IgG titres across age groups and substantial variability was present within each group (Fig. 3a, Supplementary Table S3). Correlations between baseline HCoVs IgG and age were weak or absent (OC43 Spearman r = −0.090, 95% CI: −0.24 to −0.064; HKU1 Spearman r = 0.13, 95% CI: −0.018 to 0.28; 229E Spearman r = 0.21, 95% CI: 0.064–0.36; NL63 Spearman r = −0.036, 95% CI: −0.19 to −0.12), indicating that pre-vaccination antibody levels were not strongly associated with age (Supplementary Fig. S2).

ChAdOx1 nCoV-19 vaccination induced modest but detectable increases in HCoVs IgG. The strongest fold increases were observed in the 6–11 and 12–17 year groups, particularly for OC43 and HKU1. For example, OC43 fold change in 6–11 years was 1.68-fold (95% CI: 1.37–2.07-fold) and in 12–17 years 1.64-fold (95% CI: 1.30–1.81-fold), compared with 1.15-fold (95% CI: 1.06–1.31-fold) in 18–55, 1.22-fold (95% CI: 1.13–1.34-fold) in 56–69, and 1.09-fold (95% CI: 1.02–1.11-fold) in ≥70 years age group. A similar age-related trend was seen for HKU1, where fold increases declined from 1.71-fold (95% CI: 1.27–2.11-fold) in 6–11 to 0.96-fold (95% CI: 0.93–1.02-fold) in ≥70 years age group. For 229E and NL63, the 12–17 year group showed the most pronounced increases (fold change 229E: 1.17-fold, 95% CI: 1.10–1.45; fold change NL63: 1.63-fold, 95% CI: 1.30–1.89-fold), while adult responses remained lower or near baseline (Fig. 3b, Supplementary Table S3). Although fold changes were >1.0-fold in most groups, increases in HCoVs IgG were modest in older adults while larger fold changes in children and adolescents would suggest a more dynamic cross-reactive response. No meaningful increase in HCoVs IgG was observed following the second dose of ChAdOx1 nCoV-19 with D56/D28 fold changes close to or below 1.0-fold across all age groups and HCoVs, indicating a lack of further antibody boosting (Supplementary Table S3). These findings are compatible with vaccine-induced enhancement of cross-reactive HCoVs IgG occurring primarily after the first dose.

To explore potential cross-reactivity, we examined the relationship between HCoVs IgG and SCoV2 IgG both at D28. Beta HCoVs IgG and SCoV2 IgG were strongly positively correlated in volunteers aged 6–11 years, particularly for OC43 (Spearman r = 0.68, 95% CI: 0.42–0.84). Additionally, moderate positive associations were detected in the 18–55 age group for OC43 (Spearman r = 0.46, 95% CI: 0.16–0.68) and in the 56–69 age group for HKU1, 229E, and NL63 (Spearman r range: 0.39–0.41, 95% CI range: 0.014–0.68) (Fig. 3c, upper panel, Supplementary Table S4). When analysing baseline HCoVs IgG versus SCoV2 IgG at D28, only weak associations were observed (18–55: OC43 Spearman r = 0.34, 95% CI: 0.014–0.60; 56–69: HKU1, 229E, and NL63 Spearman r range: 0.33–0.38, 95% CI range: −0.058 to 0.66) (Fig. 3c, lower panel, Supplementary Table S4). No meaningful correlations were observed in the 12–17 or ≥70 years groups for either D28 HCoVs vs D28 SCoV2 IgG levels or baseline HCoVs IgG vs D28 SCoV2 responses (Fig. 3c, Supplementary Table S4). Collectively, these findings suggest that pre-existing or co-induced HCoVs IgG may shape the magnitude of the SCoV2 IgG response following vaccination. However, these associations were not consistent across all age groups or HCoVs, indicating that age and immune history of specific HCoVs exposure likely influence the degree of cross-reactivity.

**Fig. 4:**
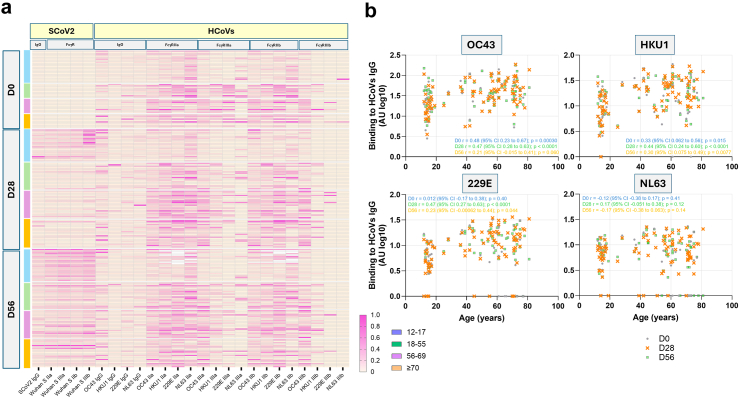
**Relationship of FcγR binding levels to SCoV2 IgG and HCoVs IgG.** (a) Heat-map of min-max normalised data for SCoV2 IgG, FcγR binding levels to SCoV2 IgG, HCoVs IgG and FcγR binding levels to HCoVs IgG. Each row represents data from one individual within the different age groups (colour indicated in the figure legend on the right), across different HCoVs antigens during course of vaccination (D0, D28, and D56) with ChAdOx1 nCoV19. White areas represent the missing data. (b) Correlation of FcγRIIa binding levels across different HCoVs with age, each dot is one participant at different time points (D0, D28, and D56). Spearman correlation coefficients (r) are shown with 95% CIs. Age groups at D0: 12–17, n = 23; 18–55, n = 10; 56–69, n = 10 and ≥70, n = 10; D28: 12–17, n = 23; 18–55, n = 19; 56–69, n = 20 and ≥70, n = 20; D56: 12–17, n = 23 (except for FcγRIIa 229E n = 16; NL63 n = 19 and for FcγRIIa 229E n = 18; NL63 n = 17); 18–55, n = 19; 56–69, n = 20 and ≥70, n = 20.

Next, we examined if similar age-related differences existed at the level of FcγR binding to HCoVs IgG, which might underpin differences in antibody effector functions. At baseline, the 12–17 years olds had lower FcγR binding to HCoVs IgG in contrast to older age groups (Fig. 4a, Supplementary Table S5). OC43 and HKU1 showed the most prominent increasing age-related trends across FcγRIIa, FcγRIIIa, and FcγRIIb, with Spearman correlation coefficients ranging between 0.33 and 0.48 at D0 and 0.27–0.49 at D28 (D0 95% CI range: 0.062–0.67; D28 95% CI range: 0.052–0.65), indicating that this relationship was maintained after the first dose of the vaccine (Fig. 4b, Supplementary Table S6). After the second dose, there was a decreasing trend in the correlation strength between FcγR binding levels to OC43 and HKU1 IgG and age (Spearman r range for FcγRIIa, FcγRIIIa, and FcγRIIb: 0.21–0.41; 95% CI range: −0.0090 to 0.58) (Supplementary Table S6). In summary, these results show a cross-reactive immune response following ChAdOx1 nCoV-19 vaccine in youngest individuals, particularly for OC43 and HKU1, while older age groups have less pronounced fold changes and higher baseline FcγR binding.

### ChAdOx1 nCoV-19 and HCoVs antibody response across adults from the UK, Brazil, and Kenya

To assess how geographical location impacts HCoVs IgG and potentially the response to ChAdOx1 nCoV-19 vaccination, SCoV2 and HCoVs IgG at D0 and D28 after a single dose of ChAdOx1 nCoV-19 were assessed in healthy adult volunteers enrolled in the UK (COV002), Brazil (COV003), and Kenya (COV004) trials. Volunteers were split into two age groups (18–55 and 56–69 years) to identify any age-related responses within countries. Volunteers from Kenya were limited to the 18–55 years age group.

**Fig. 5:**
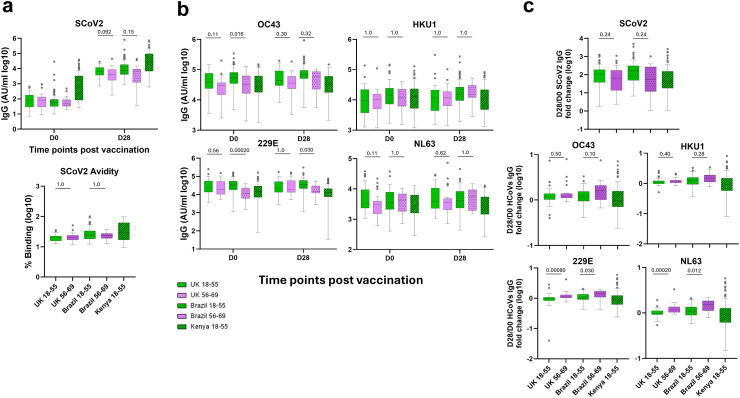
**SCoV2 and HCoVs IgG response in various countries (UK, Brazil, and Kenya) at baseline (D0) and 28 days post first vaccination (D28) with ChAdOx1 nCoV19.** (a) Baseline and D28 SCoV2 IgG measured by MSD (top) and D28 avidity measured by an in-house ELISA (bottom). Avidity is shown as percentage of antibody binding following exposure to NaSCN. (b) Baseline and D28 HCoVs IgG measured by MSD. (c) Fold change in SCoV2 and HCoVs IgG levels 28 days post-first dose (D28/D0) of ChAdOx1-nCoV19. Box plots display the median (midline), the 25th and 75th percentiles (box), and the whiskers represent the minimum and maximum values within 1.5 times the IQR from Q1 and Q3. Outliers, defined as values exceeding 1.5 times the IQR, are shown as individual points. Mann–Whitney test used to determine differences within countries, between age groups. Participant numbers for all analyses except avidity: UK 18–55: 40, 56–69: 30; Brazil 18–55: 82, 56–69: 17; Kenya 18–55: 92. Avidity participant numbers: UK 18–55: 36, 56–69: 24; Brazil 18–55: 82, 56–69: 14; Kenya 18–55: 89.

**Supplementary Fig. S3:**
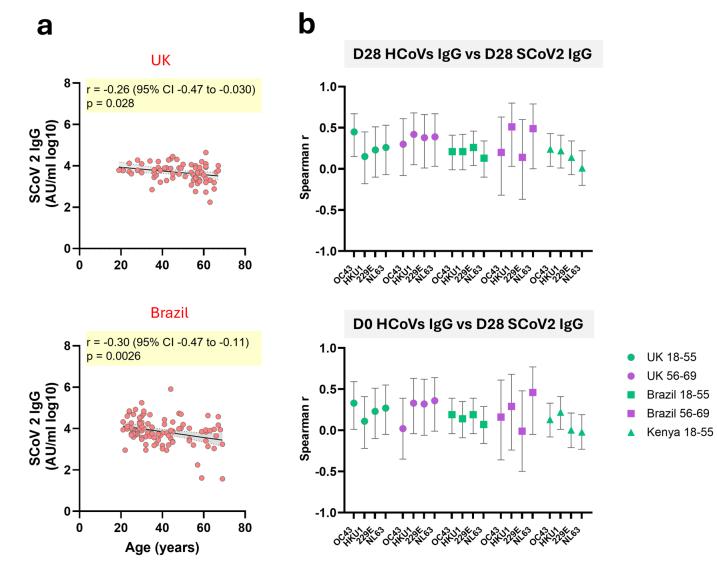
**SCoV2 and HCoVs IgG response in various countries (UK, Brazil and Kenya).** (a) Correlation of SCoV2 IgG with age as a continuous variable at D28 in the UK (top, n = 70) and Brazil (bottom, n = 99). Each dot is one participant while solid lines indicate the linear regression with 95% CIs presented as the shaded area. Pearson correlation coefficients (r) are shown with 95% CIs. (b) Spearman correlation coefficient (symbols) across different countries of D28 HCoVs and D28 SCoV2 IgG responses (top) and baseline HCoVs and D28 SCoV2 IgG responses (bottom) with error bars presenting 95% CI. Participant numbers: UK 18–55: 40, 56–69: 30; Brazil 18–55: 82, 56–69: 17; Kenya 18–55: 92.

At D28 SCoV2 IgG increased from baseline across all countries (Fig. 5a, upper panel, Supplementary Table S7). In the UK, the 18–55 years age group had higher SCoV2 IgG levels at D28 compared to the 56–69 group, with a median difference of 2204 AU/ml (95% CI: −916 to 5174 AU/ml), and a corresponding fold change from baseline of 99.7-fold (95% CI: 65.0–141.9-fold) versus 64.5-fold (95% CI: 18.6–91.3-fold)–representing a 1.5-fold greater relative increase in the younger group. A similar trend was observed in Brazil, where the median difference between the 18–55 and 56–69 age groups was 668 AU/ml (95% CI: −2401 to 7313 AU/ml), and the fold change was 176.5-fold (95% CI: 122.2–216.1-fold) in the younger group compared to 55.7-fold (95% CI: 13.9–247.6-fold) in the older group–representing a 3.2-fold greater relative increase in the younger group. While there were wide intervals around the median differences, the fold change data indicate a pattern consistent with stronger responses in younger adults across both settings. This age-associated trend was further supported by negative correlations between age and SCoV2 IgG levels at D28 in both countries (UK Pearson r = −0.26, 95% CI: −0.47 to −0.030; Brazil Pearson r = −0.30, 95% CI: −0.47 to −0.11) (Supplementary Fig. 3a). Similar levels in avidity towards the SCoV2 spike were observed between age groups and across countries (Fig. 5a, lower panel).

Baseline and post-vaccination HCoVs IgG levels across countries are presented in Fig. 5b. In Brazil, older adults had higher D28/D0 fold changes for all HCoVs compared to younger adults, with median fold changes ranging from 1.43-fold to 1.62-fold (95% CI range: 1.01–2.06-fold) in the older group and 1.05-fold–1.27-fold (95% CI range: 0.95–1.36-fold) in the younger group. In the UK, fold changes were similar across age groups for OC43 and HKU1, but were higher in older adults for 229E and NL63 (e.g., NL63: 1.17-fold, 95% CI: 1.08–1.31-fold in 56–69 vs 0.98-fold, 95% CI: 0.95–1.03-fold in 18–55). In Kenya, fold changes were consistently low across all HCoVs in the 18–55 years group, with values near or below 1.0-fold indicating limited vaccine-induced boosting of HCoVs antibodies in this setting (Fig. 5c, Supplementary Table S7). The overall correlation showed similar profiles between baseline HCoVs IgG and SCoV2 IgG at D28 and between HCoVs IgG and SCoV2 IgG both at D28 (Supplementary Fig. S3b, Supplementary Table S7). Overall, these results suggest that, although levels of pre-existing IgG to HCoV2 varied between countries, this did not strongly influence the overall response profiles to ChAdOx1 nCoV-19 in adult age groups.

## Discussion

We observed a distinct humoural immune response in younger individuals after one dose of ChAdOx1 nCoV-19. Higher levels of vaccine-induced SCoV2 IgG response were observed in both younger age groups (6–11 and 12–17), with higher avidity and higher levels of FcγR binding (12–17 age group). The youngest age group also generated cross-reactive antibodies towards beta HCoVs. In contrast, older age groups had lower levels of vaccine-induced SCoV2 IgG with lower avidity and lower cross-reactivity towards beta HCoVs, and with lower and more variable FcγR binding profiles. Furthermore, older individuals had higher baseline levels of FcγR binding to HCoVs IgG which were maintained after the first dose.

ChAdOx1 nCoV-19 was critical in protecting older individuals from severe disease and hospitalisation, as shown by real-world data.^31^, ^32^, ^33^, ^34^ Our results are consistent with previous studies that have shown higher antibody responses in younger age groups after one dose of a COVID-19 vaccine, whether mRNA or viral-vector based.^28^^,^^35^ These differences between age groups were less pronounced after the second dose.

Longitudinal sero-epidemiological studies have demonstrated that immunity against OC43, HKU1, 229E, and NL63 increases during childhood and is sustained through adulthood.^36^^,^^37^ Natural infection with SCoV2 has been linked to the induction of cross-reactive antibodies in serum and nasal mucosa against OC43 and HKU1 in both children and adults, often referred to as “back-boosting”,^21^^,^^38^^,^^39^ with similar responses found in sera post-vaccination with mRNA-1273.^40^ In our dataset after the first dose of ChAdOx1 in younger individuals, we observed an increase in absolute titres of OC43 and HKU1 IgG, and to a lesser extent 229E and NL63 IgG, indicating a back-boosting effect. In the youngest age group (6–11 years), the greatest fold change of OC43 and HKU1 IgG increase, together with positive correlations between D28 OC43 and HKU1 IgG and D28 SCoV2 IgG, suggests a strong vaccine-induced antibody response that is highly cross-reactive with OC43 and HKU1. In contrast, the OC43 and HKU1 IgG fold increases were less pronounced in older age groups. Further analysis across geographical locations highlighted that although there are differences in baseline HCoVs IgG, there are consistent trends in ChAdOx1 nCoV-19-induced IgG response; younger adults responded to the vaccine with higher levels of SCoV2 IgG compared to older adults.

Antigen-antibody and antibody-FcγR affinity are essential for the formation of stable immune complexes.^41^ Following the first dose of ChAdOx1 nCoV-19, avidity to SCoV2 spike and binding of FcγR to vaccine-induced SCoV2 IgG was more heterogeneous in older adults. While FcγR binding can directly depend on the amount of IgG available, other factors such as glycan structure alterations of the Fc portion could further influence FcγR binding, which is closely associated with ageing.^42^ We show that following the second dose of ChAdOx1 nCoV-19 variability across older age groups is reduced, suggesting that the second vaccine dose enhances both antibody levels and functional responses.

Studies of natural infection with SCoV2 demonstrated that children generate a more polyreactive antibody response due to fewer exposures to HCoVs. In contrast, older adults, with repeated exposures to HCoVs, develop a more specific and higher-affinity antibody pool.^21^ Furthermore, Selva et al. showed distinct humoural profiles in healthy children and the elderly where elderly individuals had increased levels of bound FcγR to HCoVs IgG.^9^ This could explain the higher baseline levels of FcγR binding to HCoVs in older individuals in our study, as their antibodies could be more effective at binding FcγR due to their greater specificity and affinity, even if overall HCoVs IgG levels are similar across age groups.

There are several limitations of this study. It is important to note that although lockdown measures were in place in the UK at the time of sampling, community transmission of HCoVs may nonetheless have occurred during 2020 (for adults) and 2021 (for children aged 6–17 years of age) and could confound the results. Additionally, age-related differences in HCoVs prevalence rates have been observed as well as significant variation between regions.^14^, ^15^, ^16^ Climatic, and socio-economic factors may also contribute to variability. Demographic factors such as sex assigned at birth, race, and ethnicity, may also influence the individual responses. For example, the Brazil cohort was predominantly female, while the Kenya cohort was predominantly male, which may introduce additional confounding effects. However, due to the exploratory nature of the study and small sample size, these variables were not analysed in detail.

Our results demonstrate both quantitative and qualitative differences in the humoural immune response profile in younger and older age groups after vaccination with ChAdOx1 nCoV-19. Importantly these findings are not UK-centric, with similar responses and trends measured from adult trial volunteers in Kenya and Brazil. Older adults are highly susceptible to severe SCoV2 infection. We demonstrate that vaccination with ChAdOx1 nCoV-19 in older age groups improves the ability of SCoV2 IgG to bind FcγR. Importantly, the most pronounced age-related differences were observed after the first dose of ChAdOx1 nCoV-19, with these differences becoming less apparent following the second dose. Investigating if this phenomenon is broadly applicable to the vaccine platform will be critical for assessing the suitability of using one dose regimens in response to outbreaks.

## Contributors

SBR, ES, GLi, NB, SCa, MH, AF, JB, HS, GLip, DW, AAY, SACC, GMW, LYW, MMH, SCh, JNG, DM, HKK, AJP, and TL contributed to study implementation and/or laboratory data acquisition. SBR, ES, GLi, and HS contributed to data analysis and visualisation. SBR, ES, and GLi wrote the original draft. SBR, ES, GLi, MV, CB, KH, RS, SB, AJP, and TL contributed to the editing of the manuscript. All authors read and approved the final version of the manuscript.

## Data sharing statement

Anonymised participant data will be made available following the completion of the trials, upon requests directed to the corresponding author. Proposals will be reviewed and approved by the sponsor, investigator, and collaborators on the basis of scientific merit. After approval of a proposal, data can be shared through a secure online platform after signing a data access agreement. All data will be made available for a minimum of 5 years from the end of the trial.

## Declaration of interests

TL was a consultant to Barinthus Biotherapeutics on an unrelated project involving ChAdOx1 vector vaccines. TL is also named as an inventor on a patent involving unrelated ChAdOx1 vector vaccines. TL also reports an honorarium from Seqirus. SBR, ES, AF, SB, RS, GLi, HS, TL, DW, MV, and AJP are inventors and/or contributors to intellectual property licenced by Oxford University Innovation to AstraZeneca. AJP is chair of DHSCs Joint Committee on Vaccination and Immunisation but did not participate in the JCVI COVID19 vaccine committee during the pandemic, and was a member of the World Health Organisation SAGE until 2022. AJP was chair of WHOs Salmonella TAG until end of 2024 and is a current member. AJP received mRNA material from Moderna to study immunity in preclinical models (unrelated to the current study). AJP undertakes research on behalf of the University of Oxford funded by Serum Institute of India. He also receives grants from the Gates Foundation, Wellcome Trust, CEPI, MRC, NIHR, AstraZeneca, and European Commission as well as consulting for Shinogi and the Ellison Institute, Oxford.

## Appendix A Supplementary data

Supplementary Table S7

COV004_Trial_Protocol

ISRCTN15638344 COV006_Protocol
