# Supplementary material for: Age differences in immunity to human seasonal coronaviruses and the immunogenicity of ChAdOx1 nCoV-19 (AZD1222)
Source: eBioMedicine. 2025 Jul 16;118:105847. doi: 10.1016/j.ebiom.2025.105847 (PMC12284711; doi:10.1016/j.ebiom.2025.105847)
Supplement: COV004_Trial_Protocol [file mmc8.pdf]

KWTRP
CLINICAL TRIAL PROTOCOL
A phase Ib/II single-blinded, randomised, controlled study to determine safety, immunogenicity and efficacy of the candidate Coronavirus Disease (COVID-19) vaccine ChAdOx1 nCoV-19 in adults in Kenya (COV004)

1. GENERAL INFORMATION

**Protocol Number:**	**KEMRI/SERU/CGMR-C/CSC197/4024**
**Trial Registration Number:**	**PACTR202005681895696**
**Investigational Product(s):**	a) ChAdOx1 nCoV-19, a replication-deficient simian adenoviral vector expressing the spike (S) protein of SARS-CoV-2<br>b) Rabies vaccine
**Funders [name and address]:**	**Wellcome Trust,** Gibbs Building, 215 Euston Road, London NW1 2BE, UK<br>Email: grants@wellcome.ac.uk<br>Tel: +44 20 7611888<br><br>**Medical Sciences Division, University of Oxford**, Level 3, John Radcliffe Hospital, Oxford OX3 9DU, UK<br>Email: communications@medsci.ox.ac.uk<br>Tel: +44 1865 289561<br><br>**Innovate UK and Dept of Health and Social Care,** Polaris House, North Star Avenue, Swindon, Wiltshire SN2 1FL, UK<br>Email: support@innovateuk.ukri.org<br>Tel: +44 300 3214357
**Sponsor [name and address]:**	University of Oxford, University Offices, Wellington Square, Oxford, OX1 2JD, UK<br>Email: research.services@admin.ox.ac.uk<br>Tel: +44 1865 270000

**Principal Investigator**	Prof. George Warimwe<br>KEMRI-Wellcome Trust Research Programme,<br>P.O. Box 230-80108<br>Kilifi, Kenya<br>Email: gwarimwe@kemri-wellcome.org<br>Tel: +254 709 983000
**Drug/Product Manufacturers**<br>**[name and address]:**	Advent S.r.l<br>Via Pontina Km 30, 600<br>00071<br>Pomezia (Roma) Italy<br>Email: bd@irbm.com<br>Tel: +39 06 91093692<br><br>Cobra Biologics Ltd<br>Science Park,<br>University of Keele,<br>Newcastle ST5 5SP, UK<br>Email: www.cobrabio.com/contact-us<br>Tel: +44 1782 714 181

Confidentiality Statement

The information contained herein is privileged or confidential and may not be disclosed unless such disclosure is required by applicable laws or regulations. In any event, persons to whom the information is disclosed must be informed that the information is privileged or confidential and may not be further disclosed by them. These restrictions on disclosure will apply equally to all future information supplied to you, which is indicated as privileged or confidential. This confidentiality statement also applies to data generated during the course of the study.

INVESTIGATOR'S APPROVAL OF THE PROTOCOL

A phase Ib/II single-blinded, randomised, controlled study to determine safety, immunogenicity and efficacy of the candidate Coronavirus Disease (COVID-19) vaccine ChAdOx1 nCoV-19 in adults in Kenya (COV004)

Protocol Number: KEMRI/SERU/CGMR-C/CSC197/4024

The undersigned acknowledge possession of and have read the Investigators' Brochure, version 11 dated 9th January 2021 and protocol "A phase Ib/II single-blinded, randomised, controlled study to determine safety, immunogenicity and efficacy of the candidate Coronavirus Disease (COVID-19) vaccine ChAdOx1 nCoV-19 in adults in Kenya" Version 2.1 dated 12th November 2021. Having fully considered all the information available, the undersigned consider that it is ethically justifiable to give ChAdOx1 nCoV-19 vaccine to selected participants according to the agreed protocol.

I understand that all information supplied to me in connection with this study is confidential information. This includes the Clinical Trial Protocol, Case Report Forms and any other preclinical and clinical data provided. I understand that no data are to be made public or published without prior knowledge and written approval by the University of Oxford.

By my signature below, I hereby attest that I have read, understood and agreed to abide by all the conditions, instructions and restrictions contained in Protocol "A phase Ib/II single-blinded, randomised, controlled study to determine safety, immunogenicity and efficacy of the candidate Coronavirus Disease (COVID-19) vaccine ChAdOx1 nCoV-19 in adults in Kenya" Version 2.1 dated 12th November 2021, and in accordance with the most recent Declaration of Helsinki and Good Clinical Practice and all applicable regulatory requirements. I acknowledge that the Sponsor of the study has the right to discontinue the study at any time.

12th November 2021

Principal Investigator Signature **Date**

Prof. George Warimwe (Principal Investigator)

Table of Contents

1. GENERAL INFORMATION 1
2. LAY SUMMARY 6
3. LIST OF INVESTIGATORS & COLLABORATORS 10
4. ABSTRACT 10
5. INTRODUCTION 12
5.1. Background Information 12
5.2. Name and description of the investigational product 14
5.3. Justification 23
6. TRIAL OBJECTIVES AND PURPOSE 25
7. TRIAL DESIGN 27
7.1. Overall Study Design and Plan Description 27
7.2. Study procedures (similar for phase Ib and phase II trial) 31
7.2.1. Recruitment 31
7.2.2. Informed consent/E-consent 32
7.2.3. Screening 33
7.2.4. Randomization/Enrolment 34
7.2.5. Scheduled visits 36
7.2.6. Unscheduled Visits 36
7.2.7. End of Treatment Visit 37
7.2.8. Study Restrictions 37
8. SELECTION AND WITHDRAWAL OF STUDY PARTICIPANTS 38
8.1. Description of the population to be studied and eligibility criteria 38
8.2. Inclusion criteria 38
8.3. Exclusion criteria 39
8.4. Managing withdrawals 40
8.5. Replacing withdrawn participants 41
9. TREATMENT OF STUDY PARTICIPANTS 42
9.1. ChAdOx1 nCoV-19 vaccine 42
9.2. Storage 42
9.3. Administration 42
9.4. Timing and selection of Doses 42
9.6. Packaging and Labelling 44
9.7. Control vaccine 45
9.8. Dispensing procedures 45
9.9. Dose Administration 45
9.10. Accountability 46
9.11. Unblinding 46
9.12. Prior/Concomitant Therapy 47
10. ASSESSMENT OF SAFETY 47
10.1. Adverse Events (AEs) 47
10.2. Definitions and monitoring of AEs 47
10.3. Documenting AEs 52
10.4. Reporting Serious Adverse Events (SAEs) and/or Unexpected AEs 52
10.5. Emergency Procedures 54
10.6. Pregnancy 54
10.7. Procedures for reporting any protocol violation(s) 54

11. LABORATORY INVESTIGATIONS 56
12. STATISTICS 57
12.1. Determination of sample size 58
12.2. Statistical and analytical plans 58
12.2.1. Data management 58
12.2.2. Data security, access and back up 58
12.2.3. Analysis of study endpoints 59
13. DIRECT ACCESS TO SOURCE DATA/DOCUMENTS 60
15. INTELLECTUAL PROPERTY 61
16. TIME FRAME/DURATION OF THE TRIAL 62
17. ETHICS 62
17.1. Human Subjects 62
17.2. Public, Community and Stakeholder Engagement 63
17.3. Compensation and Reimbursement 64
17.4. Patient Data Protection/Confidentiality 64
17.5. Data Sharing 64
17.6. Safety 65
18. ARCHIVING AND RECORD RETENTION 65
19. TRIAL MANAGEMENT 67
20. REPORTING, DISSEMINATION AND NOTIFICATION OF RESULTS 67
21. REFERENCES 68
22. APPENDICES 71
22.1. Work Plan 71
22.2. Roles of Investigators 72

GLOSSARY OF TERMS AND ABBREVIATIONS

**AE**	Adverse event
**CAST**	Community Advisory for Studies
**CBF**	Clinical Bio-Manufacturing Facility
**CLG**	Community Liaison Group
**COVID-19**	Coronavirus Disease 2019
**ChAdOx1 nCoV-19**	Chimpanzee Adenovirus Ox1 encoding the SARS-CoV-2 spike protein
**CGMRC**	Centre for Geographic Medicine Research Coast
**DSMB**	Data and Safety Monitoring Board
**ELISA**	Enzyme-linked immunosorbent assay
**ELISpot**	Enzyme-linked immunospot
**FBC**	Full Blood Count
**GCP**	Good Clinical Practice

**GDPR**	General Data Protection Regulation
**HbsAg**	Hepatitis B surface antigen
**HCV**	Hepatitis C Virus
**HCW**	Healthcare worker
**HIV**	Human Immunodeficiency Virus
**IC**	Informed Consent
**KCH**	Kilifi County Hospital
**KEMRI**	Kenya Medical Research Institute
**KWTRP**	KEMRI-Wellcome Trust research programme
**LSM**	Local safety monitor
**MERS**	Middle East Respiratory Syndrome
**NACOSTI**	National Commission for Science, Technology and Innovation
**OxTREC**	Oxford Tropical Research Ethics Committee
**PCR**	Polymerase Chain Reaction
**PI**	Principal Investigator
**PPB**	Pharmacy and Poisons Board
**PPE**	Personal Protective Equipment
**SAE**	Serious Adverse Event
**SARS**	Severe Acute Respiratory Syndrome
**SARS-CoV-2**	Severe Acute Respiratory Syndrome Coronavirus 2
**SOP**	Standard Operating Procedure
**SPC**	Summary of Product Characteristics
**SUSAR**	Suspected Unexpected Serious Adverse Reaction
**WHO**	World Health Organization

2. LAY SUMMARY

Formal Title: A phase Ib/II single-blinded, randomised, controlled study to determine safety, immunogenicity and efficacy of the candidate Coronavirus Disease (COVID-19) vaccine ChAdOx1 nCoV-19 in adults in Kenya (COV004)

Lay Title: A study to determine if a new vaccine safely generates good immune responses to protect adults in Kenya from Coronavirus Disease

What is the problem/background?

As of 11th February 2021, the coronavirus disease (COVID-19) pandemic has affected over 106 million people globally, with over 2 million deaths. The disease has been reported in over 200

countries, including Kenya where over 102,000 cases have been reported. Several vaccines have been approved for emergency use against COVID-19 in different regions. As the number of cases continue to rise globally, use of effective vaccines remains the best way to control the pandemic and to safely relax physical distancing and other restrictions. There are over 170 COVID-19 vaccine candidates in development, with 63 of these currently undergoing evaluation in human clinical trials (as of 9th February 2021). Of the 63 vaccines, ChAdOx1 nCoV-19, a candidate vaccine developed by our longstanding collaborators at the University of Oxford in partnership with AstraZeneca, is among the most advanced and has received Emergency Use Authorisation from various national and international regulators, including the World Health Organization (WHO). ChAdOx1 nCoV-19 has shown promise in a range of animal models of COVID-19 disease and has already been administered to over 10,000 adult volunteers in the United Kingdom, Brazil and South Africa (Registration numbers: NCT04324606, NCT04400838, ISRCTN89951424 and NCT04444674). The WHO has recommended that two standard doses of ChAdOx1 nCoV-19 be administered at an 8- to 12-week interval in people aged 18 years and older. This dosing regimen was shown in the above clinical trials to be safe and effective in preventing symptomatic COVID-19, with no severe cases and no hospitalisations from COVID-19 more than 14 days after the second dose.

What questions are we trying to answer?

Vaccine performance from studies in other populations may not be generalizable to Kenya. For instance, differences have been observed in malaria vaccine and ebola vaccine immune responses and reactions in European vs African populations. We plan to evaluate whether the ChAdOx1 nCoV-19 vaccine is safe, elicits good immune responses and protects Kenyan adults from COVID-19. The persistence of these immune responses will be investigated up to 12 months following immunization with a single dose of vaccine.

Where is the study taking place, how many people does it involve and how are they selected?

This study will be conducted among adults aged ≥18 years and will take place in the coastal counties of Kilifi and Mombasa, where high numbers of COVID-19 cases have been detected. The study will enrol frontline workers in key service areas (as per the government of Kenya definition) including healthcare workers and allied healthcare professionals, emergency medical personnel, Kenya Ports Authority staff, security staff, laboratory scientists working on COVID-19 response, the Kenya Police and security officers to name a few. These have been prioritised due to their high

risk of occupational exposure to COVID-19, hence their urgent need for protection. However, due to the risk of infection from ongoing community SARS-CoV-2 transmission in Kenya, other members of public will also be eligible for the study. We will enrol a total of 400 participants; 40 in phase 1b, and 360 in phase II.

What does the study involve for those who are in it?
Before a face-to-face visit, potential participants will undergo pre-screening**.** We will conduct an abbreviated eligibility check for potential volunteers so that those who do not meet the threshold for potential eligibility don't attend clinic to avoid congestion. Prior to enrolment, participants will complete screening procedures to ensure they are in good health after they give written informed consent. This will include blood sampling for a series of tests to ensure eligibility, and collection of nose and throat swabs to screen for SARS-CoV-2 infection. Safety and immunogenicity data accrued so far from the UK trials support the use of two doses of ChAdOx1 nCoV-19 vaccine, administered at least one to 3 months apart. A longer interval between doses results in better immune responses and higher efficacy and this has informed WHO's recommendation that two standard doses of ChAdOx1 nCoV-19 be administered at an 8- to 12-week interval. Individuals who are healthy and free of COVID-19 will be randomised for vaccination with either two doses of ChAdOx1 nCoV-19 (n=200; 20 in phase Ib, 180 in phase II) or two doses of a licensed rabies vaccine (n=200; 20 in phase Ib, 180 in phase II) used as a 'control' for comparison. The time interval between the two doses will be 3 months for both phase Ib and phase II. Participants in both phase Ib and phase II will be followed up for 12 months from the first dose of vaccine, during which data on any vaccine-associated side effects will be collected and reviewed by an independent Data Safety and Monitoring Board (DSMB). These safety assessments will be performed throughout the study. Blood, nose and throat swabs will be collected during screening and scheduled follow up visits (10 visits over the duration of the study) for monitoring of immune responses and acquisition of COVID-19. Participants will be asked to check their temperature before visits. Participants will be directed to continue to adhere to the current COVID-19 preventive measures (including physical distancing, regular hand washing and cough/sneeze etiquette, as well as the infection control procedures in their workplace) even after vaccination since there is no evidence yet on the efficacy of the vaccine. In the event that any of the study participants present with symptoms consistent with COVID-19 during the study, we will immediately notify public health authorities to facilitate their sampling, clinical management and quarantine as per the

prevailing national guidelines. Participants will remain in follow up and their clinical outcome documented until the day 365 clinic visit when the scheduled final clinical assessment and sampling will be done.

What are the benefits and risks/costs of the study for those involved?

Participants will benefit by knowledge of their general health status, including whether or not they have COVID-19. Participants will also have close oversight and treatment support from the study team although this will primarily be for risk monitoring. ChAdOx1 nCoV-19 vaccine has been well tolerated with no safety concerns in the over 10,000 adult volunteers that have been vaccinated in the UK, Brazil and South Africa (Registration numbers: NCT04324606, NCT04400838, ISRCTN89951424 and NCT04444674). The vaccine has received Emergency Use Authorisation from various national and international regulators, including the WHO. The potential risk to participants in Kenya is considered to be low and these mainly relate to phlebotomy and vaccination. During vaccination and when collecting blood samples, the participants may experience some discomfort on the injection site, which gets better after a short while. Participants may also have an ongoing risk if their participation becomes public knowledge and the stigma associated with the disease is directed towards them. All participants will also be offered the opportunity to receive a complete course of the rabies vaccine at the end of the study. The control group will be offered the ChAdOx1 nCoV-19 vaccine at the end of the trial. If the vaccine is licensed at this point, then this will be done as a non-research procedure (or after a short delay if licensing is under consideration). If the vaccine is still not licensed, then the investigators will write to SERU, OxTREC and PPB to ask their input on the appropriateness of vaccination of the control group based on all available global data on safety and efficacy. Use of the unlicensed vaccine will require a protocol amendment in order to allow vaccination to take place as a research procedure. The required monitoring, follow up and safety assessments after vaccination will depend on the data available from the trial and from other related trials (e.g. UK, Brazil and South Africa), and will be specified in full in the amendment.

How will the study benefit society?

COVID-19 is currently a global pandemic with the number of cases and deaths continuing to rise on a daily basis. If found to be safe and good in generating protective immune responses, the ChAdOx1 nCoV-19 will be further developed for use in controlling COVID-19.

When does the study start and finish?

The study will start on receipt of ethical and regulatory approvals. Participants will be followed up for 1 year following vaccination, and we anticipate the total study duration including community engagement activities and post-study reporting to take approximately 2 years.

3. LIST OF INVESTIGATORS & COLLABORATORS

Institutions	Investigators
**KEMRI CGMRC**	George Warimwe (PI), Samuel Sang, Henry Karanja, John Gitonga, Philip Bejon, Caroline Ngetsa, Charles Agoti, Stanley Cheruiyot, Amek Nyaguara, Mainga Hamaluba, Benedict Orindi, Marianne Munene, Neema Mturi, Isabella Ochola-Oyier, Noni Mumba, Eunice Nduati
**Ministry of Health**	Nadia Aliyan, Kadondi Kasera
**University of Oxford**	Adrian Hill, Sarah Gilbert, Andrew Pollard, Teresa Lambe, Alexander Douglas
	**Collaborators**
**Ministry of Health**	Rashid Aman, Irene Njau

4. ABSTRACT

As of 11th February 2020, the coronavirus disease (COVID-19) pandemic has affected over 106 million people globally, with over 2 million deaths. The disease has been reported in over 200 countries, including Kenya where over 102,000 cases have been reported. Some of the vaccines approved for emergency use in different regions for use against COVID-19 include Pfizer/BioNTech, Moderna, Sinovac, and ChAdOx1 nCoV-19 among others. As the number of cases continue to rise globally, use of effective vaccines remains the best way to control the pandemic and to safely relax physical distancing and other restrictions. There are over 170 COVID-19 vaccine candidates in development, with 63 of these currently undergoing evaluation in human clinical trials (as of 9th February 2021). Of the 63 vaccines, ChAdOx1 nCoV-19, a candidate vaccinedeveloped by our longstanding collaborators at the University of Oxford in partnership with

AstraZeneca, is among the most advanced and has received Emergency Use Authorisation from various national and international regulators, including the World Health Organization (WHO). ChAdOx1 nCoV-19 has shown promise in a range of animal models of COVID-19 disease and has already been administered to over 10,000 adult volunteers in the United Kingdom, Brazil and South Africa (Registration numbers: NCT04324606, NCT04400838, ISRCTN89951424 and NCT04444674). The vaccine has been found to be safe and to provide protection against COVID-19 in these studies.

In this single-blinded randomized controlled phase Ib/II study we plan to evaluate the safety and immunogenicity of ChAdOx1 nCoV-19 ($5x10^{10}$ vp) as compared to rabies vaccine among 400 adults aged ≥18 years. The study will take place in the coastal counties of Kilifi and Mombasa, where high numbers of COVID-19 cases have been detected. The study will enrol frontline staff such as; healthcare workers, allied health professionals, truckers, security personnel, banking personnel, supermarket staff, police, security personnel, prison workers, laboratory technicians, scientists, logistics personnel, public transport workers including aviation industry amongst others. These populations have been prioritised due to their high risk of occupational exposure to COVID-19, hence their urgent need for protection. However, due to the risk of infection from ongoing community SARS-CoV-2 transmission in Kenya, other members of public will also be eligible for the study. Participants will be randomized to receive two doses of either ChAdOx1 nCoV-19 (n=200) or rabies vaccine (n=200). Out of these, 40 will be enrolled in the phase Ib trial, and the remainder to the phase II trial (180 per vaccine). The time interval between the two doses will be 3 months for both phase Ib and phase II. After each vaccination, participants will be required to complete diary cards (paper or electronic) and record any solicited and unsolicited adverse events experienced for 6 days following vaccination while at home. Each participant will have 10 clinic visits in the course of the study and some of the activities that will be carried out will include; physical assessment, checking vital signs, phlebotomy and safety assessment. If found to be safe and effective in generating protective immune responses, the ChAdOx1 nCoV-19 will be further developed for use in controlling COVID-19, including in populations in Kenya and other countries in Africa.

5. INTRODUCTION

5.1. Background Information

The emergence of the SARS-CoV-2 has posed a significant public health crisis throughout the world. SARS-CoV-2 was first detected in Wuhan, China in December 2019 and its initial transmission to human linked, to the local wet markets (1). Later, human-to-human transmission was reported resulting in increased spread throughout Wuhan and other areas within the Hubei Province. The number of cases continued to increase significantly and spread across China and other countries in the Asian region (2). Initially, the Chinese government referred to the illness as the novel coronavirus pneumonia. Later, the WHO, following an emergency meeting on 30th January 2019, declared the outbreak as a global public health emergency, and on February 11, the virus was renamed as SARS-CoV-2 and the resulting disease as COVID-19 (Coronavirus Disease 2019). As of 11th February 2020, there were over 106 million COVID-19 cases globally and over 2 million deaths in over 200 countries, of which over 2.6 million cases and over 65,000 deaths were in Africa (https://www.who.int/emergencies/diseases/novel-coronavirus-2019/situation-reports). Most health systems are characterized by an increasing number of confirmed as well as suspected cases, limited personal protection equipment (PPE), lack of drugs, and overwhelming workload (3).

COVID-19 is mainly transmitted through the inhalation of droplets from an infected person and the clinical features are reported to range from asymptomatic manifestations to acute respiratory distress (4) and death. In more severe cases of the illness, multi organ dysfunction is reported. Touching of contaminated objects or surfaces has also been reported to cause infections although this is not thought to be the primary mode of transmission (5). The incubation period ranges from 2 to 14 days (mean of 5.8 days) and there are variations in the symptoms reported. In addition, SARS-CoV-2 is reported to have a reproductive number (R_0) of 2.2, which means that a single infected person has a likelihood of transmitting the infection to about 2 susceptible individuals (6). The common symptoms of COVID-19 include breathlessness, cough, fever, fatigue, malaise and sore throat (7). These symptoms are also characteristics of many other respiratory illness. In individuals with other comorbidities such as heart conditions, diabetes and hypertension and among patients over 60 years, current data shows significant mortality and severe respiratory disease (1). While a significant number of the population is reported to be asymptomatic, COVID-19 has a case fatality rate of approximately 2 to 3 percent, but these rates are variable based on the geographic location

and context. An effective vaccine is urgently needed to provide protection against SARS-CoV-2 infection and the associated clinical disease.

Current preventive measures and global public health response to COVID-19 are primarily based on interventions that were implemented for other flu epidemics in the past. Such measures include avoiding exposure through quarantine and social distancing (8). Additionally, isolation and management of cases, tracing of contacts, infection control within the healthcare system, and community containment as well as strict quarantine period for travellers have been implemented. There have been challenges in active identification and isolation of cases in many resource limited settings (6). Proactive case identification in the community and hospital settings would allow early clinical management of cases and result in better disease prognosis among the high-risk groups. Moreover, such an approach is likely to provide evidence on the manifestation of subclinical infections allowing better understanding of COVID-19 severity as well as limit the spread of the illness. In case of infection, measures such as covering of sneezes and coughs, regular hand washing, use of face masks, and isolation have been proposed to reduce the risk of exposure (9). Vaccination still remains the most effective intervention for SARS-CoV-2 infection in the event that the existing preventive measures do not eliminate it from the population (2).

While there have been considerable efforts in the past to develop therapies against other human coronavirus infections in the past decades such as SARS (Severe Acute Respiratory Syndrome) and MERS (Middle East Respiratory Syndrome), there are no licensed antiviral agents or vaccines to date (10). Due to the lack of effective therapies against COVID-19, the current treatment strategies target symptomatic and respiratory support. The WHO treatment guidelines recommend supportive care such as fluid and oxygen therapy, and antibiotics for the treatment of secondary bacterial infection coupled with isolation of patients with severe forms of the disease (7). Some of the therapeutic drugs that have been considered for the management of the illness include lopinavir/ritonavir, which are HIV protease inhibitors, dexamethasone, tocilizumab and remdesivir, a broad-spectrum antiviral agent (8). Some of these drugs have been reported in MERS-CoV animal models and shown promising results. Their use alone or in combination with interferon-β is also being investigated (7). In other settings, options such as monoclonal antibodies treatments and convalescent plasma are being considered. Nonetheless, prior to the utilization of such drugs for COVID-19 patients with pneumonia, safety and efficacy studies are required.

5.2. Name and description of the investigational product

ChAdOx1 nCoV-19 vaccine consists of the replication-deficient simian adenovirus vector ChAdOx1, containing the structural surface glycoprotein (Spike protein) antigen of the SARS CoV-2 (nCoV-19), with a leading tissue plasminogen activator (tPA) signal sequence. The spike protein is a type I, trimeric, transmembrane glycoprotein located at the surface of the viral envelope of coronaviruses, which can be divided into two functional subunits: The N-terminal S1 and the C-terminal S2. S1 and S2 are responsible for cellular receptor binding via the receptor binding domain (RBD) and fusion of virus and cell membranes respectively, thereby mediating the entry of SARS-CoV-2 into target cells (Figure 1)(11). The roles of the spike protein in receptor binding and membrane fusion make it an ideal target for vaccine and antiviral development, as it is the main target for neutralising antibodies. ChAdOx1 nCoV-19 expresses a codon-optimised coding sequence for the Spike protein from genome sequence accession GenBank: MN908947. The tPA leader sequence has been shown to be beneficial in enhancing immunogenicity of another ChAdOx1 vectored coronavirus vaccine (ChAdOx1 MERS)(12). Following vaccination, the Spike glycoprotein primes the host immune system to attack the coronavirus in case of any infections (Figure 1).

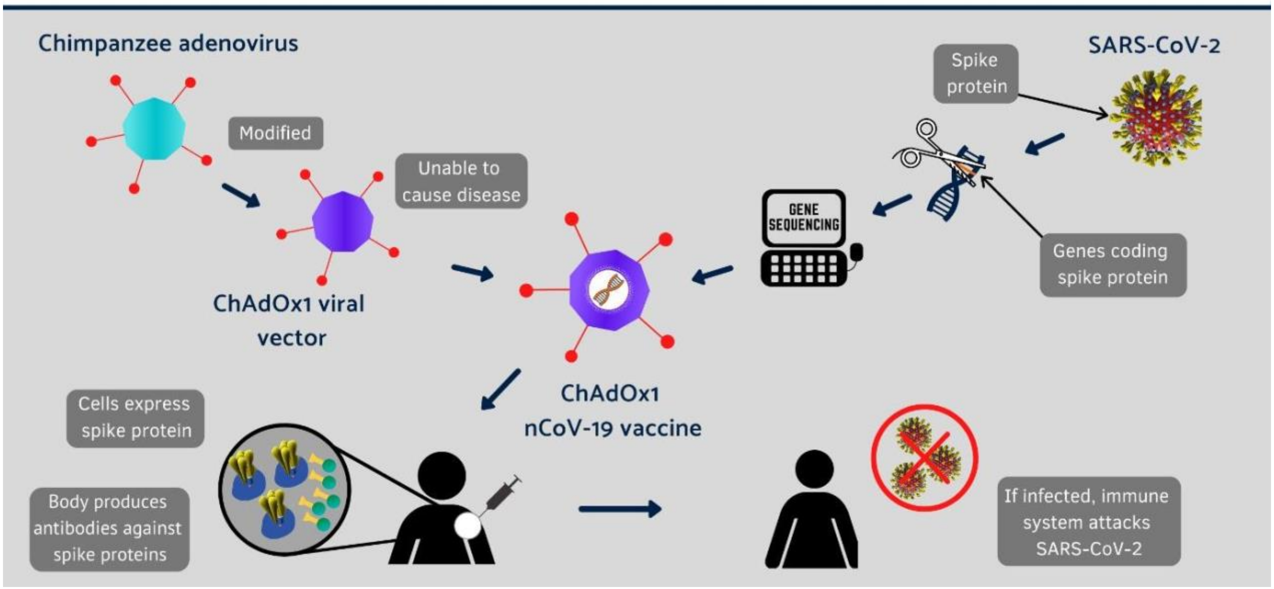

Figure 1*: Illustration of the ChAdOx1 nCoV-19 vaccine development. Following vaccination, the Spike glycoprotein primes the host immune system to generate responses that will attack the coronavirus in case of any infections*

5.2.1. Pre-clinical studies

5.2.1.1. Immunogenicity (Jenner Institute, unpublished)

Mice (BALB/c and CD-1) were immunised with ChAdOx1 expressing SARS-CoV-2 Spike protein or green fluorescent protein (GFP). Spleens were harvested for assessment of gamma interferon (IFN-γ) ELISpot responses and serum samples were taken for assessments of antibody responses to the Spike protein domains (S1 and S2) on ELISA at 9- or 10-days post-vaccination. The results of this study show that a single dose of ChAdOx1 nCoV-19 was immunogenic in mice (Figures 2 and 3).

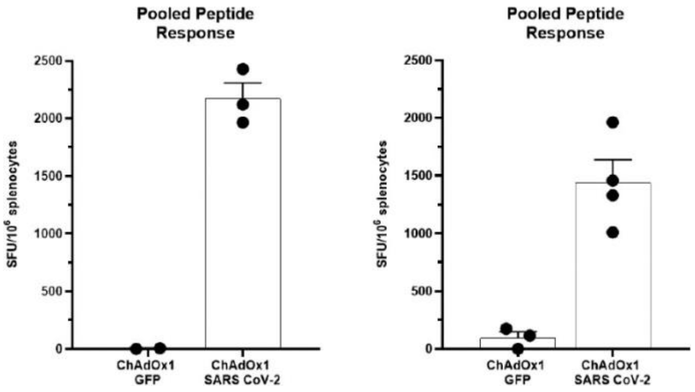

Figure 2. *Summed splenic IFN-γ ELISpot responses of BALB/c (left panel) and CD-1 (right panel) mice, in response to peptides spanning the spike protein from SARS-CoV-2, nine or ten days post vaccination, with 1.7 × 10^{10} vp ChAdOx1 nCoV-19 (pre-clinical product termed ChAdOx1 SARS CoV-2) or 8 × 10^{9} vp ChAdOx1 GFP. Mean with standard errors (SEM) are depicted.*

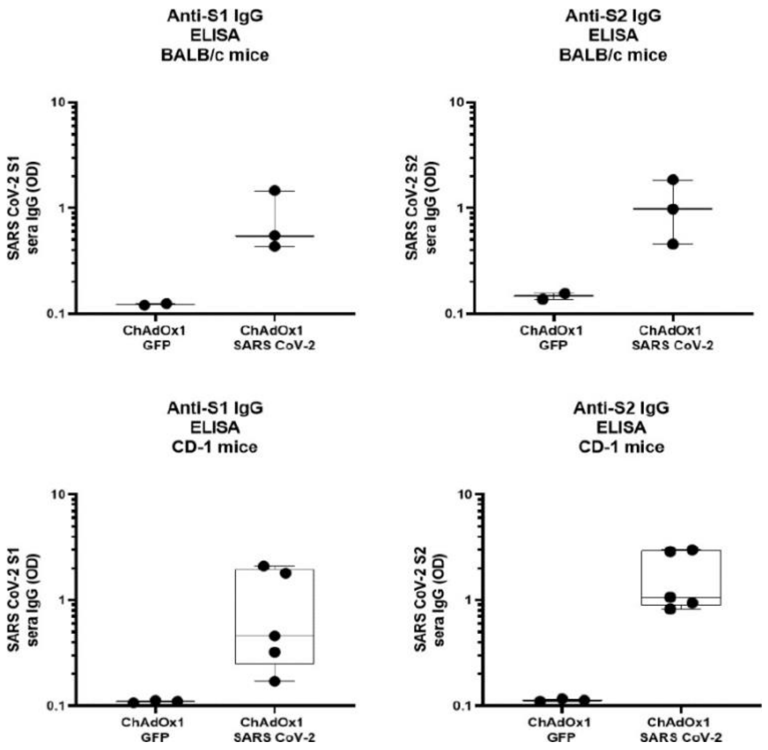

Figure 3*. Box and whisker plot of the optical densities following ELISA analysis of BALB/C mouse sera (Top panel) incubated with purified protein spanning the S1 domain (left) or purified protein spanning the S2 domain (right) of the SARS-CoV-2 spike nine or ten days post vaccination, with 1.7×10^{10} vp ChAdOx1 nCoV-19 or 8×10^{9} vp ChAdOx1 GFP. Box and whisker plots of the optical densities following ELISA analysis of CD-1 mouse sera (Bottom panel) incubated with purified protein spanning the S1 domain (left) or purified protein spanning the S2 domain (right) of the SARS-CoV-2 spike.*

In another study, two mouse strains (BALB/c, N=5 and outbred CD1, N=8) were vaccinated intramuscularly (IM) with ChAdOx1 nCoV-19 or ChAdOx1 GFP, a control vaccine expressing green fluorescent protein. Humoral and cellular immunity were studied 9-14 days later (13) . Total IgG titres were detected against spike protein subunits S1 and S2 in all vaccinated mice (13); also see Fig. 5 & 6 in Investigator's Brochure), further confirming the immunogenicity of ChAdOx1 nCoV-19. The immunogenicity of ChAdOx1 nCoV-19 has now also been confirmed in a pig model, where high levels of neutralizing antibody and T-cells were observed (see Investigator's Brochure).

5.2.1.2. Efficacy

Pre-clinical efficacy of ChAdOx1 nCoV-19 was determined in two independent studies in non-human primates (rhesus macaques). The vaccine was found to be safe, highly immunogenic and significantly reduced viral load in bronchoalveolar lavage fluid and respiratory tract tissue of vaccinated animals challenged with SARS-CoV-2 compared with control animals (13). No pneumonia was observed in ChAdOx1 nCoV-19 vaccinated monkeys. Importantly, no evidence of immune-enhanced disease following viral challenge in vaccinated animals was observed. Full details of these studies can be found in the Investigator’s Brochure). In a further efficacy study in ferrets, ChAdOx1 nCoV-19 was safe, immunogenic and conferred protection in vaccinated ferrets challenged with live SARS-CoV-2 virus (see Investigator’s Brochure).

5.2.1.3. Antibody dependent enhancement

Safety concerns around the use of full length coronavirus Spike glycoproteins and other viral antigens (nucleoprotein) as a vaccine antigen have been raised following historical and limited reports of immunopathology and antibody dependant enhancement (ADE) reported in vitro and post SARS-CoV challenge in mice, ferrets and non-human primates immunised with whole SARS-CoV inactivated or full-length S protein based vaccines, including a study using Modified Vaccinia Ankara as a vector (14–16). To date, there has been one report of lung immunopathology following MERS-CoV challenge in mice immunised with an inactivated MERS-CoV candidate vaccine (17). However, in preclinical studies of ChAdOx1 immunisation and MERS-CoV challenge, no ADE was observed in hDPP4 transgenic mice, dromedary camels or non-human primates (18–20).

The risks of inducing lung immunopathology in the event of COVID-19 disease following ChAdOx1 nCoV-19 vaccination are unknown. In the non-human primate (13) and ferret challenge studies presented in the Investigator’s Brochure no evidence of lung pathology was observed. Safety assessment of the vaccine will be conducted throughout the study in Kenya, and is ongoing in the UK, Brazil and South Africa (Registration numbers: NCT04324606, NCT04400838, ISRCTN89951424 and NCT04444674). Results will be reviewed on an ongoing basis as they emerge and will inform discussions on risk/benefit to participants receiving the Investigational Product. All pathology data arising from animal challenge studies of other SARS-CoV-2 vaccine candidates will also be taken into account. These data will also be considered in the DSMB safety reviews. The DSMB, will have sight of all safety data from the trial sites.

5.2.2. Previous clinical experience

ChAdOx1 vectored vaccines expressing different inserts have previously been used in over 320 healthy volunteers taking part in clinical trials conducted by or in partnership with the University of Oxford in the UK, Switzerland, Saudi Arabia and Uganda (Tables 1 and 2). A similar vector, ChAd63, has been given to 74 volunteers in Kilifi, Kenya across two studies with few adverse events reported (21,22). Most importantly, a ChAdOx1 vectored vaccine expressing the full-length Spike protein from another Betacoronavirus, MERS-CoV, has been given to 31 participants to date as part of MERS001 and MERS002 trials. ChAdOx1 MERS was given at doses ranging from $5x10^9$ vp to $5x10^{10}$ vp with no serious adverse reactions reported (23). Further safety and immunogenicity results on ChAdOx1 MERS can be found on the Investigator’s Brochure for ChAdOx1 nCoV-19 for reference.

Clinical trials of ChAdOx1 vectored vaccines encoding antigens for Influenza (fusion protein NP+M1), Tuberculosis (85A), Prostate Cancer (5T4), Malaria (LS2), Chikungunya (structural polyprotein), Zika (prM and E), MERS-CoV (full-length Spike protein) and Meningitis B are listed below. None of the below mentioned clinical trials reported serious adverse events associated with the administration of ChAdOx1, which was shown to have a good safety profile.

Table 1: Clinical experience with ChAdOx1 viral vector vaccines

Country	Trial	Vaccine	Age	Route	Dose	Number of Volunteers (Received ChAdOx1)	Publication / Registration Number
Kenya	ECCT/11/12/02	ChAd63-MVA ME-TRAP	18-50	IM	5×10^{10} vp	30	(21)
UK	FLU004	ChAdOx1 NP+M1	18-50	IM	5×10^{8} vp	3	Antrobus et al, 2014. Molecular Therapy. DOI:10.1038/mt.2013.284 (24)
					5×10^{9} vp	3	
					2.5×10^{10} vp	3	
					5×10^{10} vp	6	
UK	FLU005	ChAdOx1 NP+M1	18-50	IM	2.5×10^{10} vp	12	Coughlan et al, 2018. EBioMedicine DOI: 10.1016/j.ebiom.2018.02.011 DOI: 10.1016/j.ebiom.2018.05.001 (25,26)
		MVA NP+M1 (week 8)	18-50	IM	2.5×10^{10} vp	12	
		ChAdOx1 NP+M1	18-50	IM	2.5×10^{10} vp	12	
		MVA NP+M1 (week 52)	18-50	IM	2.5×10^{10} vp	9	
		MVA NP+M1	>50	IM	2.5×10^{10} vp	12	
		ChAdOx1 NP+M1 (week 8)	>50	IM	2.5×10^{10} vp	12	
UK	TB034	ChAdOx1 85A	18-50	IM	5×10^{9} vp	6	Wilkie et al, 2020 Vaccine DOI: 10.1016/j.vaccine.2019.10.102 (27)
					2.5×10^{10} vp	12	
		ChAdOx1 85A MVA85A (week 8)	18-50	IM	2.5×10^{10} vp	12	
		ChAdOx1 85A (x2, 4weeks apart) MVA85A (at 4 months)	18-50	IM	2.5×10^{10} vp	12	
Switzerland	TB039 (ongoing)	ChAdOx1 85A	18-55	Aerosol	1×10^{9} vp	3	Clinicaltrials.gov: NCT04121494
				Aerosol	5×10^{9} vp	3	

				Aerosol	1 x 10^{10} vp	11	
				Aerosol/ IM	1 x 10^{10} vp	15	
Uganda	TB042 (ongoing)	ChAdOx1 85A	18-49	IM	5 x 10^{9} vp	6	Clinicaltrials.gov: NCT03681860
					2.5 x 10^{10}	6	
UK	VANCE01	ChAdOx1.5T4 MVA.5T4	18-75	IM	2.5 x 10^{10} vp	34	Clinicaltrials.gov: NCT02390063
UK	ADVANCE (ongoing)	ChAdOx1.5T4 MVA.5T4	≥18	IM	2.5 x 10^{10} vp	23 (as of Feb 20)	Clinicaltrials.gov: NCT03815942
UK	VAC067	ChAdOx1 LS2	18-45	IM	5 x 10^{9} vp	3	Clinicaltrials.gov: NCT03203421
					2.5 x 10^{10} vp	10	
UK	VAMBOX	ChAdOx1 MenB.1	18-50	IM	2.5 x 10^{10} vp	3	ISRCTN46336916
					5 x 10^{10} vp	26	
UK	CHIK001	ChAdOx1 Chik	18-50	IM	5 x 10^{9} vp	6	Clinicaltrials.gov: NCT03590392 DOI: https://doi.org/10.4269/ajtmh.abstract2019 Abstract #59, page 19.
					2.5 x 10^{10} vp	9	
					5 x 10^{10} vp	9	
UK	ZIKA001 (ongoing)	ChAdOx1 Zika	18-50	IM	5 x 10^{9} vp	6	Clinicaltrials.gov: NCT04015648
					2.5 x 10^{10} vp	3 (as of Feb 20)	
					5 x 10^{10} vp	-	
UK	MERS001 (ongoing)	ChAdOx1 MERS	18-50	IM	5 x 10^{9} vp	6	Clinicaltrials.gov: NCT03399578 DOI:https://doi.org/10.1016/S1473-3099(20)30160-2
					2.5 x 10^{10} vp	9	
					5 x 10^{10} vp	9	
					2.5 x 10^{10} vp (homologous prime-boost)	3	
Saudi Arabia	MERS002 (ongoing)	ChAdOx1 MERS	18-50	IM	5 x 10^{9} vp	4	Clinicaltrials.gov: NCT04170829
					2.5 x 10^{10} vp	3	
					5 x 10^{10} vp	-	
UK	HBV001 (ongoing)	ChAdOx1-HBV	18-65	IM	2.5 x 10^{10} vp	5	Clinicaltrials.gov: NCT04297917

5.2.3. Clinical experience with ChAdOx1 nCoV-19

ChAdOx1 nCoV-19 has already been administered to over 10,000 adult volunteers in the United Kingdom, Brazil and South Africa (Registration numbers: NCT04324606, NCT04400838, ISRCTN89951424 and NCT04444674) as detailed in Table 2 below. There have been no serious adverse reactions (SARs) or suspected unexpected serious adverse reactions (SUSARs) definitively

associated with ChAdOx1 nCoV-19 to date. In the ongoing ChAdOx1 nCoV-19 trials, safety reviews have been undertaken when trial volunteers developed unexplained symptoms of the nervous system including reduced sensitivity to touch or limb weakness that led to a study pause across sites while a safety review took place. After independent review, these illnesses were either considered unlikely to be associated with the vaccine or there was insufficient evidence to say for certain that the illnesses were or were not related to the vaccine. In each of these cases, after considering the information, the independent reviewers recommended that vaccinations should continue. Close monitoring of the affected individuals and other participants will be continued.

Table 2: Summary of ongoing ChAdOx1 nCoV-19 vaccine trials globally

Country	Trial	Vaccine	Age	Route	Dose	Number of Volunteers (Received ChAdOx1)	Publication / Registration Number
UK	COV001 (ongoing)	ChAdOx1 nCoV-19	18-55	IM	5×10^{10} vp	533	Clinicaltrials.gov: NCT04324606 Folegatti, Ewer et al. Lancet 2020 (28)
					5×10^{10} vp (homologous prime-boost, 4 weeks interval)	10	
					5×10^{10} vp (homologous prime-boost, 8 weeks interval)	20	
					5×10^{10} vp prime and 2.5×10^{10} vp boost (8 weeks interval)	32	
UK	COV002 (ongoing)	ChAdOx1 nCoV-19	18-55	IM	2.2×10^{10} vp	1765	Clinicaltrials.gov: NCT04400838
			18-55		2.2×10^{10} vp (homologous prime-boost)	50	
			56-69		2.2×10^{10} vp	30	
			56-69		2.2×10^{10} vp (homologous prime-boost)	30	
			≥70		2.2×10^{10} vp	50	
			≥70		2.2×10^{10} vp (homologous prime-boost)	50	
			5-12		2.5×10^{10} vp	-	
			18-55		5×10^{10} vp	2267	
			56-69		5×10^{10} vp	30	
			56-69		5×10^{10} vp (homologous prime-boost)	30	

			≥70		5 x 10^{10} vp	50	
			≥70		5 x 10^{10} vp (homologous prime-boost)	50	
Brazil	COV003	ChAdOx1 nCoV-19	18-55	IM	5 x 10^{10} vp (homologous prime-boost)	5,000 as of 26th Oct 2020	ISRCTN89951424
South Africa	COV005	ChAdOx1 nCoV-19	18-55	IM	5 x 10^{10} vp (homologous prime-boost)	950 as of 26th Oct 2020	Clinicaltrials.gov: NCT04444674

5.2.4. ChAdOx1 nCoV-19 safety and immunogenicity data from the UK Phase I/II trial

The preliminary safety data on volunteers receiving ChAdOx1 nCoV-19 in the UK phase I/II trial are promising. The first participant was randomised on April 23, 2020. In total 1077 participants were randomised 1:1 to ChAdOx1 nCoV-19 (n=543) or MenACWY (n=534) control vaccine, 10 of whom were enrolled in a non-randomised group to evaluate a two-dose ChAdOx1 nCoV-19 vaccination schedule. Detailed safety and immunogenicity data are presented in the Investigator’s Brochure and in a recent publication (28). There have been no serious adverse reactions (SARs) or suspected unexpected serious adverse reactions (SUSARs) definitively associated with ChAdOx1 nCoV-19 to date. The majority of adverse events (AEs) reported were self-limiting and mild or moderate in severity, but severe events were also reported with their onset within the first 72h (most frequently within the first 24h). The vaccine was well tolerated despite the reactogenicity profile, with no safety concerns. The vast majority of solicited local and systemic AEs were short-lived and resolved within 1-7 days. Furthermore, a single dose of ChAdOx1 nCoV-19 was able to elicit both humoral and cellular responses (see below and Investigator’s Brochure) and a booster dose enhanced these responses (28).

ChAdOx1 nCoV-19 immunogenicity in adult volunteers in the UK phase I/II trial

Antibody responses measured by live SARS-CoV-2 neutralisation assays

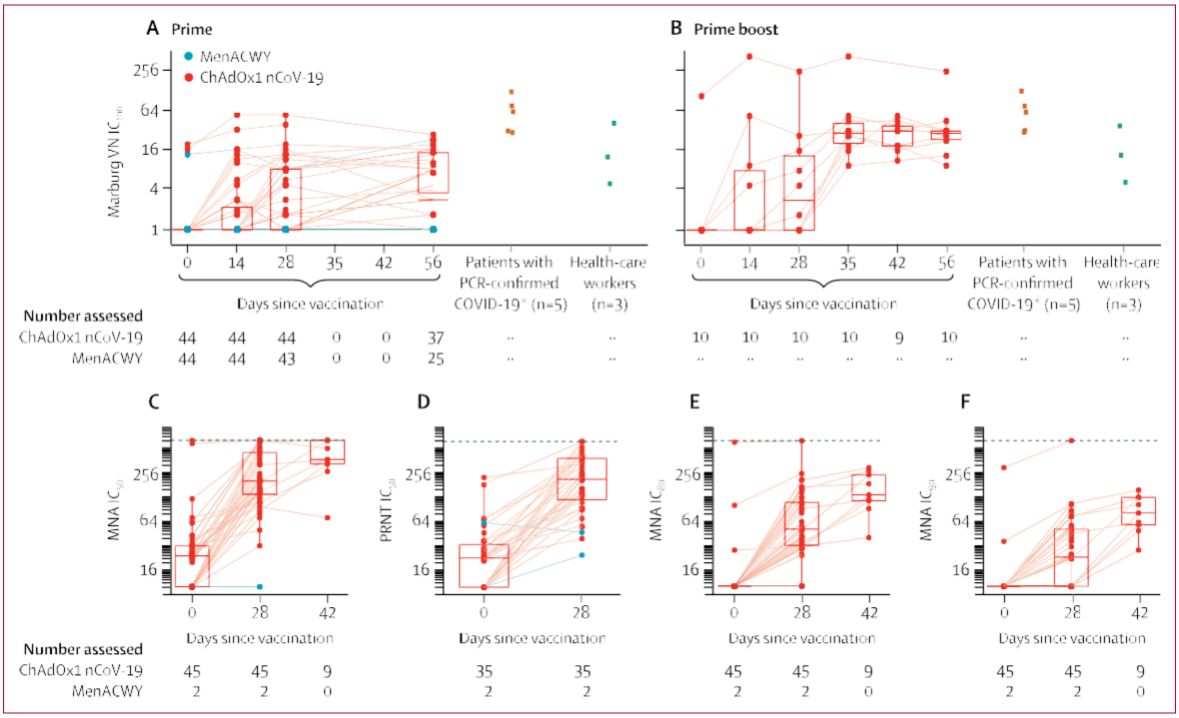

Panels A and B show live SARS-CoV-2 neutralisation (Marburg VN) in prime (A) and prime boost (B) trial participants (boosted at day 28) and convalescent plasma from patients with PCR-confirmed COVID-19 and asymptomatic health-care workers. Panels C, E, and F show the PHE MNA (at IC_{50}, IC_{80}, and IC_{90}, respectively) and panel D the PHE $PRNT_{50}$. The day 42 timepoint was only measured in participants who received a booster dose at day 28. Solid lines connect samples from the same participant. Boxes show median (IQR). Dotted lines show upper limits of detection. MenACWY=meningococcal group A, C, W-135, and Y conjugate vaccine. PHE=Public Health England. MNA=microneutralisation assay. PRNT=plaque reduction neutralisation test. VN=virus neutralisation. IC=inhibitory concentration.

Interferon-γ ELISpot responses to peptides spanning the SARS-CoV-2 spike insert

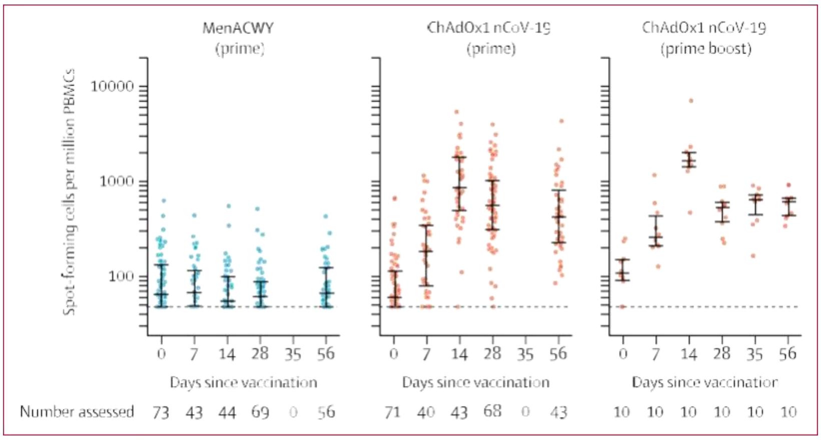

Error bars show median (IQR). The lower limit of detection, indicated with the dotted line, is 48 spot-forming cells per million PBMCs. PBMC=peripheral blood mononuclear cell. SARS-CoV-2=severe acute respiratory syndrome coronavirus 2. ELISpot=enzyme linked immunospot. MenACWY=meningococcal group A, C, W-135, and Y conjugate vaccine.

5.3. Justification

The COVID-19 pandemic has had global overwhelming effect on human life and the healthcare system (6). It is a recent example of the global emerging complex threats of infectious diseases. Along with other challenges such as antimicrobial resistance, emerging infections are challenging and likely to result in economic losses, global fears and significant negative implications on mobility and growth of industries (29). As such, intensive efforts to develop effective therapies and vaccines against COVID-19 is essential (6). In case effective therapies are not developed soon, there is a danger that the illness will have catastrophic impacts especially in countries that have fragile economies, health systems and limited resources. Further, due to the high infectivity of the SARS-CoV-2 virus, this poses a significant risk to health workers and would eventually crumble healthcare operations worldwide.

While life-threatening illness is reported to affect older individuals and people with pre-existing conditions, current evidence also shows that COVID-19 can cause both symptomatic and asymptomatic infections in a wider age-group although the frequency is not yet known (8). As a result, many asymptomatic cases are likely to be missed in the statistics being reported and continue to pose a threat to the containment of the pandemic. Furthermore, the role of children in the transmission of the infection remains unknown but the prevalence of COVID-19 in this age-group is reported to be low. The current gaps relating to the COVID-19 pandemic coupled with the unavailability of licensed antiviral therapies support the need for an effective vaccine to contain its spread.

Safety, immunogenicity and efficacy data from the ongoing trials in the UK, Brazil and South Africa have supported the emergency use approval of ChAdOx1 nCoV-19 vaccine by the WHO (https://www.who.int/publications/i/item/WHO-2019-nCoV-vaccines-SAGE_recommendation-AZD1222-2021.1). The vaccine is made using the ChAdOx1 virus that has previously been administered to over 320 individuals and reported to be safe and tolerable (30). Chimpanzee adenovirus vaccine vectors have been safely administered to thousands of people using a wide range of infectious disease targets. Of relevance, a single dose of a ChAdOx1 vectored vaccine expressing full-length spike protein from another betacoronavirus (MERS-CoV) has been shown to induce neutralising antibodies in recent clinical trials (23). Additionally, adenoviral vector vaccines have been well studied and safely administered to participants aged 1 week to 90 years of age; this makes ChAdOx1 nCoV-19 a safe choice for individuals with pre-existing conditions who are reported to account for a majority of COVID-19 fatalities.

In order for the vaccine to be available for use in Kenya prior to the height of any future epidemic it is essential that phase I and II trials begin as soon as possible.

6. TRIAL OBJECTIVES AND PURPOSE

Objectives	Outcome measures	Time point(s) of evaluation of this outcome measure
**Primary Objectives**<br>To assess the safety, tolerability and reactogenicity profile of the candidate vaccine ChAdOx1 nCoV-19	Occurrence of serious adverse events (SAEs) throughout the study duration<br><br>Occurrence of solicited local and systemic reactogenicity signs and symptoms for 7 days following vaccination<br><br>Occurrence of unsolicited adverse events (AEs) at all scheduled visits;<br><br>Change from baseline for safety laboratory measures and;<br><br>Occurrence of SAE of special interest: disease enhancement episodes	Throughout the study<br><br>Day 0-7<br>Self-reported symptoms recorded using diaries and home / clinic visit<br><br>At all scheduled visits.<br><br>Blood samples drawn at enrolment (before vaccination), days 7 and 28 post-vaccination<br><br>Throughout the study
To assess immunogenicity of ChAdOx1 nCoV-19	ELISA to quantify IgG antibodies against SARS-CoV-2 spike protein (seroconversion rates)	Days 0 and 28 post-booster vaccination
**Secondary Objectives**<br>To assess humoral immunogenicity of	ELISA to quantify IgG antibodies against SARS-	See schedule of attendances (Tables 4)

ChAdOx1 nCoV-19 at early and late timepoints	CoV-2 spike protein (seroconversion rates)	
To assess cellular immunogenicity of ChAdOx1 nCoV-19	IFN-γ ELISpot responses to SARS-CoV-2 spike protein;	See schedule of attendances (Tables 4)
To assess efficacy of ChAdOx1 nCoV-19 against COVID-19	Virologically confirmed (PCR positive) symptomatic cases of COVID-19<br><br>Hospital admissions associated with COVID-19<br><br>Deaths associated with COVID-19<br><br>Seroconversion against non-Spike antigens measured by ELISA<br><br>Asymptomatic SARS-CoV-2 carriage	Throughout the study<br><br>Throughout the study<br><br>Throughout the study<br><br>Blood samples drawn at scheduled sampling timepoints (see Tables 4)<br><br>Nasopharyngeal samples taken at scheduled sampling timepoints (see Tables 4)
**Exploratory Objectives**<br>Exploratory Immunology	Virus neutralising antibody (NAb) assays against live and/or pseudotype SARS-CoV-2 virus<br><br>Cell analysis by flow cytometry assays<br><br>Functional antibody assays (e.g. antibody dependent cell mediated cytotoxicity)<br><br>Measurement of plasma cytokines and chemokines	See schedule of attendances (Tables 4)

7. TRIAL DESIGN

7.1. Overall Study Design and Plan Description

This phase Ib/II trial will be a single-blinded, randomized, controlled study of a single dose ChAdOx1 nCoV-19 vaccine among adults in Kenya. The study will be conducted among frontline staff (including Health care workers, Allied health professionals, truckers, security personnel, banking personnel, supermarket staff, Police, security personnel, Prison workers, Laboratory technicians, scientists, logistics personnel, public transport workers including aviation industry amongst others) in the coastal counties of Kilifi and Mombasa, where high numbers of COVID-19 cases have been detected. However, due to the risk of infection from ongoing community SARS-CoV-2 transmission in Kenya, other members of public will also be eligible for the study. The primary endpoints of the trial will be vaccine safety and immunogenicity of ChAdOx1 nCoV-19 vaccine as compared to the rabies control vaccine, with vaccine efficacy against COVID-19 evaluated as a secondary endpoint (see Section 6). Appropriate PPE will be used as applicable when performing study procedures. All participants and staff will follow prevailing government directives; currently wearing face masks while at the study clinics and ensure physical distancing whenever possible. In addition, level 2 PPE (e.g. NP95/FFP2/3 face mask, eye protection) will be used for all aerosol generating procedures.

Phase Ib trial: We will first evaluate the safety, tolerability and immunogenicity of the ChAdOx1 nCoV-19 (n=20) in comparison to the rabies control vaccine (n=20) in the phase Ib trial among healthy adults aged 18-55 years (Table 3a). Following screening, participants in groups 1a and 2a will be immunized on day 0 as outlined in Table 3a and undergo a 24-hour safety assessment. Provided there are no safety concerns, participants in groups 1b and 2b will be vaccinated no earlier than 24 hours after group 1a and 2a. Finally, if there are no safety concerns in groups 1a, b and 2a, b, after 72 hours, participants in groups 1c and 2c will be vaccinated no earlier than day 5 (Table 3a). Participants will be followed up daily for assessment of any adverse events during the first week. We will provide study participants with a thermometer, vaccination ruler and a diary. The follow-up will be done by phone call. No samples will be collected during this period, but the axillary temperature and any other significant symptoms will be recorded in the diaries. On day 7, the participants will visit the designated study clinic where physical assessment will be done, diary reviewed, and sampling done as per Table 4.

The DSMB will review safety data every two weeks for the first four weeks of the phase Ib trial and then subsequently at a frequency to be agreed between the trial investigators and the DSMB as the trial progresses. Should a grade 3 or more unsolicited AE (with no clear underlying cause) or a grade 4 solicited adverse event be identified, this would preclude progression from groups 1a/2a to 1b/2b or 1b/2b to 1c/2c. If vaccine safety is found to be satisfactory (after DSMB review of safety data accrued up to day 28 following vaccination of participants in group 1a and 2a), we will begin the phase II trial. During the scheduled clinic visits (see Table 4), physical and clinical assessment, recording of medical history, vital signs and concomitant medication will be done. We will also collect nasopharyngeal, and throat swabs for monitoring of SARS-CoV-2 infection and assessment of mucosal immunity, and 50ml of blood for immunological analyses (Table 4).

Higher levels of immune responses have been observed following a booster immunization with ChAdOx1 nCoV-19 in the UK trial (28). Phase Ib trial participants will receive a booster dose of vaccine 3 months after the first dose (ChAdOx1 nCoV-19 for groups 1a, 1b and 1c, and rabies for groups 2a, 2b and 2c). Follow up will be for 12 months from the first dose of vaccine. Phase II trials will evaluate the safety and immunogenicity of this 3-month prime-boost regimen.

Table 3a: Schedule for the phase Ib trial

Group	Vaccine	Day of study (first dose)	Day of study (booster dose)
**1a (n=1)**	$5x10^{10}$ vp ChAdOx1 nCoV-19	0	3 months after the first dose
**2a (n=1)**	Rabies vaccine	0	
24 hours safety assessment (DSMB safety review)			
**1b (n=3)**	$5x10^{10}$ vp ChAdOx1 nCoV-19	2	
**2b (n=3)**	Rabies vaccine	2	
72 hours safety assessment (DSMB safety review)			
**1c (n=16)**	$5x10^{10}$ vp ChAdOx1 nCoV-19	5	
**2c (n=16)**	Rabies vaccine	5	
**The DSMB will meet to review the data every two weeks for the first four weeks of the phase Ib trial and then subsequently at a frequency to be agreed between the trial investigators and the DSMB as the phase Ib/II trial progresses**			

Phase II trial: We will assess immunogenicity of $5x10^{10}$vp ChAdOx1 nCoV-19 vaccine in comparison to rabies vaccine in 360 adults aged ≥18 years (180 per vaccine group; Table 3b). Participants will be randomized to receive two doses of vaccine 3 months apart as outlined in Table 3b. Follow up will be for 12 months from the first dose of vaccine. During the scheduled clinic visits (see Table 4), physical and clinical assessment, recording of medical history, vital signs and concomitant medication will be done. We will also collect nasopharyngeal, and throat swabs for monitoring of SARS-CoV-2 infection and assessment of mucosal immunity, and 50ml of blood for immunological analyses (Table 4).

Table 3b: Schedule for the phase II trial

Group	Vaccine (day 0)	Vaccine (day 84)
**3 (n=180)**	$5x10^{10}$ vp ChAdOx1 nCoV-19	$5x10^{10}$ vp ChAdOx1 nCoV-19
**4 (n=180)**	Rabies vaccine	Rabies vaccine

Care of participants with symptoms consistent with COVID-19: In the event that any of the study participants present with symptoms consistent with COVID-19 we will immediately notify public health authorities to facilitate their sampling, clinical management and quarantine as per the prevailing national guidelines. Though these Ministry of Health (MoH) personnel will not be on the trial delegation log they may perform the sampling of study participants suspected or confirmed to have COVID-19. This will be a pragmatic activity in order to keep with the prevailing national directives. As per current government procedures participants will either be quarantined in the COVID-19 complex in Kilifi or in the Technical University of Mombasa and the costs covered by the government. The location of quarantine and isolation may change. However, during isolation and quarantine we will reimburse the cost of meals where these may not be fully covered. The government has also implemented home-based care for mild cases, and we anticipate this will cover the majority of our participants who develop infection. Public health authorities will also be notified of participants whose nasopharyngeal/throat swabs are found to be positive for SARS-CoV-2 in the absence of symptoms. These notifications to the MoH will be done through the existing framework for reporting COVID-19 test results at KEMRI CGMRC, the designated COVID-19 testing centre for coastal Kenya. Participants who test positive for COVID-19 / asymptomatic SARS-CoV-2 infection will remain in follow up (including during isolation) until the

end of the study and their clinical outcome documented. Any required sampling during the isolation period will be done by designated personnel, who may be from the MoH or study team depending on the prevailing government directives all of whom are trained in collection of nasopharyngeal/throat swabs.

Table 4: Study schedule of activities (Phase Ib and II)

**Event**	**Screening**	**D0**	**D1-D6**	**D7**	**D14**	**D28**	**D84**	**D85-D90**	**D91**	**D112**	**D182**	**D365**
Visit	**VS**	**V1**		**V2**	**V3**	**V4**	**V5**		**V6**	**V7**	**V8**	**V9**
Window (days)	**-30**			**+1**	**±1**	**+3**	**±7**		**+1**	**±7**	**±14**	**±14**
Obtain written Informed Consent/e-consent	X											
Demography	X											
Medical History	X	X		X	X	X	X		X	X	X	X
Physical Examination	X	X		X	X	X	X		X	X	X	X
Anthropometry	X											
Urine for Glucose	X											
β-HCG urine (♀)	X	X										
Vital signs^	X	X		X	X	X	X		X	X	X	X
Vaccination		X					X					
Randomization		X										
Diary cards		X	X	X				X	X			
Solicited AEs		X	X	X				X	X			
Unsolicited AEs		X	X	X	X	X	X	X	X	X	X	X
Safety bloods[$]	X	X*		X		X	X		X	X		
Nasopharyngeal/throat swabs	X	X*			X	X	X		X	X	X	X
Immunology bloods		X			X	X	X		X	X	X	X
Blood volume per visit (mL)**	10	50	0	10	50	50	50	0	50	50	50	50
Cumulative blood** volume (mL)	10	60	60	70	120	170	220	220	270	320	370	420

^ = Vital signs includes pulse, blood pressure, oxygen saturation, respiratory rate and temperature; $ = Biochemistry will include Sodium, Potassium, Urea, Creatinine and Liver function tests (Albumin, ALT, ALP, Bilirubin) and

Haematology tests will include; Full blood count. *Pre-vaccination (Day 0) safety bloods and nasopharyngeal/throat swabs only repeated if more than 14 days (bloods) or 96 hours (swab) elapse between screening and an eligible and willing participant presenting for enrolment. **Represents maximum volumes for the first 120 study participants, with 50ml sampling allowing for cellular immunology work. For the remaining 280 study participants, the maximum volume collected for immunology will be 10ml at each scheduled visit (total cumulative blood volume of 100mL).

7.2. Study procedures (similar for phase Ib and phase II trial)

7.2.1. Recruitment

Recruitment for this study will be focused on frontline staff (including Health care workers, Allied health professionals, truckers, security personnel, banking personnel, supermarket staff, Police, security personnel, Prison workers, Laboratory technicians, scientists, logistics personnel, public transport workers including aviation industry amongst others) aged ≥18 years resident in the coastal counties of Kilifi and Mombasa. However, due to the risk of infection from ongoing community SARS-CoV-2 transmission in Kenya, other members of public will also be eligible for the study. Individuals included in other studies of COVID-related interventions will not be eligible for inclusion to this vaccine trial. We will put in place measures to avoid co-recruitment of participants to more than one study. Following public and community engagement activities detailed in section 17.2 information sheets will be given to interested volunteers. Posters will also be used to give study information to prospective participants. These posters will be put up in areas where the frontline staff work, public notice boards, and workplace intranet advertising spaces. Those willing to participate in the study will be asked to travel to the designated study clinic (after pre-screening) where the consent process, screening and enrolment into the study will be conducted by a clinician. Investigators will emphasize that participation in the study is voluntary. Potential participants will be screened for good health to identify the minimum number required for each group; we will screen potential volunteers until we reach the number required for enrolment. It will be made clear to all potential participants that they may be excluded for several health reasons even for conditions that may have relatively little impact on their day to day lives. Eligible participants for phase Ib who are not enrolled due to the target number being reached will be invited to phase II. 28 days following first vaccination of group 1a the study will pause enrolment whilst the DSMB review the safety data before moving to phase II of the trial. We will attempt to retain participants by facilitating study visits and making participation as positive an experience as possible. Where individuals choose not to take part, we will respect that, and no explanation is required should they wish to withdraw.

7.2.2. Informed consent/E-consent

Prior to full screening procedures participants will be required to provide informed consent, pre-screening will be conducted to minimise congestion. Appropriately trained staff will take the participant through the informed consent form (ICF) that will be developed specifically for this study. E-consent is a mitigation to minimize transmission for SARS-CoV-2. E-consent can be self-administered with study team members to address queries at any time during the process. Consent may be obtained in-person, self-administered online using supportive software (e.g. REDCap or on the phone).

All consent will have to be written/signed as per our consent SOP. The paper/electronic ICF will be translated into Kiswahili. Potential participants will have ample time to read the patient information sheet. Information regarding the study will be disseminated either in person on an individual basis, via email, on the phone or in a socially distanced group session where potential participants will be encouraged to ask questions. After this session, each participant will have a private discussion with trained study staff where additional clarification will be provided and time for more questions provided. The staff will assure potential participants of confidentiality. We will share the Patient Information Sheet and ICF with potential participants who contact us by email, and they will be free to read and sign this prior to attending clinic although all consent will be verified on site and a written signed copy shared with the study team or signed in person on the day. They will complete a test of understanding either online or in clinic which again will be verified. During the informed consent process, we will emphasize the following:

- Participation in the study is entirely voluntary.
- Declining to participate involves no penalty or loss of medical benefits.
- A volunteer may withdraw from the study at any time.
- A volunteer is free to ask questions at any time to allow him or her to understand the purpose of the study and the procedures involved-irrespective of the nature in which informed consent is obtained.
- There may be no direct benefit from participating, other than knowledge of their general health status. The benefits will be realized in the long-term for the community by contributing towards the development of an effective COVID-19 vaccine.
- Volunteers will be compensated for travel, time and inconvenience of participating.

- We will notify public health authorities if they are found to have COVID-19 or asymptomatic SARS-CoV-2 infection during the study.

During the informed consent process, we will emphasize to the participants that the vaccine efficacy is yet to be proven. As such, the participants will be advised to continue with the current preventive measures for COVID-19 as outlined by the MoH e.g. physical distancing, coughing and sneezing etiquette, hand washing and avoiding touching of the face. The informed consent process will ensure that potential participants have an understanding of the potential risks and benefits of participating in the study, the study procedures (including maintaining confidentiality and anonymity), study assessment schedule, the use of the blood samples, their right to refuse and/or withdraw from the study at any point without affecting any of the other health services or care they receive, without having to disclose a reason for their refusal or withdrawal. All participants will sign and date the informed consent form before any study specific procedures are performed. Participants will be expected to complete a test of understanding of the study. If consent is obtained, the screening procedures described below and outlined in Table 4 will be undertaken.

7.2.3. Screening

Abbreviated pre-screening of individuals interested in participating will be conducted by phone (i.e. when the individuals get in touch with the study team as advertised on the flyers) prior to attending clinic to minimise congestion. We intend to recruit healthy adults aged 18 to 55 years for the phase Ib trial, and all adults aged ≥18 years for the phase II trial. Screening may take place up to 30 days prior to enrolment. Participants will be screened for clinically significant acute or chronic diseases such as congenital heart disease, renal failure, hepatitis, HIV, Hepatitis B and C, chronic respiratory conditions, hypertension, diabetes amongst others based on the study inclusion and exclusion criteria and the clinical judgment of the clinician. All participants will have pre-test counselling for Hepatitis and HIV as per local practices and guidelines. Screening will be based on physical examination and laboratory tests; complete blood count and biochemistry. Abnormal clinical findings from the medical history, clinical assessment, or blood tests at any point in the study will be assessed. We will recruit participants with no clinically significant illness. If a test is deemed clinically significant it may be repeated to ensure it is not a single occurrence. If an abnormal finding is deemed to be

clinically significant and warrant referral, the volunteer will be informed and offered referral to an appropriate medical centre.

Participants will also be counselled by a trained member of the study team for screening for HIV and results feedback accordingly. To maintain the confidentiality of those potential participants infected with HIV, we will make it clear during screening that one can be excluded due to a range of diseases (not just HIV) as well as abnormal laboratory results. Prior to vaccination, female participants will be tested for pregnancy and excluded if confirmed. Once all specific visit procedures are completed, results will be fed back in a timely manner during a feedback visit if the participant is not to take part. These procedures will be documented in the case report form (CRF) and relevant clinical notes, which will be kept in the individual study participant's file.

7.2.4. Randomization/Enrolment

Participants who meet all the inclusion and none of the exclusion criteria will be enrolled in the study. After eligibility is confirmed, the participant will be randomized once they complete their pre-vaccination assessment successfully. The randomization list will be generated using a statistical software (Stata/R) and random allocation done using REDCap and its access will be restricted to the unblinded team. Participants will be allocated to one of the two vaccine arms as per the computer-generated randomization schedule. To minimize vaccine wastage, randomization will be done in block sizes that match with clinic visit days. Participants will not be informed of the allocated vaccine. Vaccination will be administered as outlined in Table 3a and Table 3b. The following procedures will take place before vaccination:

- Review of the inclusion and exclusion criteria to confirm eligibility.
- Targeted medical history since previous visit.
- Physical examination.
- Vital signs.
- Record any concomitant medication.
- Safety and immunology blood collection.
- Nasopharyngeal and throat swab collection.
- Urine pregnancy test for female participants.
- Randomization.
- Record pre-vaccination axillary temperature.

Participants will then be vaccinated with either ChAdOx1 nCoV-19 or rabies vaccine and observed post-vaccination for at least one hour to:

- Record vital signs and post-vaccination axillary temperature.
- Record any solicited and unsolicited AEs before the participant leaves the site.
- Instruct the participant to contact the study team in case they develop any symptoms.
- Given a thermometer, measuring ruler and diary card and show how to fill it appropriately.
- Given emergency contact numbers of the study team member on call in the event of an emergency or inquiries.
- Schedule the next appointment.

A participant will be considered enrolled once they receive the allocated vaccination. Vaccine vials will be handled as per manufacturer's instructions, with cold storage verified by a temperature tracker. Table 5 outlines the local and systemic solicited AEs that will be assessed during the week post vaccination. These will be documented on the diary cards/e-diary, which will have sections for documenting the timing and severity of solicited and unsolicited AEs.

Table 5: Solicited AEs as collected on post vaccination diary cards

**Local Solicited AEs**	**Systemic Solicited AEs**
Pain	Fever
Tenderness	Feverishness
Redness	Chills
Warmth	Joint pains
Itch	Muscle pains
Swelling	Fatigue
Induration	Headache
	Malaise
	Nausea

7.2.5. Scheduled visits

Following each vaccination, study participants will be contacted daily from days 1-6 via phone by the study team to collect information on any new symptoms or concomitant medication and remind them to fill the diary cards. Participants will also be instructed to contact the study team at any time should they experience any clinical symptoms. 7 days after each vaccination, participants will attend for a clinic visit and return the diaries. Information collected during the daily phone call follow-up and at the 7 days post-vaccination clinic visit will include:

- Record information on any post-vaccination solicited and unsolicited AEs.
- Record axillary temperature.
- Completion of the diary cards.
- Record any AEs or concomitant medication in the respective forms.

For all remaining visits vital signs will be taken, adverse events and concomitant medication recorded, and sampling done as per Table 4. If a participant does not appear for an assigned visit, every effort will be made to contact them to confirm that they are well and to schedule a visit. This effort will be documented into the source data. At the day 365 visit, an end of study CRF will be completed and participants informed of study completion.

7.2.6. Unscheduled Visits

Prior to leaving the study clinic, the participants will be advised to contact the study team if they experience any symptoms of concern during or out of working hours. Such visits will be documented in the unscheduled visit CRF and a corresponding concomitant medication CRF. Data on AEs and SAEs will be collected as described in later sections below. Female participants becoming pregnant during the follow-up period will be referred for ante-natal visits to health facilities of their choice and pregnancies will be followed-up (at least at every antenatal clinic) and outcome recorded within 72 hours of delivery and at a 6-week post-natal check. Congenital anomalies will be reported as SAEs.

In the event that any of the study participants develop symptoms consistent with COVID-19 during the study, or if they are found to carry SARS-CoV-2 in the absence of symptoms, we will immediately notify public health authorities to facilitate with their sampling, clinical management and quarantine as described in section 7.1. The Government of Kenya is

currently covering all costs of hospitalization for patients diagnosed with COVID-19. The study will cover costs for management of acute illnesses and of admission to public facilities for any other serious adverse events during the 12 months duration of participation in the trial. Medical care will be provided within the Kenyan Ministry of Health guidelines. Medical emergency plans will be in place with the possibility to admit patients to the Aga Khan Hospital in Mombasa for vaccine-related serious adverse events requiring intensive care.

7.2.7. End of Treatment Visit

To reduce loss to follow-up at the 6-month (day 182) and 12-month (day 365) visit, the study team will contact the participants a month earlier to remind them about the remaining follow-up visit. The 12-month visit will be the final study visit. During the last visit, a blood sample and nasopharyngeal/throat swab will be taken, and the participants' general health status recorded. All study participants will be offered an optional full course of the control rabies vaccine at the end of the study. Depending on the results at the end of the study, we may contact participants to enable long-term (>1 year) follow-up of immunogenicity and efficacy. An amendment to the protocol with details of any planned long-term follow up will be made and submitted to SERU, OXTREC and PPB for review and approval. The control group will be offered the ChAdOx1 nCoV-19 vaccine at the end of the trial. If the vaccine is licensed at this point, then this will be done as a non-research procedure (or after a short delay if licensing is under consideration). If the vaccine is still not licensed, then the investigators will write to SERU, OxTREC and PPB to ask their input on the appropriateness of vaccination of the control group based on all available global data on safety and efficacy. Use of the unlicensed vaccine will require a protocol amendment in order to allow vaccination to take place as a research procedure. The required monitoring follow up and safety assessments after vaccination will depend on the data available from the trial and from other related trials (e.g. UK, Brazil and South Africa), and will be specified in full in the amendment.

7.2.8. Study Restrictions/ holding rules

Since the approval of ChAdOx1 nCoV-19 for emergency use by PPB and its subsequent national roll-out, group holding rules will no longer apply. Any SUSARs occurring in the trial from the time of approval onwards will be notified to and discussed with the DSMB and PPB but will no longer automatically trigger a holding rule.

In case of study termination, the investigators will be informed by the regulatory authority or other approved body of the procedures to be followed to ensure that, adequate consideration is given, to the protection of the participant's safety. The Principal Investigator with support of the sponsor will be responsible for informing IRB/ECs and for notifying the health authorities/regulatory authorities of the early termination of the trial.

8. SELECTION AND WITHDRAWAL OF STUDY PARTICIPANTS

8.1. Description of the population to be studied and eligibility criteria

The study will be conducted among frontline staff (including healthcare workers, allied health professionals, truckers, security personnel, banking personnel, supermarket staff, police, security personnel, prison workers, laboratory technicians, scientists, logistics personnel, public transport workers including aviation industry amongst others) in the coastal counties of Kilifi and Mombasa, where high numbers of COVID-19 cases have been detected. However, due to the risk of infection from ongoing community SARS-CoV-2 transmission in Kenya, other members of public will also be eligible for the study. A total of 400 participants will be enrolled and out of these, 40 will be enrolled in the phase Ib trial (Table 3a), and the remainder to the phase II trial (180 per vaccine; Table 3b). The adults must be healthy and aged ≥18 to 55 years for phase Ib, and ≥ 18 years for phase II. Continuous eligibility will be reviewed and confirmed by the study team at specific visits. The following inclusion and exclusion criteria will be confirmed during the screening visit:

8.2. Inclusion criteria

- Frontline Staff as defined by the Government of Kenya (including healthcare workers, allied health professionals, truckers, security personnel, banking personnel, supermarket staff, police, security personnel, prison workers, laboratory technicians, scientists, logistics personnel, public transport workers including aviation industry amongst others) and other members of public
- Healthy adults aged 18-55 years for phase Ib, ≥18 years for phase II.
- Able and willing (in the Investigators' opinion) to comply with all study requirements, including making visits to the designated study clinic for follow up under conditions with limited transport.
- Agreement to refrain from blood donation during the course of the study

- Use of effective method of contraception for duration of study for female participants. They should use effective contraception for 30 days prior to vaccination. For female participants, we will ask them to attend with their family planning records for verification. Effective contraception is defined as a contraceptive method with failure rate of less than 1% per year when used consistently and correctly, in accordance with the product label. Examples of these include: combined oral contraceptives; injectable progestogen; implants of etenogestrel or levonorgestrel; intrauterine device or intrauterine system; male partner sterilisation at least 6 months prior to the female subject's entry into the study, and the relationship is monogamous; male condom combined with a vaginal spermicide (foam, gel, film, cream or suppository); and male condom combined with a female diaphragm, either with or without a vaginal spermicide (foam, gel, film, cream, or suppository).
- Provide written informed consent.
- Plan to remain resident in the study area for 1 year following vaccination

8.3. Exclusion criteria

- Prior receipt of any vaccines (licensed or investigational) ≤30 days before enrolment.
- Previous receipt of a covid-19 vaccine.
- Volunteer who is not literate.
- Planned receipt of any vaccine other than the study intervention within 30 days before or after study vaccination.
- Prior receipt of an investigational or licensed vaccine likely to impact on interpretation of the trial data (e.g. Adenovirus vectored vaccines, any coronavirus vaccines).
- Planned or ongoing participation in any other interventional studies (of licensed or investigational products) ≤30 days before enrolment and for the duration of the study.
- Administration of immunoglobulins and/or any blood products within the three months preceding the planned administration of the vaccine candidate.

- Any confirmed or suspected immunosuppressive or immunodeficient state, including HIV infection; asplenia; recurrent severe infections and chronic use (more than 14 days) immunosuppressant medication within the past 6 months (inhaled and topical steroids are allowed).
- History of allergic disease or reactions likely to be exacerbated by any component of the vaccine.
- Any history of hereditary or idiopathic angioedema.
- Pregnancy, lactation or willingness/intention to become pregnant during the study.
- History of cancer (except basal cell carcinoma of the skin and cervical carcinoma in situ).
- History of serious psychiatric condition likely to affect participation in the study.
- Bleeding disorder (e.g., factor deficiency, coagulopathy or platelet disorder), or prior history of significant bleeding or bruising following IM injections or venepuncture.
- Any other serious chronic illness requiring hospital specialist supervision.
- Suspected or known current alcohol abuse as defined by an alcohol intake of greater than 42 units every week (e.g., more than 2 bottles of 500mls Tusker (beer) a day, more than 2 large glasses of 12% wine per day).
- Suspected or known injecting drug abuse in the 5 years preceding enrolment.
- Any clinically significant abnormal finding on screening biochemistry, haematology blood tests or urinalysis.
- Any other significant disease, disorder or finding which may significantly increase the risk to the volunteer because of participation in the study, affect the ability of the volunteer to participate in the study or impair interpretation of the study data.
- History of laboratory-confirmed COVID-19 or SARS-CoV-2 infection.
- New onset of fever and a cough or shortness of breath in the 30 days preceding screening and/or enrolment.

8.4. Managing withdrawals

The participants have a right to withdraw from the study at any time and for any reason without penalty. In the absence of a medical contraindication or significant protocol violation, every effort will be made by the investigator to keep the participant in the study

and ensure they attend safety follow-up. An excessive rate of withdrawals can render the study non-interpretable. If a participant withdraws, the PI will make a reasonable effort to determine the reason for the request. Telephone calls and physical tracing (in line with institutional and government directives) are considered reasonable efforts. The PI may withdraw a participant from participating in the study without the participant's consent if any of these criteria are met:

- A participant fails to comply with study procedures and/or inclusion/exclusion criteria
- A participant's safety or health may be compromised by further participation
- It is determined to be in the participant's best interest.
- Participant withdraws consent.
- On the advice of the DSMB
- Any adverse event which results in the inability to comply with study procedures.
- Significant protocol violation.
- Loss to follow up (applies to a subject who consistently does not return for scheduled study visits, is not reachable by telephone or any other means of communication and/ is not able to be located).
- Ineligibility either arising during the study or retrospectively (having been overlooked/missed during screening).

Data and samples retrieved before the withdrawal of consent will be used as study data unless the participant requests otherwise. Similarly, any samples collected prior to their withdrawal will be used or stored unless requested otherwise. The reason for withdrawal will be recorded in the study completion CRF. In cases where the withdrawal is due to SAEs, the investigators will arrange for the appropriate specialist management and notify the regulatory authority. In such cases, the extent of follow-up will be based on the assessment of a medically qualified investigator. Once a participant is withdrawn by the PI, they will not be required to continue with the remaining study visits but may be requested to come to the clinic for safety assessment.

8.5. Replacing withdrawn participants

Participants will not be replaced following withdrawal.

9. TREATMENT OF STUDY PARTICIPANTS

9.1. ChAdOx1 nCoV-19 vaccine

ChAdOx1 nCoV-19 formulated and vialed under Good Manufacturing Practice conditions at ADVENT S.R.L., Italy will be used for the phase Ib trial. At the University of Oxford Clinical Biomanufacturing Facility (CBF) the vaccine will be certified and labelled for the trial by a Qualified Person (QP) before transfer to KEMRI CGMRC.
ChAdOx1 nCoV-19 (AZD1222) formulated at Cobra Biologics Ltd, vialed at Symbiosis Pharmaceutical Services, and labelled and packaged at Thermo Fisher Scientific (Hertfordshire, United Kingdom) will be used for the phase II trial. It will be certified by a Qualified Person (QP) at the MedImmune Pharma, BV (Nijmegen, The Netherlands) or MedImmune Ltd (Cambridge, United Kingdom) before release and transfer to the clinical site.

9.2. Storage

The vaccine manufactured by Advent s.r.l. will be stored at nominal -80ºC (+/-20ºC) in a locked freezer, at the clinical site. The vaccine manufactured by Cobra Biologics Ltd will be stored at 2-8°C in a secure fridge, at the clinical site. All movements of the study vaccines will be documented in accordance with existing standard operating procedure (SOP). Vaccine accountability, storage, shipment and handling will be in accordance with relevant SOPs and forms.

9.3. Administration

The vaccine will be administered intramuscularly in the deltoid of the non-dominant arm unless there is a contradiction (e.g. large keloid scar at site) which would be documented on the CRF. The control vaccine will also be administered on the same site as the investigational product to maintain blinding. This information will be recorded in the CRF. Additional information on the study product and administration is included in the Investigator's Brochure.

9.4. Timing and selection of Doses

Participants will receive $5x10^{10}$ vp ChAdOx1 nCoV-19 vaccine. The DSMB will review the safety data prior to starting the phase II trial. Should any individual safety concern(s) arise a

local safety monitor will be available to conduct an independent assessment and will defer and provide input to the DSMB.

9.5. Rationale for the selected doses

The dose to be administered in this trial has been selected on the basis of clinical experience with the ChAdOx1 vector expressing different inserts (Table 1) and other similar adenovirus vectored vaccines (e.g. ChAd63). A first-in-man dose escalation study using the ChAdOx1 vector encoding an influenza antigen (FLU004) safely administered ChAdOx1 NP+M1 at doses ranging from 5 x 10^8 to 5 x 10^{10} vp. Subsequent review of the data identified an optimal dose of 2.5 x 10^{10} vp balancing immunogenicity and reactogenicity. This dose has subsequently been given to over hundreds of volunteers in numerous larger phase 1 studies at the Jenner Institute (see Table 1). ChAdOx1 vectored vaccines have thus far demonstrated to be very well tolerated. The vast majority of AEs have been mild-moderate and there have been no SARs until this date.

Another simian adenovirus vector (ChAd63) has been safely administered at doses up to 2 x 10^{11} vp with an optimal dose of 5 x 10^{10} vp, balancing immunogenicity and reactogenicity. MERS001 was the first clinical trial of a ChAdOx1 vectored expressing the full-length Spike protein from a separate, but related betacoronavirus. ChAdOx1 MERS has been given to 31 participants to date at doses ranging from $5x10^9$ vp to $5x10^{10}$ vp. Despite higher reactogenicity observed at the $5x10^{10}$ vp, this dose was safe, with self-limiting AEs and no severe adverse reactions recorded. The $5x10^{10}$ vp was the most immunogenic, in terms of inducing neutralising antibodies against MERS-CoV using a live virus assay (23). Given the immunology findings and safety profile observed with a ChAdOx1 vectored vaccine against MERS-CoV, the $5x10^{10}$ vp dose was chosen for ChAdOx1 nCoV-19.
Preliminary results following ChAdOx1 nCoV-19 vaccination of 543 adult volunteers in the UK indicate that the $5x10^{10}$ vp dose safely elicits a robust antibody and T cell response, and that these responses increase in magnitude following a second dose of $5x10^{10}$ vp ChAdOx1 nCoV-19 vaccine (28). No SARs or SUSARs definitively associated with ChAdOx1 nCoV-19 have been reported to date (see Investigator's Brochure). ChAdOx1 nCoV-19 has received Emergency Use Authorisation from various national and international regulators, including the WHO.

9.6. Packaging and Labelling

All study vaccines were manufactured and packaged in accordance with Good Manufacturing Practice (GMP). The ChAdOx1 nCoV-19 vaccine will be packaged and labelled under the responsibility of the Sponsor. Each vial and box containing the investigational product will be labelled for identification with a primary and a secondary label, respectively. ChAdOx1 nCoV-19 formulated and vialed under Good Manufacturing Practice conditions at Advent s.r.l., Italy will be used in the phase Ib trial. At the University of Oxford Clinical Biomanufacturing Facility (CBF) the vaccine will be certified and labelled for the trial by a Qualified Person (QP) before transfer to the clinical site. ChAdOx1 nCoV-19 (AZD1222) formulated at Cobra Biologics Ltd, vialed at Symbiosis Pharmaceutical Services, and labelled and packaged at Thermo Fisher Scientific (Hertfordshire, United Kingdom) will be used for the phase II trial. It will be certified by a Qualified Person (QP) at the MedImmune Pharma, BV (Nijmegen, The Netherlands) or MedImmune Ltd (Cambridge, United Kingdom) before release and transfer to the clinical site.

ChAdOx1 nCoV-19 manufactured at Advent s.r.l. is formulated in formulation buffer (10mM Histidine, 7.5% sucrose, 35mM NaCI, 1mM MgCl2, 0.1% PS80, 0.1mM EDTA, 0.5% EtOH, pH 6.6) at a target concentration of $\geq 1.0 \times 10^{11}$ vp/mL. The drug product is filled into 2mL glass vials with a 13mm grey bromobutyl rubber freeze-dry stopper (CE Marked, supplied by Adelphi Healthcare Packaging Tubes) and a 13 mm complete tear, clear lacquered aluminium seal. The nitrogen filled vials are supplied sterile. The containers and closures are tested for compliance with defined specifications. The vials are made from Ph Eur Type 1 glass. The fill volume is 0.35mL to 1mL. ChAdOx1 nCoV-19 (AZD1222) manufactured at Cobra Biologics Ltd is vialed as solution for injection stored at 2–8°C intended for intramuscular administration. AZD1222 is formulated at $>0.7 \times 10^{11}$ vp/mL in 10 mM histidine, 7.5% (w/v) sucrose, 35 mM sodium chloride, 1 mM magnesium chloride, 0.1% (w/v) polysorbate 80, 0.1 mM edetate disodium (EDTA), 0.5% (v/v) ethanol, pH 6.6. The investigational product is supplied as a sterile, clear to slightly opalescent solution, practically free from visible particles, in a 10R vial at a label-claim volume of 5 mL, stoppered with an elastomeric stopper, and sealed with an aluminium overseal. Each multi-dose vial can provide up to ten 0.5 mL doses.

9.7. Control vaccine

In order to provide a benefit to study participants, a vaccine currently on the market and recommended but not covered in the national immunization program will be administered to participants of the control group instead of placebo. Verorab (from Sanofi) will be used as the rabies vaccine and will be purchased locally by the study site and maintained under controlled temperatures as outlined in the Specific Product Characteristics. The rabies vaccine has been reported to have a reassuring safety profile and would offer some benefits to the participants given the risk of rabies in the country. One dose consists of the administration of 0.5 mL of vaccine via the intramuscular route.

9.8. Dispensing procedures

The vaccination nurse and pharmacist will be unblinded and will not disclose the vaccine to the participants and will be responsible for transporting the vaccines from the storage area to the vaccination rooms. The participant, clinicians, nurses and all staff/investigators assessing the safety endpoints in the trial will be blinded to the allocation. The ChAdOx1 nCoV-19 vaccine is a slightly opaque frozen liquid, essentially free from visible particulates. The appearance is dependent upon the concentration of the virus and the buffer that the virus is formulated in. Vaccines will be prepared out of sight of the participant and syringes will be masked with an opaque object/material until ready for administration to ensure blinding. In addition, they will ensure that the vaccines are transported in accordance to the temperature requirements and any excursions reported immediately. Study vaccines will be labelled as per the Sponsor's requirements and will be reconstituted after randomization.

9.9. Dose Administration

The pharmacist and nurse will be responsible for preparation and administration of the vaccines as randomized. They will be responsible for the accompanying documentation. The vaccine will be administered into the non-dominant arm and vaccine preparation will be undertaken under aseptic non-touch conditions by the pharmacist and nurse.

Advent s.r.l. material, phase Ib trial: On vaccination day, ChAdOx1 nCoV-19 will be allowed to thaw to room temperature and will be administered within 1 hour of removal from the freezer or stored at 2-8°C for a maximum of 6 hours, where multiple doses are required from a single vial. Stability data has been generated to support this from ChAdOx1 vaccines (refer to

IMPD for stability programmes). Vaccination will be performed according to the relevant SOPs.

Cobra Biologics Ltd material, phase II trial: The vaccine must be stored at 2–8°C at all times unless it is being used for the dose preparation process. Total time from needle puncture of the ChAdOx1 nCoV-19 (AZD1222) vial to the start of administration must not exceed 6 hours at room temperature (25°C). If preparation time exceeds the time limit a new dose must be prepared. AZD1222 does not contain preservatives, each vial must be assigned a beyond use date of 48 hours from first needle puncture of the AZD1222 vial, after which any unused portion must be discarded. Each vial of AZD1222 has a label-claim volume of 5 mL and can provide up to ten 0.5 mL doses. Each dose is prepared by withdrawing 0.5 mL from a vial of AZD1222 in a sterile 1 mL or equivalent syringe.

9.10. Accountability

The PI or responsible personnel will document and file records of clinical supplies for the study. At any time, the figures of supplied, used, and remaining vaccines should match. At the end of the study, a reconciliation of the delivery records with those of used and unused stocks will be performed and documented. There will be accountability logs kept in the pharmacy or storage area as well as during the vaccination sessions. These will be reconciled at the end of each day.

9.11. Unblinding

Unblinding will be done at the end of the trial. Participants who receive the ChAdOx1 nCoV-19 or rabies vaccine will be offered a complete course of the rabies vaccine at the end of the trial. Participants will be advised that should they be exposed at any point during or after the study a full post-exposure course will be required. If the clinical condition of a participant necessitates unblinding, this will be undertaken according to a trial specific working instruction and group allocation sent to the attending clinician, if unblinding is thought to be relevant and likely to change clinical management. In the event of COVID-19 vaccines becoming approved for use by PPB, participants will be supported to access COVID-19 vaccines as early as possible. Participants who are eligible (as per government prioritisation strategy) and have been invited to receive an approved or licensed COVID-19 vaccine can request to be unblinded. All unblinded participants will be kept in the study regardless of the

COVID-19 vaccines received. Follow up visits will continue as per the participants previous schedule and their data will contribute to ongoing safety monitoring according to the vaccine combinations they have received. Participants will be censored in the analysis of efficacy endpoints at the time of their unblinding and vaccination but will contribute to exploratory immunological analyses which are descriptive or observational according to the vaccines they have received.

9.12. Prior/Concomitant Therapy

Any concomitant therapy will be documented in the appropriate CRF. Unblinded participants that have received an approved or licensed vaccine as part of the government vaccine roll-out will be asked about the vaccine they received and date of administration, and the information recorded as a concomitant medication.

10. ASSESSMENT OF SAFETY

10.1. Adverse Events (AEs)

The recording of AEs is an important aspect of study conduct and must be appropriately documented. The investigators will be responsible for appropriately documenting all AEs according to the detailed guidelines set out below. The participants will be instructed to contact the study team immediately should they manifest any signs or symptoms they perceive as severe. The PI/designee is responsible for the routine evaluation of safety aspects of the study. This includes the review of all available information. Emergency equipment and medications will be available within the clinical unit and their use in the context of the study will be documented in the source documents and then in the CRF.

10.2. Definitions and monitoring of AEs

Definitions of AEs

Adverse Event (AE)

Any untoward medical occurrence in a patient or clinical investigation subject occurring in any phase of the clinical study whether considered related to the vaccine. This includes an exacerbation of pre-existing conditions or events, intercurrent illnesses, or vaccine or drug interaction. Anticipated day-to-day fluctuations of pre-existing conditions, that do not represent a clinically significant exacerbation will not be considered adverse events. Discrete

episodes of chronic conditions occurring during a study period will be reported as adverse events (following receipt of a study vaccine) to assess changes in frequency or severity. Adverse events will be documented in terms of a medical diagnosis/diagnoses. When this is not possible, the adverse event will be documented in terms of signs and symptoms observed by the investigator or reported by the subject at each study visit. Pre-existing conditions or signs and/or symptoms (including any which are not recognised at study entry but are recognised during the study period) present in a subject prior to the start of the study will be recorded on the Medical History form. Definitions of AEs and grading, when applicable, to be followed will be specified in a study specific procedure (SSP). Malaria will be diagnosed and treated as per national guidelines. There is some concern but no evidence that malaria may have an impact on COVID-19 disease. We will collect all AEs and SAEs and these data will be correlated with safety and immunogenicity outcomes.

Adverse Reaction (AR)

All noxious and unintended responses to a medicinal product related to any dose should be considered adverse reactions.

Serious Adverse Event (SAE)

A serious adverse event is any untoward medical occurrence that at any dose:

- results in death,
- is life-threatening,

  Note: The term “life-threatening” in the definition of “serious” refers to an event in which the patient was at risk of death at the time of the event; it does not refer to an event which hypothetically might have caused death if it were more severe.
- requires inpatient hospitalisation or prolongation of existing hospitalisation,
- results in persistent or significant disability/incapacity, or
- is a congenital anomaly/birth defect.

Unexpected and expected SAEs will be manged in the same manner. Hospitalization for either elective surgery related to a pre-existing condition, which did not increase in severity, or frequency following initiation of the study, or for routine clinical procedures (including

hospitalization for "social" reasons) are not considered as SAEs. Medical judgment should be exercised in deciding whether an adverse event/reaction is serious in other situations. Important adverse events/reactions that are not immediately life-threatening or do not result in death or hospitalization but may jeopardize the participant or may require intervention to prevent one of the other outcomes listed in the definition above, should also be considered serious. These are considered “Other Important Medical Events”. SAEs are subject to expedited reporting to the sponsor, ethics committee and local safety monitor (s) as described in the section below.

Suspected Unexpected Serious Adverse Reaction (SUSAR)

An adverse reaction, the nature or severity of which is not anticipated based on the applicable product information (*e.g.*, Investigator's Brochure for an unapproved investigational medicinal product) is considered as an unexpected adverse drug reaction. Where the adverse reaction is also considered to have a possible, probable or definite relationship with the vaccine, and meets the criteria for a serious adverse reaction, it is termed a Suspected Unexpected Serious Adverse Reaction (SUSAR). These events are subject to expedited reporting as for SAEs and are also reported to the regulatory authority (see below).

Severity assessment

The severity of clinical and laboratory adverse events and their relationship to the investigational product will be assessed according to the scales in Tables 7 to 10 below.

Table 7: **Severity grading criteria for local adverse events*** erythema ≤2.5cm is an expected consequence of skin puncture and will therefore not be considered an adverse event

Adverse Event	Grade	Intensity
Pain at injection site	1	Pain that is easily tolerated
	2	Pain that interferes with daily activity
	3	Pain that prevents daily activity

	4	Emergency room visit or hospitalization
Tenderness	1	Mild discomfort to touch
	2	Discomfort with movement
	3	Significant discomfort at rest
	4	Emergency room visit or hospitalization
Erythema at injection site*	1	2.5-5cm
	2	5.1-10cm
	3	>10cm
	4	Necrosis of exfoliative dermatitis
Induration swelling at injection site	1	2.5-5cm and does not interfere with activity
	2	5.1-10cm or interferes with activity
	3	>10cm or prevents daily activity
	4	Necrosis

Table 8: Severity grading criteria for physical observations

	Grade 1 (mild)	Grade 2 (moderate)	Grade 3 (severe)	Grade 4 Potentially Life threatening
Fever (axillary)	38.0°C - 38.4°C	38.5°C – 38.9°C	39.0°C - 40°C	>40°C
Tachycardia (bpm)*	101 - 115	116 – 130	>130	Emergency room visit or hospitalization for arrythmia

Bradycardia (bpm)**	50 – 54	45 – 49	<45	Emergency room visit or hospitalization
Systolic hypertension (mmHg)	141 - 150	151 – 155	≥155	Emergency room visit for malignant hypertension
Systolic hypotension (mmHg)***	85 - 89	80 – 84	<80	Emergency room visit or hospitalization for hypotensive shock
Diastolic hypertension (mmHg)	91 - 95	96 – 100	≥100	Emergency room visit or hospitalization for malignant hypertension
Respiratory Rate breaths per minute	17 - 20	21- 25	>25	Intubation

*Taken after ≥10 minutes at rest

**When resting hear rate is between 60 – 100 beats per minute Use clinical judgement when characterising bradycardia among some healthy subject populations, for example, conditioned athletes.

***Only if symptomatic (e.g. dizzy/ light-headed)

Table 9: Severity grading criteria for local and systemic AEs

**GRADE 0**	None: Symptom not experienced
**GRADE 1**	Mild: Transient or mild discomfort (< 48 hours); No interference with activity; No medical intervention/therapy
**GRADE 2**	Moderate: Mild to moderate limitation in activity – some assistance may be needed; no or minimal medical intervention/therapy required
**GRADE 3**	Severe: Marked limitation in activity, some assistance usually required; medical intervention/therapy required.
**Grade 4**	Potentially Life-threatening; requires assessment in Emergency room visit or hospitalisation.

Table 10: Relationship to Investigational Product

0	**No Relationship**	No temporal relationship to study product ***and***<br>Alternate aetiology (clinical state, environmental or other interventions); ***and***<br>Does not follow known pattern of response to study product
1	**Unlikely**	Unlikely temporal relationship to study product ***and***<br>Alternate aetiology likely (clinical state, environmental or other interventions) ***and***<br>Does not follow known typical or plausible pattern of response to study product.
2	**Possible**	Reasonable temporal relationship to study product; ***or***<br>Event not readily produced by clinical state, environmental or other interventions; ***or***<br>Similar pattern of response to that seen with other vaccines
3	**Probable**	Reasonable temporal relationship to study product; ***and***<br>Event not readily produced by clinical state, environment, or other interventions ***or***<br>Known pattern of response seen with other vaccines
4	**Definite**	Reasonable temporal relationship to study product; ***and***<br>Event not readily produced by clinical state, environment, or other interventions; ***and***<br>Known pattern of response seen with other vaccines

10.3. Documenting AEs

Both solicited and unsolicited AEs will be recorded on the participant's diary card/e-diary. The diagnosis, date and time of onset, outcome, severity and relationship to vaccination will be established. Details of any treatment or concomitant interventions will be recorded. Only SAEs will be recorded for participants who are unblinded and receive an approved/licensed COVID-19 vaccine as part of national roll-out strategy. All AEs that result in a volunteer's withdrawal from the study will be followed up until a satisfactory resolution occurs (if the volunteer consents to this), or until a non-study related causality is assigned.

10.4. Reporting Serious Adverse Events (SAEs) and/or Unexpected AEs

The PI/designee will be responsible for reporting and providing updates of SAEs/SUSARs to the Sponsor, IRB, DSMB, Local Safety Monitor (LSM), and regulatory authorities. Every

SAE occurring throughout the trial must be reported by telephone, e-mail or fax to the Sponsor , DSMB Chair, and PPB within twenty-four hours, even if the investigator considers the SAE not related to vaccination. Initial notifications to PPB will be submitted through the online portal. The DSMB may ask for the study to be stopped, or for an extended study hold to be applied while further data and information are sought. The DSMB will make its recommendation to the Sponsor, who will have ultimate responsibility for acting on the recommendation. The PI will then complete a hard copy SERU SAE report as soon as possible and within 5 working days or 7 calendar days. Any relevant information concerning the adverse event that becomes available after the SAE report form has been sent (outcome, precise description of medical history, results of the investigation, copy of hospitalisation report, etc.) will be forwarded to the Sponsor in a timely manner, the anonymity of the subjects shall be respected and data protection policies adhered to when forwarding this information.

Grade 4 laboratory AEs should be reported as SAEs under the category of an outcome of an important medical event. Emergency room attendance should not routinely be reported as SAEs unless they meet the SAE definition described above.

Restrictions

Eosinophilia as a marker skewed Th2 responses will be routinely monitored in participants attending their COVID-19 testing and follow-up visits. Marked eosinophilia of $\geq 1.5 \times 10^9$/L will be reported as an SAE.

Cases falling under the Hy's Law should be reported as SAEs. A Hy's Law Case is defined by FDA Guidance for Industry "Drug-Induced Liver Injury: Premarketing Clinical Evaluation" (2009). Any study participant with an increase in Aspartate Aminotransferase (AST) or Alanine Aminotransferase (ALT) ≥ 3x Upper Limit of Normal (ULN) together with Total Bilirubin ≥2xULN, where no other reason can be found to explain the combination of these abnormal results, e.g., elevated serum alkaline phosphatase (ALP) indicating cholestasis, viral hepatitis A, B or C, another drug capable of causing the observed injury, amongst others.

Any study related SUSAR or serious adverse event related to participation in the study must be reported by telephone, e-mail or fax to the Sponsor and DSMB within twenty-four hours, and to SERU via email within 48 hours of the PI being aware of the event. The hard copies of

the report must be forwarded to the SERU secretariat within 5 working days of the initial notification. Follow-up reports should be submitted as soon as more information becomes available. Initial reports of SUSARs will be provided by the Sponsor to PPB as soon as possible but within seven (7) calendar days of the notification of the SUSARs with follow up reports being provided within a further eight (8) calendar days. The SUSAR and SAE reports will be submitted to PPB through the online system at https://ctr.pharmacyboardkenya.org/users/login. A summary of SAEs and SUSARs shall be submitted every six months from the day of approval of the study. In the UK, Brazil and South Africa trials SAEs are reported to Chief Investigator within 24 hours of becoming aware. Chair of DSMB will be notified of any SAE possibly, probably, definitely related to study intervention within 24hours of investigator aware of occurrence. All SUSARS are reported by the sponsor delegate to relevant authorities; fatal or life-threatening SUSARS are reported no later than 7 calendar days, with a further 8 days for additional information from initial report. All other SUSARS are reported within 15 calendar days.

10.5. Emergency Procedures

Standard resuscitation equipment will be readily available at the vaccination clinic including bag mask valve, oxygen cylinder, non-rebreather bag and adrenaline.

10.6. Pregnancy

Pregnancy will not be considered as an adverse event but will be reported to the Sponsor, IRB and regulatory authority within 24 hours of the PI becoming aware. Subsequently, this will be updated on the pregnancy reporting CRF. Any pregnancy will be followed to term, and any premature terminations reported. Further, the investigators will assess the health of the mother and baby after delivery. At a minimum, pregnant participants will be seen at every antenatal visit then within 72 hours of delivery for a post-natal check. A final assessment will be conducted 6 weeks post-natally and further follow-up offered if warranted. All assessments of the infant will be conducted by a paediatrician. This will allow the team to report any SAE data related to congenital anomalies or birth defects.

10.7. Procedures for reporting any protocol violation(s)

Protocol violations will be reported to the sponsor, regulatory and ethics committees as specified in their guidelines.

10.8. Study Governance, LSM and DSMB

10.8.1. Local Safety Monitor

The Local Safety Monitor will be a clinician resident in Kilifi (therefore likely to be linked to or a staff member of KEMRI CGMRC) but independent of the study team. The LSM will act as a semi-independent assessor of participants experiencing important safety events at the request of the DSMB and will provide their observations to the DSMB and/or local clinicians or study team members where appropriate. They will not be responsible for safety decisions pertaining to trial progression.

10.8.2. Data and Safety Monitoring Board

The DSMB will include at least 3 independent members (at least 1 clinician and 1 statistician), including internationally reputable clinical and/or statistical experts, and will have access to all relevant data on administration of the vaccine. The DSMB will include at least 1 member with expert knowledge of the Kenyan context. The DSMB will be convened at the start of the study before vaccinations begin to review the protocol and their responsibilities. A local safety monitor will also be appointed at this time to provide independent safety assessments of participants. The DSMB will review; a) all SAEs/ SUSARs as they occur, b) severe adverse events reported within a week of their occurrence with an updated summary of all severe adverse events reported to date. For interim safety assessments, the following data will be provided systematically; a) all SUSARS/SAEs to date, b) all severe adverse events to date. In addition, any other events that the investigators or other stakeholders are concerned about will be provided, and any event that the LSM considers relevant and asks about. Scheduled DSMB meetings will include, one before the start of the study, and two-weekly safety reviews for the first four weeks of the phase Ib trial and then subsequently at a frequency to be agreed between the trial investigators and the DSMB as the trial progresses. The DSMB will provide approval for enrolment of participants in phase II after review of safety data accrued up to day 28 post-vaccination of the participants in groups 1a and 2a (Table 3a), and a final scheduled meeting will be held at the end of the study. The DSMB will also have ad-hoc meetings should there be any safety

concerns and may increase the frequency of meetings if there are concerns from other studies where the ChAdOx1 nCov-19 vaccine is being evaluated.

LABORATORY INVESTIGATIONS

Vaccine immunogenicity: this will be assessed by a variety of immunological assays. These will include measurement of antibodies to SARS-CoV-2 antigens, including Spike protein, by ELISA and virus neutralising assays, ex vivo ELISpot assays for interferon gamma and flow cytometry assays, functional antibody assays and B cell analyses. Other exploratory immunological assays including cytokine and chemokine analysis and antibody assays on nasopharyngeal swabs, DNA analysis of genetic polymorphisms potentially relevant to vaccine immunogenicity and gene expression studies amongst others may be performed at the discretion of the Investigators. Venous blood will be collected as indicated in Tables 4 and processed for serum and peripheral blood mononuclear cells (PBMCs) within 6 hours of sample collection at the KEMRI CGMRC immunology lab using local SOPs. Many of these immunological assays are in development through ongoing collaborations between KEMRI CGMRC and the University of Oxford, with assay development efforts including participation in WHO-led standardization of serological methods with other labs globally.

All immunology work for this trial will be led from Kenya but SOPs for the serology and cellular immunology assays to be used will be harmonised between KEMRI CGMRC and the University of Oxford, so that similar validated immunological methods and endpoints will be used for the trial in Kenya and other sites (i.e. UK, Brazil, South Africa). This may involve shipment of serum or plasma and/or PBMCs to collaborating laboratories in the University of Oxford or other specialist laboratory, but these samples would remain anonymised. Informed consent for sample shipment will be obtained from volunteers and a Material Transfer Agreement will be developed, and approval obtained from SERU before samples are transferred. Approval will be sought from SERU if shipment of samples to other specialist laboratories is necessary.

Participants will be informed that there may be leftover samples of their blood (after all testing for this study is completed), and that such samples may be stored in the KWTRP Biobank for possible future research (exploratory immunology), including human DNA and RNA analyses to search for correlates of vaccine immunogenicity and efficacy. New research

questions unrelated to the objectives of this protocol will require a new protocol for review by SERU.

Vaccine efficacy: ChAdOx1 nCoV-19 efficacy against COVID-19 and asymptomatic SARS-CoV-2 carriage will be assessed in comparison to the control rabies vaccine as a secondary objective. Passive detection of COVID-19 cases among all study participants will be done throughout the study duration. Nasopharyngeal and throat swabs will be taken following local SOPs from any study participant with symptoms consistent with COVID-19. These will be tested using validated quantitative real-time PCR assays (RT-PCR) for SARS-CoV-2 currently in use at KEMRI CGMRC, the nationally designated COVID-19 testing centre in coastal Kenya. Sampling and clinical management of participants will be facilitated by MoH as per prevailing national guidelines. Symptomatic participants may be regularly reviewed over the phone or via video call if clinically appropriate. Face-to-face reviews will only be done if they adhere to the prevailing government directives, and a risk assessment will be done to determine the most appropriate location for this review. The RT-PCR assay will also be used to monitor asymptomatic carriage of SARS-CoV-2 in nasopharyngeal/throat swabs collected from study participants during the scheduled clinic visits as outlined in Table 4 and RT-PCR positive results fed back through the public health reporting mechanisms already in place at KEMRI CGMRC. RT-PCR positive samples will be whole genome sequenced, directly from clinical samples or after viral isolation in tissue culture (in the KEMRI CGMRC biosafety level 3 lab), and any viral genetic variability correlated with the vaccine induced immune response. These isolated viruses may be used for testing neutralising antibodies in sera from study participants at the KEMRI CGMRC biosafety level 3 lab. The relationship between COVID-19 and cytokine and chemokine responses, immune responses to other pathogens (including other coronaviruses, arboviruses, and malaria) at baseline and during follow-up, and other host factors will be determined to identify correlates and confounders of protective immunity against COVID-19. Clinical data and convalescent blood and nasopharyngeal/throat samples for immunology purposes will be taken from day 14 post-COVID-19 diagnosis or after discharge from care. This sampling will be done by MoH staff or the study team depending on the prevailing government directives.

12. STATISTICS

12.1. Determination of sample size

The phase Ib trial will be a descriptive safety study, where volunteers will be vaccinated with two doses of $5x10^{10}$ vp ChAdOx1 nCoV-19 (n=20) or two doses of control rabies vaccine (n=20) (see Table 3a and 3b). This sample size should allow an estimation to be made of the frequency and magnitude of adverse events following vaccination, rather than aiming to obtain statistical significance for safety differences between individuals. Safety data will be presented according to frequency, severity, relatedness to vaccine and duration of adverse events. The primary endpoint for the phase II trial (n=180 per group) will be vaccine immunogenicity (seroconversion) as measured by IgG ELISA against SARS-CoV-2 spike protein 28 days after the second dose of ChAdOx1 nCoV-19 vaccine. A sample size of 200 per vaccine group (combined phase Ib and phase II) will allow detection of at least 70% seroconversion with <5% error margin.

12.2. Statistical and analytical plans

12.2.1. Data management

A Data Management team will be located at KEMRI CGMRC with data collection using eCRFs/CRFs by designated staff. Clinician/data entry clerks/trained study team members will use password-protected computers. Data will be entered onto an electronic database. This will be done via secure web interface with data checks used during data entry to ensure data quality. The database will be activated for the study only after successfully passing a formal design and test procedure. Laptops and desktop computers will be used for data entry. Management and maintenance of computers will lie with the operational support and data managers at the study site.

12.2.2. Data security, access and back up

The database will be kept in a locked server-room. Only the system administrators have direct access to the server and back-up tapes. A role concept with personal passwords (site investigator, statistician, monitor, administrator etc.) regulates permission for each user to use the system and database, as he/she requires.

All data entered onto the eCRF/CRFs are transferred to the database using Secure Sockets Layer (SSL) encryption. Each data point has attributes attached to it identifying the user who entered it with the exact time and date. Retrospective alterations of data in the database are

recorded in an audit table. Time, table, data field and altered value, and the person are recorded (audit trail). A multi-level back-up will be implemented. Back-ups of the entire system including the database are run internally several times per day and on external tapes once a day. The back-up tapes are stored in a secure location.

12.2.3. Analysis of study endpoints

Adverse events occurring during the study follow up period will be analysed and compared between the ChAdOx1 nCoV-19 and rabies vaccine groups. This will be a descriptive analysis and will include all AEs up to 28 days post-vaccination, and SAEs that occurred any time during study follow-up.

Information for each of the following categories will be presented for the phase Ib and phase II studies: number of individuals screened for eligibility; the number and reason of screen failures; the number and percentage of eligible individuals who consent and are randomized; the number and percentage of randomized individuals who receive a vaccine; the number and percentage of vaccinated individuals who complete the scheduled post-vaccination visits; and the number of individuals who discontinue and the reason for discontinuation. The influence of the various demographic factors (e.g. gender, age) on vaccine safety and immunogenicity will be assessed.

The intention to treat (ITT) population will comprise all randomized participants who received a study vaccine and that have at least one post-vaccination blood sample. The per protocol (PP) population will include randomized participants who have a blood sample at baseline and 28 days (+ 3 days) post-vaccination, and for whom the eligibility criteria were correctly applied. The safety population will include all subjects who received a study vaccine.

The primary analysis for the phase II trial (vaccine immunogenicity) will be a Chi2 test of proportions of vaccinees in each group seroconverting against SARS-CoV-2 spike protein at day 28 post-booster vaccination as measured by IgG ELISA. Repeated measures non-parametric tests will be used to compare immune responses measured at various times during follow up between vaccine groups (see Table 4). These will include IgG endpoint geometric mean titres (GMT), IFNg ELISpot responses, virus neutralising antibody titres, among others. This unblinded safety and immunogenicity analysis will be done when all phase Ib and phase

II participants have peak immunogenicity data (at ≥28 days post-booster vaccination), and again at the end of the study. Vaccine availability will continue to increase exponentially making unblinding inevitable over the course of the study (see section 9.11 for unblinding procedures). These data will continue to inform the vaccine programme in Kenya and elsewhere on the continent.

Vaccine efficacy will be a secondary objective. Survival analysis, using Cox regression models, will be used to estimate vaccine efficacy against virologically confirmed COVID-19. Only events that occur more than 14 days after the booster vaccination dose will be included in efficacy evaluations to allow time for the vaccine recipients to mount a protective immune response and to provide a more accurate estimation of vaccine efficacy. Vaccine immunogenicity and efficacy analysis will censor participants at the point of testing positive for COVID-19 or SARS-CoV-2 carriage. Secondary exploratory analysis will be done to investigate boosting of immune response following infection, and the frequency of re-infections.

A detailed statistical analysis plan (SAP) will be provided separately and finalized after the study has started (and before the database lock). This SAP will include all conventions on data, descriptive and statistical analyses to be performed on collected data during the conduct of the study.

13. DIRECT ACCESS TO SOURCE DATA/DOCUMENTS

The PI will provide direct access to the source data documents to the ethics committee should this be requested, to the regulatory agency, and to authorized representatives of the Sponsor in order to permit trial related monitoring and audits.

14. QUALITY CONTROL AND QUALITY ASSURANCE

The Clinical Trials Facility (CTF) monitors will conduct monitoring visits as per the risk-based monitoring plan at the trial site will to check that the trial is being conducted, data recorded, analysed and accurately reported according to the protocol, trial SOPs and in compliance with ICH GCP. The laboratory QA manager will conduct audits which include laboratory activities

according to an agreed audit schedule. The internal audits will supplement the monitoring process. On the recommendation of the sponsor the Clinical Trials and Research Governance Office in Oxford can carry out an audit to ensure compliance with the protocol, GCP and appropriate regulations but this responsibility is primarily that of the CTF monitors.

15. INTELLECTUAL PROPERTY

Any intellectual property rights that arise from the work will be safeguarded according to the current KEMRI guidelines and the Industrial Property Act of 2001, sections 32, 58 and 80. The scientific and intellectual contributions of all persons involved in the research will be appropriately acknowledged in all publications and presentations arising from the work. The University of Oxford and AstraZeneca developed an agreement in April 2020. AstraZeneca is working with global partners on the international distribution of the vaccine, particularly working to make it available and accessible for low- and medium-income countries. Both AstraZeneca and the University of Oxford have agreed to operate on a not-for-profit basis for the duration of the coronavirus pandemic, with only the costs of production and distribution being covered.

Oxford University and its spin-out company Vaccitech, who jointly have the rights to the platform technology used to develop the vaccine candidate, will receive no royalties from the vaccine during the pandemic. AstraZeneca has also recently completed agreements with the UK, US, the Coalition for Epidemic Preparedness Innovations and Gavi the Vaccine Alliance for 700 million doses, and it agreed a licence with the Serum Institute of India for the supply of an additional one billion doses, principally for low-and middle-income countries. Total manufacturing capacity currently stands at two billion doses which is intended to ensure manufacturing capacity for LMICs. As per current agreements responsibility for distribution of the vaccine if proved efficacious remains with AstraZeneca**.**

The control group will be offered the ChAdOx1 nCoV-19 vaccine at the end of the trial. If the vaccine is licensed at this point, then this will be done as a non-research procedure (or after a short delay if licensing is under consideration). If the vaccine is still not licensed, then the investigators will write to SERU, OxTREC and PPB to ask their input on the appropriateness of vaccination of the control group based on all available global data on safety and efficacy. Use of the unlicensed vaccine will require a protocol amendment in order to allow vaccination to take place as a research procedure. The required monitoring, follow up and safety

assessments after vaccination will depend on the data available from the trial and from other related trials (e.g. UK, Brazil and South Africa), and will be specified in full in the amendment.

16. TIME FRAME/DURATION OF THE TRIAL

We anticipate the study will take 2 years including data analysis and write-up.

17. ETHICS

Ethical approval will be sought from KEMRI Scientific & Ethics Review Unit (SERU), OxTREC and any other applicable IRBs according to local regulation. Regulatory approval will be sought from the Pharmacy and Poisons Board (PPB). The trial will also be registered with NACOSTI (National Commission for Science, Technology and Innovation).

17.1. Human Subjects

In all investigations involving human subjects, the following guidelines should be observed:

i. “First, do no harm.”

This trial will be conducted in accordance with the current Declaration of Helsinki as agreed by the World Medical Association General Assembly, ICH Good Clinical Practice and local regulatory requirements. The safety of the participants will be monitored throughout the trial and any AEs or SAE reported will be treated immediately.

ii. **Ethical review**

Before the inclusion of the first subject in the study, the protocol and the informed consent must be approved by SERU, OxTREC and PPB. The participant will give written informed consent before being included in the trial, after having been informed of the nature of the trial, the potential risks and their obligations. Informed consent forms will be provided in duplicate (original kept by the PI, one copy kept by the participant or the participant’s legally acceptable representative).

iii. Confidentiality

Personal information of the participants, HIV testing and referral information will be handled confidentially in keeping with our data protection policies, GDPR (General Data Protection Regulation) and Kenyan law. They will be linked to personal identifying information only on documents held securely in the Clinical Trials Facility/KEMRI-CGMRC site/clinic-sites, and no personal information will be entered into the electronic database. This will identify subjects by their unique code number only. Immunology bloods and nasopharyngeal swabs will not have personal identifying information. Any future research related to the data or samples from this study will require written approval from SERU before it can be done.

17.2. Public, Community and Stakeholder Engagement

A detailed engagement plan has been developed for this study. The plan outlines communication messages, and key stakeholders from National, County, and community levels, who must be engaged prior to and during the course of study implementation. The KEMRI CGMRC Communications and Engagement team and the investigators will form a Community engagement Advice for Studies (CAST) group whose role will be to coordinate implementation of the activities outlined in this plan. Stakeholder and Community/Public engagement will be done in accordance with the prevailing Kenya government advisories at the time of recruitment. Prior to regulatory approval, engagement will be done with top level government officials in the Ministry of Health as well as Office of the President.

On receipt of approval from the SERU, OxTREC, PPB and Kilifi and Mombasa County Departments of Health, we will plan sensitization meetings with the County, Sub-County Health Management and hospital management teams. Concurrent meetings will be held with medical associations where the potential study participants may be members. Engagement meetings will be held with HCWs in their areas of work through meetings or CMEs, or other available platforms. Engagement activities will also be conducted with political, community and religious leaders, and community representatives to explain the study and its aims, and to discuss and respond to concerns.

In the course of study implementation, broader publics, including the media will be engaged as well. Innovative modes of meeting, including virtual/teleconferences, films/videos, will be used to comply with existing government physical distancing directives. Community

engagement will continue throughout the study period, collecting and responding to concerns from the community about the study through the CLG team.

17.3. Compensation and Reimbursement

We will reimburse participants for travel required to attend screening and to attend for vaccinations and follow up. Compensation for study-related out of pocket expenses will be done in accordance with standard figures determined by KEMRI CGMRC based on government recommendations on daily wages for skilled and non-skilled labour in Kenya. Compensation will be KSH2,000 for out of pocket expenses plus additional transport reimbursement based on public transport costs for distance travelled to attend clinic. The study will also cover costs for additional contraception required throughout the course of the study for female adults in cases where they are unable to access free services. Reasonable costs for data bundles used for e-Consent as well as for meals when not fully covered during isolation and quarantine will also be reimbursed.

17.4. Patient Data Protection/Confidentiality

Clinical records will be kept in locked cabinets at the KEMRI-CGMRC site or clinic sites. All immunological and SARS-CoV-2 PCR data will be kept in anonymized databases linked by the study number to clinical data. The data will be stored in password protected computers and the hard copy documents will be stored in lockable cabinets. Participant identifiable information will not be shared in any way that is not necessary for the day-to-day administration of the trial. Participant identifiable information will not be published. History taking and examination for participants will be carried out with the normal respect towards privacy, dignity and confidentiality. Clinical written information arising from such episodes will be held securely, as normal patient confidentiality guidelines dictate.

17.5. Data Sharing

The study is a collaboration between KEMRI CGMRC and the University of Oxford. Individual-level anonymized data will be shared with the Sponsor. For wider stakeholder engagement and the medical community, summary-level statistical analyses will be shared. Information collected or generated during this study may be anonymised for use to support new research on coronavirus vaccines and immunology. Any future research using

information from this study must first be approved by the Centre Scientific Committee at KEMRI-CGMRC and the KEMRI Scientific and Ethics Review Unit to make sure that the interests of participants and their communities are protected. Data will be managed by KEMRI CGMRC.

17.6. Safety

The study team will provide medical care to participants during the study follow-up period for acute illnesses managed in government facilities. The study team will not become responsible for long-standing chronic conditions that were present before vaccination, or those that are unrelated to vaccination. Medical care will be provided within Kenyan Ministry of Health Guidelines.

18. ARCHIVING AND RECORD RETENTION

The investigators will maintain appropriate medical and research records for this study, in compliance with ICH E6 GCP, GDPR, regulatory and institutional requirements for the protection of confidentiality of participants. The PI, co-investigators, and clinical research staff will have access to records. The investigators will permit authorized representatives of the Sponsor, and regulatory agencies to examine (and when required by applicable law, to copy) clinical records for the purposes of quality assurance reviews, audits and evaluation of the study safety and progress. The Trial Master File (TMF), containing all documents and information relating to the trial, will be maintained at KEMRI CGMRC.

The TMF including a copy of the final completed CRFs, as well as all source documentation will be retained by the PI and one copy will be maintained by the Sponsor, who will ensure that it is stored with other study documents, such as the signed informed consent forms, protocol, the investigator’s brochure and any protocol amendments, in a secure place following local regulations. The final study database will be securely kept with all archive tables for at least 10 years. Paper CRFs and study files at KEMRI CGMRC will be archived following local laws.

19. TRIAL MANAGEMENT

A trial management group will be convened for coordination of study activities and will include investigators from KEMRI CGMRC, MoH and University of Oxford. The trial will be registered by the University of Oxford through the Pan African Clinical Trials Registry (https://pactr.samrc.ac.za) as required by PPB. The University of Oxford and KEMRI CGMRC will be responsible for initiating, registering and conduct of the trial, and as such, will be involved in the study design, collection, management and analysis, and interpretation of data, and writing of the report. The Sponsor will ensure that the trial is monitored properly, and results made available. The Principal Investigator will be responsible for reporting of SAEs to the DSMB and sponsor within 24 hours of discovery of the SAE. The investigators will be responsible for reporting a summary of safety data at the end of the trial to PPB and will report SUSARs and SAEs deemed causally related to the study vaccine to SERU and PPB. Regular monitoring will be performed according to International Council for Harmonisation (ICH) Good Clinical Practice (GCP) and a Monitoring Plan. Monitors will check whether the clinical trial is conducted, and data are generated, documented and reported in compliance with the protocol, GCP and the applicable regulatory requirements. The site team led by the PI will be responsible for local submissions to the regulators and all the staff will have good clinical practice training prior to study start.

20. REPORTING, DISSEMINATION AND NOTIFICATION OF RESULTS

Results will be published in an open-access journal. Anonymized data will be made available with these publications. We will feedback individual results with clinical relevance to participants in real-time. Results will be shared with stakeholders who were sensitized such as sub-county health management teams, professional associations and recruiting

hospitals/workplaces. Furthermore safety, immunogenicity and efficacy data will be shared with trial participants as it becomes available during the conduct of the trial.
Summaries of the outcomes of the trial will be provided during community meetings in the areas from which participants are recruited. It is not anticipated that substantial information in this form will be available until at least the second year of the trial, and this will be made clear during initial meetings to avoid unrealistic expectations regarding the rapidly of feedback.

21. REFERENCES

1. Chen WH, Strych U, Hotez PJ, Bottazzi ME. The SARS-CoV-2 Vaccine Pipeline: an Overview. Curr Trop Med Reports. 2020;1–4.
2. Wong J, Goh QY, Tan Z, Lie SA, Tay YC, Ng SY, et al. Preparing for a COVID-19 pandemic: a review of operating room outbreak response measures in a large tertiary hospital in SingaporeSe préparer pour la pandémie de COVID-19: revue des moyens déployés dans un bloc opératoire d’un grand hôpital tertiaire au S. Can J Anesth Can d’anesthésie [Internet]. 2020; Available from: https://doi.org/10.1007/s12630-020-01620-9
3. Lai J, Ma S, Wang Y, Cai Z, Hu J, Wei N, et al. Factors Associated With Mental Health Outcomes Among Health Care Workers Exposed to Coronavirus Disease 2019. JAMA Netw open [Internet]. 2020;3(3):e203976. Available from: http://www.ncbi.nlm.nih.gov/pubmed/32202646
4. Singhal T. A Review of Coronavirus Disease-2019 (COVID-19). Indian J Pediatr. 2020;87(April):281–6.
5. CDC. What you need to know about coronavirus disease 2019 (COVID-19) [Internet]. Vol. 2019. 2020. p. 314937. Available from: https://www.cdc.gov/coronavirus/2019-ncov/downloads/2019-ncov-factsheet.pdf
6. Li Q, Guan X, Wu P, Wang X, Tong Y, Ren R, et al. Early Transmission Dynamics in Wuhan, China, of Novel Coronavirus–Infected Pneumonia. N Engl J Med. 2020;382(13):1199–207.
7. WHO. Clinical management of severe acute respiratory infection (SARI) when COVID-19 disease is suspected . [Internet]. 2020. p. 1–21. Available from: https://www.who.int/publications-detail/clinical-management-of-severe-acute-

respiratory-infection-when-novel-coronavirus-(ncov)-infection-is-suspected

8. Mackenzie JS, Smith DW. COVID-19 : a novel zoonotic disease caused by a coronavirus from China : what we know and what we don ' t. Microbiol Aust. 2020;10(171):A-F.
9. Adhikari SP, Meng S, Wu Y-J, Mao Y-P, Ye R-X, Wang Q-Z, et al. Epidemiology, causes, clinical manifestation and diagnosis, prevention and control of coronavirus disease (COVID-19) during the early outbreak period: a scoping review. Infect Dis poverty [Internet]. 2020;9(1):29. Available from: http://www.ncbi.nlm.nih.gov/pubmed/32183901
10. Dhama K, Sharun K, Tiwari R, Dadar M, Singh Y. COVID-19 , an emerging coronavirus infection : advances and prospects in designing and developing vaccines , immunotherapeutics , and therapeutics. Hum Vaccin Immunother [Internet]. 2020;00(00):1–7. Available from: https://doi.org/10.1080/21645515.2020.1735227
11. Li F. Structure, Function, and Evolution of Coronavirus Spike Proteins. Annu Rev Virol. 2016;3(1):237–261.
12. Alharbi KN, Padron-regalado E, Thompson CP, Kupke A, Wells D, Sloan MA, et al. ChAdOx1 and MVA based vaccine candidates against MERS-CoV elicit neutralising antibodies and cellular immune responses in mice. Vaccine. 2017;35(January):3780–3788.
13. Doremalen N Van, Lambe T, Spencer A, Belij-Rammerstorfer S, Purushotham JN, Port JR, et al. ChAdOx1 nCoV-19 vaccination prevents SARS-CoV-2 pneumonia in rhesus macaques. bioRxiv Prepr Serv Biol. 2020;1–7.
14. Weingartl H, Czub M, Czub S, Neufeld J, Marszal P, Gren J, et al. Immunization with Modified Vaccinia Virus Ankara-Based Recombinant Vaccine against Severe Acute Respiratory Syndrome Is Associated with Enhanced Hepatitis in Ferrets. J Virol. 2004;78(22):12672–6.
15. Liu L, Wei Q, Lin Q, Fang J, Wang H, Kwok H, et al. Anti-spike IgG causes severe acute lung injury by skewing macrophage responses during acute SARS-CoV infection. JCI insight [Internet]. 2019;4(4):1–19. Available from: https://doi.org/10.1172/jci. insight.123158.
16. Tseng C Te, Sbrana E, Iwata-Yoshikawa N, Newman PC, Garron T, Atmar RL, et al. Immunization with SARS coronavirus vaccines leads to pulmonary immunopathology

on challenge with the SARS virus. PLoS One. 2012;7(4).

17. Agrawal AS, Tao X, Algaissi A, Garron T, Narayanan K, Peng BH, et al. Immunization with inactivated Middle East Respiratory Syndrome coronavirus vaccine leads to lung immunopathology on challenge with live virus. Hum Vaccines Immunother [Internet]. 2016;12(9):2351–6. Available from: http://dx.doi.org/10.1080/21645515.2016.1177688
18. Van Doremalen N, Haddock E, Feldmann F, Meade-White K, Bushmaker T, Fischer R, et al. A single dose of ChAdOx1 MERS provides broad protective immunity against a variety of MERS-CoV strains. bioRxiv [Internet]. 2020;2020.04.13.036293. Available from: http://biorxiv.org/content/early/2020/04/13/2020.04.13.036293.abstract
19. Munster VJ, Wells D, Lambe T, Wright D, Fischer RJ, Bushmaker T, et al. Protective efficacy of a novel simian adenovirus vaccine against lethal MERS-CoV challenge in a transgenic human DPP4 mouse model. npj Vaccines [Internet]. 2017;2(1):1–3. Available from: http://dx.doi.org/10.1038/s41541-017-0029-1
20. Alharbi NK, Qasim I, Almasoud A, Aljami HA, Alenazi MW, Alhafufi A, et al. Humoral Immunogenicity and Efficacy of a Single Dose of ChAdOx1 MERS Vaccine Candidate in Dromedary Camels. Sci Rep [Internet]. 2019;9(1):1–11. Available from: https://doi.org/10.1038/s41598-019-52730-4
21. Ogwang C, Kimani D, Edwards NJ, Roberts R, Mwacharo J, Bowyer G, et al. Prime-boost vaccination with chimpanzee adenovirus and modified vaccinia Ankara encoding TRAP provides partial protection against Plasmodium falciparum infection in Kenyan adults. Sci Transl Med. 2015;7(286):1–20.
22. Ogwang C, Afolabi M, Kimani D, Jagne YJ, Sheehy SH, Bliss CM, et al. Safety and Immunogenicity of Heterologous Prime-Boost Immunisation with Plasmodium falciparum Malaria Candidate Vaccines, ChAd63 ME-TRAP and MVA ME-TRAP, in Healthy Gambian and Kenyan Adults. PLoS One. 2013;8(3):1–11.
23. Folegatti PM, Bittaye M, Flaxman A, Lopez FR, Bellamy D, Kupke A, et al. Safety and immunogenicity of a candidate Middle East respiratory syndrome coronavirus viral-vectored vaccine: a dose-escalation, open-label, non-randomised, uncontrolled, phase 1 trial. Lancet Infect Dis [Internet]. 2020;20(30160–2):1–11. Available from: https://linkinghub.elsevier.com/retrieve/pii/S1473309920301602

24. Antrobus RD, Coughlan L, Berthoud TK, Dicks MD, Hill AVS, Lambe T, et al. Clinical assessment of a novel recombinant simian adenovirus ChAdOx1 as a vectored vaccine expressing conserved influenza a antigens. Mol Ther. 2014;22(3):668–74.
25. Coughlan L, Sridhar S, Payne R, Edmans M, Milicic A, Venkatraman N, et al. Corrigendum to “Heterologous Two-dose Vaccination with Simian Adenovirus and Poxvirus Vectors Elicits Long-lasting Cellular Immunity to Influenza Virus A in Healthy Adults” [EBioMedicine 29 (2018) 146–154] (S2352396418300653) (10.1016/j.ebiom.2018.02.011). EBioMedicine. 2018;31:321.
26. Coughlan L, Sridhar S, Payne R, Edmans M, Milicic A, Venkatraman N, et al. Heterologous Two-Dose Vaccination with Simian Adenovirus and Poxvirus Vectors Elicits Long-Lasting Cellular Immunity to Influenza Virus A in Healthy Adults. EBioMedicine. 2018;29:146–54.
27. Wilkie M, Satti I, Minhinnick A, Harris S, Riste M, Ramon RL, et al. A phase I trial evaluating the safety and immunogenicity of a candidate tuberculosis vaccination regimen, ChAdOx1 85A prime – MVA85A boost in healthy UK adults. Vaccine [Internet]. 2020;38(4):779–89. Available from: https://doi.org/10.1016/j.vaccine.2019.10.102
28. Folegatti PM, Ewer KJ, Aley PK, Angus B, Becker S, Belij-Rammerstorfer S, et al. Safety and immunogenicity of the ChAdOx1 nCoV-19 vaccine against SARS-CoV-2: a preliminary report of a phase 1/2, single-blind, randomised controlled trial. Lancet. 2020;1–13.
29. El Zowalaty ME, Järhult JD. From SARS to COVID-19: A previously unknown SARS- related coronavirus (SARS-CoV-2) of pandemic potential infecting humans – Call for a One Health approach. One Heal [Internet]. 2020;9(February):100124. Available from: https://doi.org/10.1016/j.onehlt.2020.100124
30. University of Oxford. Oxford COVID-19 vaccine programme opens for clinical trial recruitment | University of Oxford [Internet]. 2020 [cited 2020 Mar 28]. Available from: http://www.ox.ac.uk/news/2020-03-27-oxford-covid-19-vaccine-programme-opens-clinical-trial-recruitment

23. APPENDICES

23.1. Work Plan

SERU & PPB Review	0	Month 1	Month 2	Month 3-12	Month 13	Month 14	Month 18-24
Community, Stakeholder and Public Engagement							
Study Preparation and Training							
Recruitment for Phase Ib							
Enrolment for Phase Ib							
Follow up for phase I							
Recruitment for Phase 2							
Enrolment for Phase 2							
Follow up for phase 2							
Study Close-out, final data analysis & reporting							

23.2. Roles of Investigators

INVESTIGATOR	INSTITUTION	ROLE
**George Warimwe**	KEMRI CGMRC	PI. Design, conduct, data quality control and assurance. Analysis, interpretation of results and publication.
**Philip Bejon**	KEMRI CGMRC	Design, conduct, data quality control and assurance. Analysis, interpretation of results and publication.
**Samuel Sang**	KEMRI CGMRC	Research Medical Officer. Conduct, recruitment, safety monitoring, data collection, sites coordination, interpretation of results and publication.
**Henry Karanja**	KEMRI CGMRC	Sample preparation and storage, lab assays and analysis, interpretation of results and publication.
**John Gitonga**	KEMRI CGMRC	Sample preparation and storage, lab assays and analysis, interpretation of results and publication.
**Caroline Ngetsa**	KEMRI CGMRC	Safety bloods analysis, interpretation of results and publication.

**Charles Agoti**	KEMRI CGMRC	Sample preparation, lab assays and analysis, interpretation of results and publication.
**Stanley Cheruiyot**	KEMRI CGMRC	Data management, data quality control and assurance.
**Amek Nyaguara**	KEMRI CGMRC	Coordination of surveillance activities
**Mainga Hamaluba**	KEMRI CGMRC	Head of clinical trial facility. Design, conduct, data quality control and assurance. Overall clinical lead. Analysis, interpretation of results and publication.
**Benedict Orindi**	KEMRI CGMRC	Trial statistician, data analysis, interpretation of results and publication.
**Marianne Munene**	KEMRI CGMRC	Regulatory affairs contact, interpretation of results and publication
**Neema Mturi**	KEMRI CGMRC	Head of clinical service, coordination of clinical activities
**Isabella Ochola-Oyier**	KEMRI CGMRC	Head of Bioscience department, liaison with Ministry of Health, interpretation of results and publication.
**Noni Mumba**	KEMRI CGMRC	Head of Community Engagement, coordination of engagement activities, interpretation of results and publication.
**Eunice Nduati**	KEMRI CGMRC	Sample preparation and storage, lab assays and analysis, interpretation of results and publication.
**Nadia Aliyan**	Ministry of Health	County medical lead (Kilifi), interpretation of results and publication.
**Kadondi Kasera**	Ministry of Health	Lead, COVID-19 Emergency Operations Centre,

		interpretation of results and publication.
**Adrian Hill**	University of Oxford	Design, interpretation of results and publication.
**Sarah Gilbert**	University of Oxford	Design, interpretation of results and publication.
**Teresa Lambe**	University of Oxford	Design, analysis, interpretation of results and publication.
**Andrew Pollard**	University of Oxford	Design, interpretation of results and publication.
**Alexander Douglas**	University of Oxford	Design, interpretation of results and publication.
