# Supplementary material for: Age differences in immunity to human seasonal coronaviruses and the immunogenicity of ChAdOx1 nCoV-19 (AZD1222)
Source: eBioMedicine. 2025 Jul 16;118:105847. doi: 10.1016/j.ebiom.2025.105847 (PMC12284711; doi:10.1016/j.ebiom.2025.105847)
Supplement: ISRCTN15638344 COV006_Protocol [file mmc9.pdf]

Study title: A single-blind, randomised, phase II study to determine safety and immunogenicity of the Coronavirus Disease (COVID-19) vaccine ChAdOx1 in UK healthy children and adolescents (aged 6-17)

Short title: A phase II study of a candidate COVID-19 vaccine in children (COV006)

Study reference: COV006

Protocol Version: 8.3

Date: 21 October 2021

EudraCT number: 2020-005765-13

REC Reference: 21/SC/0054

IRAS Reference: 293182

Chief Investigator: Professor Andrew Pollard
Centre for Clinical Vaccinology and Tropical Medicine
University of Oxford,
Churchill Hospital, Old Road, Headington
Oxford, OX3 7LE
Email: andrew.pollard@paediatrics.ox.ac.uk

Investigators: Professor Matthew Snape
Oxford Vaccine Group
Centre for Clinical Vaccinology and Tropical Medicine
University of Oxford,
Churchill Hospital, Old Road, Headington
Oxford, OX3 7LE
Email: matthew.snape@paediatrics.ox.ac.uk

Dr Rinn Song
Oxford Vaccine Group
Centre for Clinical Vaccinology and Tropical Medicine
University of Oxford,
Churchill Hospital, Old Road, Headington

Oxford, OX3 7LE
Email: rinn.song@paediatrics.ox.ac.uk

Professor Katie Ewer
The Jenner Institute, University of Oxford
Old Road Campus Research Building (ORCRB)
Roosevelt Drive Oxford OX3 7DQ
Email: katie.ewer@ndm.ox.ac.uk

Professor Teresa Lambe
The Jenner Institute, University of Oxford
Old Road Campus Research Building (ORCRB)
Roosevelt Drive Oxford OX3 7DQ
Email: teresa.lambe@ndm.ox.ac.uk

Professor Saul Faust
Faculty of Medicine
University of Southampton
University Hospital Southampton NHS Foundation Trust
Mailpoint 218, C Level West Wing,
Tremona Road
Southampton, SO16 6YD
s.faust@soton.ac.uk

Dr Marion Roderick
Bristol Childrens Vaccine Centre
University of Bristol
Level 6, UH Bristol Education and Research Centre
Bristol, BS2 8AE
Email: Marion.roderick@uhbw.nhs.uk

Professor Paul Heath
St George's University of London
Paediatric Infectious Diseases Research Group
Jenner Wing, Level 2
St George's Hospital, Blackshaw Road
London SW17 0QT
Email: pheath@sgul.ac.uk

Co-investigators: Dr Grace Li
Oxford Vaccine Group
Centre for Clinical Vaccinology and Tropical Medicine
University of Oxford,
Churchill Hospital, Old Road, Headington
Oxford, OX3 7LE
Email : grace.li@paediatrics.ox.ac.uk

Statistician: Dr Xinxue Liu
Oxford Vaccine Group
Centre for Clinical Vaccinology and Tropical Medicine
University of Oxford,

Churchill Hospital, Old Road, Headington
Oxford, OX3 7LE
Email: xinxue.liu@paediatrics.ox.ac.uk

Sponsor: University of Oxford

Funder: NIHR and AstraZeneca

Statistician Signature: 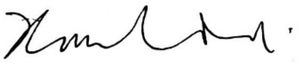

Confidentiality Statement

This document contains confidential information that must not be disclosed to anyone other than the Sponsor, the Investigator Team, HRA, host organisation, and members of the Research Ethics Committee and Regulatory Authorities unless authorised to do so.

Investigator Agreement and Notification of Conflict of Interest:

Details can be found in Appendix B

Table of Contents

1. SYNOPSIS 7
2. ABBREVIATIONS 10
3. BACKGROUND AND RATIONALE 11
3.1. Background 11
3.2. Rationale for study in this population 11
4. OBJECTIVES AND ENDPOINTS 18
5. TRIAL DESIGN 19
5.1. Study groups 22
5.2. Trial participants 22
5.3. Definition of End of Trial 22
5.4. Duration of study 22
5.5. Potential Risks for participants 22
5.6. Known Potential Benefits 23
6. RECRUITMENT AND WITHDRAWAL OF TRIAL PARTICIPANTS 24
6.1. Identification of Trial Participants 24
6.2. Informed consent 24
6.3. Inclusion and exclusion criteria 26
6.3.1. Inclusion Criteria 26
6.3.2. Exclusion Criteria 26
6.3.3. Re-vaccination exclusion criteria 27
6.3.4. Effective contraception for female participants 28
6.3.5. Withdrawal of Participants 28
6.4. Contraception 29
6.5. Pregnancy 29
7. TRIAL PROCEDURES 30
7.1. Schedule of Attendance 30
7.2. Observations 30
7.3. Study visits 31
7.3.1. Screening process 31
7.3.2. Day 0: Completion of screening, consent 31
7.3.2.1. Vaccination - D0 32
7.3.2.2. Sequence of Enrolment and Vaccination of Participants 33
7.3.3. Subsequent visits: 34

7.3.4. Participants under quarantine 34
7.3.5. 'Positive Pathway' for SARS CoV-2 infection 38
7.3.6. Medical notes review 38
7.3.7. Randomisation, blinding and code-breaking 38
8. Investigational Product 39
8.1. Description of ChAdOx1 nCoV-19 39
8.2. Supply 39
8.3. Administration 39
8.4. Rationale for selected dose 39
8.5. Control Vaccine 40
8.6. Compliance with Trial Treatment 40
8.7. Accountability of the Trial Treatment 40
8.8. Concomitant Medication 40
8.9. Provision of Treatment for Controls 41
9. Assessment of safety 41
9.1. Adverse Event (AE) 41
9.2. Adverse Reaction (AR) 41
9.3. Serious Adverse Event (SAE) 41
9.4. Serious Adverse Reaction (SAR) 42
9.5. Suspected Unexpected Serious Adverse Reaction (SUSAR) 42
9.6. Expectedness 42
9.7. Foreseeable Adverse Reactions: 42
9.8. Adverse Events of Special Interest (AESI) 42
9.9. Causality 45
9.10. Reporting Procedures for All Adverse Events 46
9.11. Reporting Procedures for Serious AEs 51
9.12. Reporting Procedures for SUSARS 51
9.13. Development Safety Update Report 51
9.14. Procedures to be followed in the event of abnormal findings 51
9.15. Interim Reviews 52
9.16. Data Safety Monitoring Board 52
9.17. Safety Group Holding Rules 52
9.17.1. Group holding rules 53
9.17.2. Individual stopping rules 54

10. STATISTICS 55
10.1. Sample size 55
10.2. Description of Statistical Methods 55
10.2.1. Safety and reactogenicity 55
10.2.2. Immunogenicity 55
8.1.3 Subgroup analyses 55
10.3. Analysis population 57
10.4. Timing for Analysis 57
10.5. The Level of Statistical Significance 57
10.6. Procedure for Accounting for Missing, Unused, and Spurious Data. 57
11. DATA MANAGEMENT 58
11.1. Data Handling 58
11.2. Record Keeping 58
11.3. Source Data and Case Report Forms (CRFs) 59
11.4. Data Protection 59
11.5. Data Quality 59
11.6. Archiving 59
12. QUALITY CONTROL AND QUALITY ASSURANCE PROCEDURES 60
12.1. Investigator procedures 60
12.2. Monitoring 60
12.3. Protocol deviation 60
12.4. Audit & inspection 60
13. SERIOUS BREACHES 61
14. ETHICS AND REGULATORY CONSIDERATIONS 62
14.1. Declaration of Helsinki 62
14.2. Guidelines for Good Clinical Practice 62
14.3. Ethical and Regulatory Approvals 62
14.4. Participant Confidentiality 62
15. FINANCING AND INSURANCE 63
15.1. Financing 63
15.2. Compensation 63
16. Publication Policy 64

1. SYNOPSIS

Trial Title	A single-blind, randomised, age-de-escalation, phase II study to determine safety and immunogenicity of the Coronavirus Disease (COVID-19) vaccine ChAdOx1 in UK healthy children and adolescents (aged 6-17)
Internal ref. no. (or short title)	A phase II study of a candidate COVID-19 vaccine in children (COV006)
Trial registration	ISRCTN 15638344
Sponsor	University of Oxford<br>Clinical Trials and Research Governance<br>Joint Research Office<br>Boundary Brook House<br>Churchill Drive<br>Headington<br>Oxford OX3 7GB<br>United Kingdom
Funder	NIHR
Clinical Phase	Phase II
Trial Design	Single-blinded, randomised, controlled, multi-centre
Trial Participants	Healthy children aged 6-17 years of age
Sample Size	261

Group	N	Age	Schedule
1	75	12-17 years	ChAdOx1 nCoV-19 5.0 x10^{10}vp (N=60) OR MenB (N=15) with homologous boost at D28
2	75		ChAdOx1 nCoV-19 5.0 x10^{10}vp (N=60) OR MenB (N=15) with homologous boost at D112
3	56	6-11 years	ChAdOx1 nCoV-19 5.0 x10^{10}vp (N=90) OR MenB (N=21) with homologous boost at D112
4	55		

Planned Trial Period	12 months post last vaccination per participant
Planned Recruitment period	Dependent on the dates of licensing of the various vaccines

	Objectives	Outcome Measures	Timepoint(s)
**Primary**	To assess the safety, tolerability and	a) occurrence of solicited local reactogenicity signs	a)-c) as stated, d)

	reactogenicity profile and tolerability of a prime boost regimen (with an interval between prime and boost of 28 or 112 days for 12-17 year olds, or interval of 112 days for 6-11 year olds) of the candidate vaccine ChAdOx1 nCoV- (5.0 x10^{10}vp /5.0 x10^{10}vp) in children aged 6-17 years	and symptoms for 7 days following vaccination;<br><br>b) occurrence of solicited systemic reactogenicity signs and symptoms for 7 days following vaccination;<br><br>c) occurrence of unsolicited adverse events (AEs) for 28 days following vaccination;<br><br>d) occurrence of SAEs and disease enhancement episodes<br><br>e) occurrence of abnormal laboratory findings (Grade 3 and above)	throughout the study, e) D2 and D7 post boost blood tests safety bloods)
**Secondary**	To assess cellular and humoral immunogenicity of ChAdOx1 nCoV-19 (5.0 x10^{10}vp /5.0 x10^{10}vp) given as homologous prime boost (at 28 and 112 days post prime in 12-17 year olds and 112 days in 6-11 year olds) in children aged 6-17 years	a) Quantify antibodies against SARS-CoV-2 spike protein<br><br>b) Virus neutralising antibody (NAb) assays against live and/or pseudotype SARS-CoV-2 virus<br><br>c) Interferon-gamma (IFN-γ) enzyme-linked immunospot (ELISpot) responses to SARS-CoV-2 spike protein<br><br>d) Cell analysis by flow cytometry assays	D0, D28, D56, D84, D112, D140, D182 and D364
Exploratory	To explore immune correlates of protection for ChAdOx1 nCoV-19	e) Functional antibody assays<br><br>f) Anti-nucleocapsid antibodies assays	D0, D28, D56, D84, D112, D140 D182 and D364
**Investigational products**			

	ChAdOx1 nCoV-19, a replication-deficient simian adenoviral vector expressing the spike (S) protein of SARS-CoV-2
Comparator	Meningococcal Group B vaccine (Bexsero®)
Formulation	ChAdOx1 nCoV-19: Liquid<br><br>Meningococcal Group B vaccine: Liquid
Route of Administration	IM
Dose per Administration	ChAdOx1 nCoV-19: $5x10^{10}$ vp<br><br>Meningococcal Group B vaccine: 0.5ml

2. ABBREVIATIONS

**AE**	Adverse event
**CCVTM**	Centre for Clinical Vaccinology and Tropical Medicine, Oxford
**CBF**	Clinical BioManufacturing Facility
**CI**	Confidence interval
**COP**	Code of Practice
**CRF**	Case Report Form or Clinical Research Facility
**CTRG**	Clinical Trials & Research Governance Office, Oxford University
**CTL**	Cytotoxic T Lymphocyte
**DSUR**	Development Safety Update Report
**ELISPOT**	Enzyme-linked immunospot
**GCP**	Good Clinical Practice
**GMO**	Genetically modified organism
**GMT**	Geometric Mean Titre
**GP**	General Practitioner
**GSK**	GlaxoSmithKline
HIV	Human immunodeficiency virus
HLA	Human leukocyte antigen
HRA	Health Research Authority
IB	Investigator Brochure
ICH	International Conference on Harmonisation
ICMJE	International Committee of Medical Journal Editors
**ID**	Intradermal
**IFNγ**	Interferon gamma
**IM**	Intramuscular
**IMP**	Investigational Medicinal Product
**IMP-D**	Investigational Medicinal Product Dossier
**IV**	Intravenous
**ME-TRAP**	Multiple epitopes and thrombospondin related adhesion protein
**MHRA**	Medicines and Healthcare Products Regulatory Agency
**MVA**	Modified vaccinia virus Ankara
**NHS**	National Health Service
**NIH**	National Institutes of Health
**NIHR**	National Institute for Health Research
PBMC	Peripheral blood mononuclear cell
PCR	Polymerase chain reaction
PHE	Public Health England
PI	Principal Investigator
**pfu**	Plaque forming unit
**qPCR**	Quantitative polymerase chain reaction
**REC**	Research Ethics Committee
**SAE**	Serious adverse event
**SC**	Subcutaneous
**SmPC**	Summary of Product characteristics
**SOP**	Standard Operating Procedure
**SUSAR**	Suspected unexpected serious adverse reaction
**VV**	Viral Vector
**WHO**	World Health Organisation

In this document, where ‘participants’ are mentioned, this may be interchanged with ‘participant or their parent/guardian’ for participants under the age of 16, unless explicitly mentioned otherwise.

3. BACKGROUND AND RATIONALE

3.1. Background

In December 2019, a cluster of patients with pneumonia of unknown cause was linked to a seafood wholesale market in Wuhan, China and were later confirmed to be infected with a novel coronavirus, known as 2019-nCoV [1]. The virus was subsequently renamed to SARS-CoV-2 because it is similar to the coronavirus responsible for severe acute respiratory syndrome (SARS-CoV), a lineage B betacoronavirus. SARS-CoV-2 shares more than 79% of its sequence with SARS-CoV, and 50% with the coronavirus responsible for Middle East respiratory syndrome (MERS-CoV), a member of the lineage C betacoronavirus [2]. COVID-19 is the infectious disease caused by SARS-CoV-2. By January 2020 there was increasing evidence of human to human transmission as the number of cases rapidly began to increase in China. Despite unprecedented containment measures adopted by the Chinese government, SARS-CoV-2 rapidly spread across the world. The WHO declared the COVID-19 outbreak a public health emergency of international concern on 30th January 2020. As of 2nd December 2020, more than 63 million cases have been reported to the World Health Organisation, with 1.5 million deaths [3].

Coronaviruses (CoVs) are spherical, enveloped, large positive-sense single-stranded RNA genomes. One-fourth of their genome is responsible for coding structural proteins, such as the spike (S) glycoprotein, envelope (E), membrane (M) and nucleocapsid (N) proteins. E, M, and N are mainly responsible for virion assembly whilst the S protein is involved in receptor binding, mediating virus entry into host cells during CoVs infection via different receptors.[4] SARS-CoV-2 belongs to the phylogenetic lineage B of the genus *Betacoronavirus* and it recognises the angiotensin-converting enzyme 2 (ACE2) as the entry receptor [5]. It is the seventh CoV known to cause human infections and the third known to cause severe disease after SARS-CoV and MERS-CoV.

The spike protein is a type I, trimeric, transmembrane glycoprotein located at the surface of the viral envelope of CoVs, which can be divided into two functional subunits: the N-terminal S1 and the C-terminal S2. S1 and S2 are responsible for cellular receptor binding via the receptor binding domain (RBD) and fusion of virus and cell membranes respectively, thereby mediating the entry of SARS-CoV-2 into target cells.[4] The roles of S in receptor binding and membrane fusion make it an ideal target for vaccine and antiviral development, as it is the main target for neutralising antibodies.

ChAdOx1 nCoV-19 vaccine consists of the replication-deficient simian adenovirus vector ChAdOx1, containing the structural surface glycoprotein (Spike protein) antigen of the SARS CoV-2 (nCoV-19), with a leading tissue plasminogen activator (tPA) signal sequence. ChAdOx1 nCoV-19 expresses a codon-optimised coding sequence for the Spike protein from genome sequence accession GenBank: MN908947. The tPA leader sequence has been shown to be beneficial in enhancing immunogenicity of another ChAdOx1 vectored CoV vaccine (ChAdOx1 MERS) [6].

3.2. Rationale for study in this population

The evidence base for the epidemiology of SARS-CoV-2 infection in children and young people is evolving rapidly. Available data suggest that the majority of COVID-19 cases in children are mild and asymptomatic, in contrast to the adult population [7]. Although children may not be prioritised for initial vaccination roll-out,

immunisation of children and young adults will be important to bring the pandemic under control. Despite widespread rollout of a vaccination programme in adults, transmission rates will still face upward pressure from incomplete vaccine uptake, vaccine failure and transmission in the classroom and educational settings (see below).

The International Severe Acute Respiratory and emerging Infection Consortium (ISARIC) Clinical Characterisation Protocol UK (CCP-UK) was deployed earlier this year and data were used to characterise features of children and young people (<19 years) admitted to hospital with confirmed SARS-CoV-2 infection in the UK. Whilst young people aged 19 years and under account for 15.4 million (23%) of the total population of 66 million people [8], they represent 651 (0.9%) of the ISARIC cohort admitted to hospital, with 6 (1%) fatalities reported [9]. This finding has been echoed by a US study using data to September 2020, showing that children represent 1.7% of hospitalisations associated with COVID-19, whilst representing 22.6% of the US population [10].

A small proportion of children are nonetheless severely affected by COVID-19. A Paediatric Inflammatory Multisystem Syndrome Temporally Associated with SARS-CoV-2 (PIMS-TS) was identified as the pandemic evolved, clinically manifesting as fever with multisystem inflammation. Some children have been critically ill with shock and multiorgan failure requiring intensive care; at least 78 cases of PIMS-TS were identified in UK Paediatric Intensive Care Units from reporting against a Royal College of Paediatrics and Child Health case definition during the first wave of SARS-CoV-2 in early 2020 [11]. Early clinical characterisation [9, 12] suggests that fever and non-specific symptoms (vomiting (45%), abdominal pain (53%) and diarrhoea (52%)) are associated with laboratory markers of inflammation (elevated CRP and ferritin), resultant shock requiring fluid resuscitation and inotropic support, and coronary artery dilatation or aneurysm in 14%. Although clinical similarities to Kawasaki disease have been noted, peripheral immunophenotyping demonstrating elevated IFNγ and activation of γδT-cell subsets (in the presence of lower CD4 and CD8 counts overall) without neutrophilia or monocytosis suggests a distinct immune aetiology [13].

Early data suggests a median age of onset of approximately 11 years (ranging from 8-14 years), and of 52 patients with PIMS-TC/MIS-C, 56% (28/50) were positive for SARS-CoV-2 antigen PCR and 44% (22/50) were SARS-CoV-2 serology positive of those tested [9]. Therefore, older children may benefit from immunisation against SARS-CoV-2 if a vaccine can be shown to protect against the occurrence of severe disease as well as PIMS-TS.

It is as yet unclear what role children and adolescents play in the transmission of SARS-CoV-2, however, as with many respiratory viruses, the school environment may be relevant to transmission. Early epidemiological meta-analyses suggest that the secondary attack rate in children is significantly lower than in adults, however, this finding is based on limited data [14]. Whilst schools and Higher Education Institutions were closed during the UK lockdown during the first wave of the pandemic in March 2020, they have since re-opened and remain so, during the second wave of infections in Autumn 2020.

ONS surveillance data show case rates per 100,000 (as of week ending 4th December 2020) trending upwards and above 100/100,000 for all year groups Y6 and older. [15] Round 7a of the REACT study shows that prevalence of swab positivity is highest in the 13-17 year age group (across all age categories, including adults) at 2.01%, with 18-24 year olds and 5-12 year olds second-highest at 1.68% and 1.27% respectively [16]. The number of outbreaks reported in school settings is also increasing. [15] It is therefore possible that children of school-going age might play a yet to be better delineated role in transmission of SARS-CoV-2, and a vaccine that could mitigate against disease transmission in this age group would be clinically valuable.

Several hypotheses have been suggested as to why COVID-19 disease appears less severe in children when compared with other respiratory viruses, and these have been explored in a recent literature review [17].

Epidemiological data gathered in the coming months of the winter 2021 might contribute to the understanding how SARS-CoV-2 infection in children and adolescents differs with respect to other common respiratory pathogens which are also responsible for hospitalisation of children in the winter months, such as RSV and influenza.

Non-clinical studies

Safety concerns around the use of full length coronavirus Spike glycoproteins and other viral antigens (nucleoprotein) as a vaccine antigen have been raised following historical and limited reports of immunopathology and antibody dependant enhancement (ADE) reported in vitro and post SARS-CoV challenge in mice, ferrets and non-human primates immunised with whole SARS-CoV inactivated or full-length S protein based vaccines, including a study using Modified Vaccinia Ankara as a vector.[18-20] To date, there has been one report of lung immunopathology following MERS-CoV challenge in mice immunised with an inactivated MERS-CoV candidate vaccine.[21] However, in preclinical studies of ChAdOx1 immunisation and MERS-CoV challenge, no ADE were observed in hDPP4 transgenic mice, dromedary camels or non-human primates. [22,23]

Existing clinical data

ChadOx1 nCov-19 has been approved for use in the UK based on an interim analysis of pooled data from four on-going randomised, blinded, controlled trials: a Phase I/II Study, COV001, in healthy adults 18 to 55 years of age in the UK; a Phase II/III Study, COV002, in adults ≥18 years of age (including the elderly) in the UK; a Phase III Study, COV003, in adults ≥18 years of age (including the elderly) in Brazil; and a Phase I/II study, COV005, in adults aged 18 to 65 years of age in South Africa [24-26]. All participants are planned to be followed for up to 12 months, for assessments of safety and efficacy against COVID-19 disease.

In the pooled analysis for efficacy (COV002 and COV003), participants ≥18 years of age received two doses of COVID-19 Vaccine AstraZeneca (N=5,807) or control (meningococcal vaccine or saline) (N=5,829). Because of logistical constraints, the interval between dose 1 and dose 2 ranged from 4 to 26 weeks.

Baseline demographics were well balanced across COVID-19 Vaccine AstraZeneca and control treatment groups. Overall, among the participants who received COVID-19 Vaccine AstraZeneca, 94.1% of participants were 18 to 64 years old (with 5.9% aged 65 or older); 60.7% of subjects were female; 82.8% were White, 4.6% were Asian, and 4.4% were Black. A total of 2,070 (35.6%) participants had at least one pre-existing comorbidity (defined as a BMI ≥30 kg/m2, cardiovascular disorder, respiratory disease or diabetes).

The most common side effects are shown below in Table 1; fatigue, headache and malaise were the most often reported by participants and many were reduced by the use of prophylactic paracetamol. Anti-spike IgG responses rose by day 28 and were boosted by a second dose, and neutralising antibody responses against SARS-CoV-2 were detected in 32 (91%) of 35 participants after a single dose when measured using a microneutralisation assay, increasing to 35 (100%) after a booster dose. Neutralising antibody responses correlated strongly with antibody levels as measured by ELISA.

<u>Table 1a: Common side effects of ChAdOx1 nCoV-19 in healthy adult subjects</u>

Side effect	Without paracetamol (%)		With paracetamol (%)	
	ChAdOx1 nCoV-19	MenACWY	ChAdOx1 nCoV-19	MenACWY
Fatigue	340 (70)	227 (48)	40 (71)	26 (46)
Headache	331 (68)	195 (41)	34 (61)	21 (37)
Muscle ache	294 (60)	15 (26)	27 48)	118 (25)
Malaise	296 (61)	6 (11)	27 (48)	83 (17)
Chills	272 (56)	5 (9)	15 (27)	46 (10)

Feeling feverish	250 (51)	5 (9)	20 (36)	38 (8)

Final determination of COVID-19 cases were made by an adjudication committee, who also assigned disease severity according to the WHO clinical progression scale. A total of 131 participants had SARS CoV 2 virologically confirmed (by nucleic acid amplification tests) COVID-19 occurring ≥15 days post dose 2 with at least one COVID-19 symptom (objective fever (defined as ≥37.8°C), cough, shortness of breath, anosmia, or ageusia) and were without evidence of previous SARS CoV 2 infection. COVID-19 Vaccine AstraZeneca significantly decreased the incidence of COVID-19 compared to control.

Vaccine efficacy from 22 days post dose 1 was 73% (95% CI 49-86%) with efficacy currently demonstrated for dose intervals from 8-12 weeks (see Table 1b):

Table 1b) SARS CoV-2 S-binding antibody response to ChAdOx1 nCoV-19

Dose interval	Baseline GMT (95% CI)	28 days after dose 1 (95% CI)	28 days after dose 2 (95% CI)
<6 weeks	(N=481) 60.51 (54.1; 67.7)	N=479) 8,734.08 (7,883.1; 9,676.9)	(N=443) 22,222.73 (20,360.50; 24,255.3)
6-8 weeks	(N=137) 58.02 (46.3; 72.6)	(N=99) 7,295.54 (5,857.4; 9,086.7)	(N=116) 24,363.10 (20,088.5, 29547.3)
9-11 weeks	(N=110) 48.79 (39.6; 60.1)	(N=87) 7,492.98 (5,885.1; 9,540.2)	(N=106) 34,754.10 (30,287.2; 39,879.8)
>12 weeks	(N=154) 52.98 (44.4; 63.2)	(N=152) 8,618.17 (7,195.4; 10,322.3)	(N=154) 63,181.59 (55,180.1; 72,343.4)

GMT: geometric mean titre, evaluated using multiplex immunoassay

COV006 will be the first time that ChAdOx1 nCoV-19 is administered to participants under the age of 18. However, ChAd vectored vaccines have previously been administered to over 400 children and adolescents aged 5-17 years of age [28] as well as children of younger ages, with acceptable safety and tolerability profiles; these children took part in clinical studies conducted by or in partnership with the University of Oxford in the UK and overseas (Table 2). ChAd vectored vaccines were given at doses ranging from 2.5 $x10^{10}$ to 1.0 x 10^{11} vp and no serious adverse reactions related to the vaccine were reported (Table 2).

Following the initial phase of recruitment of 6-11 year olds in this trial, and a review of the D7 diary card data in this population, the DSMB noted that the reactogenicity profile of the younger cohort might be higher than in the older age group and therefore safety bloods were warranted in a subset of this cohort.

The MHRA and JCVI updated their guidance regarding the use of ChAdOx1 nCoV-19 on 7th April 2021 in the under-30 age group. **As a precaution the JCVI (Joint Committee on Vaccination and Immunisation), that advises the UK government on vaccination policy, have recommended that under 30 year olds who have not yet had a first dose of the ChAdOx1 nCoV-19 vaccine, have an alternative COVID-19 vaccine. This decision was made by looking at the risk of clots following vaccination versus the benefits of receiving protection from COVID-19 disease [39].** Following this updated guidance, recruitment of the small remaining number of children in the younger age cohort was halted to be in line with the advice and a decision taken to boost all 6-11 year olds at a D84 time interval, to enable time for a further review of relevant safety data by the MHRA regarding possible adverse events, prior to further boosting all remaining participants in the trial. There is no intention to recruit further participants into this study.

A review of safety data was conducted by the MHRA to examine the risk of thrombotic thrombocytopenia following second doses of ChAdOx nCoV-19 following second doses, which was reported at 1.3 per million doses, with no reported cases in under 50 year olds. The MHRA has given permission for booster vaccinations to proceed in this trial. Therefore, the protocol (V7.0 10th May 2021) has been further amended for remaining booster doses in these groups to be given at the Day 112 timepoint, with D140 serology thereafter. Boosters given at Day 112 will be spread across at least an approximate two-week period per age group to assist in the review of diary card data should there be a safety signal and for the stopping criteria to be implemented where indicated.

Table 2: Previous clinical experience of vaccines using ChAd vectors in adolescents and children

Country	Infectious disease target for vaccine	Vector	Cohort	Schedule	Route and dose	Immunogenicity	AEFIs
Mali, Senegal [27]	Ebola	ChAd3-EBO-Z	600 children<br><br>1-5 years, 6-12 years, 13-17 years	ChAd3-EBO-Z/MenACWY-TT	1 x 10^{11} VP IM	Anti-glycoprotein Ebola virus IgG Mean concentrations were highest in 1-5 years, followed by 13-17 years and then 6-12 year old cohorts	Fever in 32% of ChAd3-EBO-Z<br>- 4-20% of 13-17 year olds<br>- 1-25% of 6-12 year olds<br>- 23-50% of 1- 5 year olds<br>-<br>Injection site pain:<br>- 31% of 13-17 year olds<br>- 41% of 6-12 year olds<br>- 55% of 1-5 year olds<br><br>• No grade 3 unsolicited adverse events<br>• 2 SAES, neither considered related to the vaccine
Burkina Faso [28]	Malaria	ChAd63 ME-TRAP	700 healthy malaria exposed children 5-17 months, Burkina Faso	ChAd63 ME-TRAP / D56 MVA ME-TRAP OR 2 doses of rabies vaccine	5 x 10^{10} VP IM (MVA 1 x 5^{10} VP)	• T cell response median 326 SFU/10^6 PBMC (CI 290-387)<br>• 72% seropositive after prime; 98% seropositive after boost	• Measured fever in 30%<br>• Reported fever in 45%<br>• Most common SAE pneumonia (12 in ChAd63 ME-TRAP arm, 10 in control arm)
Gambia [29]	Malaria	ChAd63 ME-TRAP	65 infants and neonates (16, 8 or 1 week of age)	Randomised to receive either Chad63 ME-TRAP /D56 MVA ME-TRAP and EPI OR EPI only	5 x 10^{10} VP IM (MVA 1 x 5^{10} VP)	T cell mean responses increases:<br><br>• 16wk (154-1436 SFC)<br>• 8wk (283-1759 SFC)<br>• 1 wk (254-755 SFC)<br><br>• 93% seropositivity at D63	• Fever documented in 73% in ME-TRAP+EPI versus 80% in EPI only group at 16 weeks<br>• Diarrhoea in 53% of 16/52 versus 33% of 1 wk old infants<br>• 1 case of SIDS not related to vaccine
Gambia and Burkina Faso [30,31]	Malaria	ChAd63 ME-TRAP	138 children 10 wks [n=36]	ChAd63 ME-TRAP / D56 MVA ME-TRAP	All ages except 5-17th months received both 1 x 10^{10} VP IM and 5 x	• 10 weeks old infants had significantly higher antibody responses post boost in higher dose ChAd63 ME-TRAP prime	• Approximately 10% of 2-6 year olds reported fever versus 17% of 5-12 month old and 50% of 10 week old infants

			5-12 months [n=36]<br><br>5-17 month [n=30]<br><br>2-6 year olds [n = 36]		10^{10} VP IM (followed by MVA boost)	• No significant effect of ChAd63 dose on other age groups<br>• Antibody responses in 2-6 year olds were weak, irrespective of priming or boosting dose	• 22% of 2-6 year olds reported pain at injection site

Abbreviations: EPI = Expanded Programme on Immunisation, IM = intramuscular, MVA = Modified Vaccinia virus Ankara, PBMC = peripheral blood mononuclear cell, SAE = serious adverse events, AEFIs = adverse events following immunisation

4. OBJECTIVES AND ENDPOINTS

Objectives	Outcome Measures	Time point(s) of evaluation of this outcome measure
**Primary**<br>To assess the safety, tolerability and reactogenicity profile of a prime-boost regimen (with an interval between prime and boost of 28 OR 112 days in 12-17 year olds, and interval of 112 days in 6-11 year olds) of the candidate vaccine ChAdOx1 nCoV-19 (5.0 x10^{10}vp /5.0 x10^{10}vp) in children aged 6-17 years.	a) occurrence of solicited local reactogenicity signs and symptoms for 7 days following vaccination;<br>b) occurrence of solicited systemic reactogenicity signs and symptoms for 7 days following vaccination;<br>c) occurrence of unsolicited adverse events (AEs) for 28 days following vaccination;<br>d) occurrence of SAEs and disease enhancement episodes<br>e) occurrence of abnormal laboratory findings (Grade 3 and above)	a)-c) as stated, d) throughout the study, e) D2 and D7 post-boost blood tests (safety bloods)
**Secondary**<br>To assess cellular and humoral immunogenicity of ChAdOx1 nCoV-19 (5.0 x10^{10}vp /5.0 x10^{10}vp) given as homologous prime boost (at 28 and 112 days post prime in 12-17 year olds, and 112 days post prime in children aged 6-11 years)	a) Quantify antibodies against SARS-CoV-2 spike protein (seroconversion rates)<br>b) Virus neutralising antibody (NAb) assays against live and/or pseudotype SARS-CoV-2 virus<br>c) Interferon-gamma (IFN-γ) enzyme-linked immunospot (ELISpot) responses to SARS-CoV-2 spike protein<br>d) cell analysis by flow cytometry assays	Blood samples drawn at D0, D28, D56, ,D84, D112,D140, D182 and 364
**Exploratory**<br>To explore immune correlates of protection for ChAdOx1 nCoV-19	e) Functional antibody assays<br>f) Anti-nucleocapsid antibody assays	Blood samples drawn at D0, D28, D56, , D84, D112, D140, D182 and D364

5. TRIAL DESIGN

This is a Phase II, single-blinded, -controlled, individually randomised study in healthy children and adolescents aged 6-17 years recruited in Oxford, Southampton, Bristol and London, of ChAdOx1 nCoV-19 or active control (licensed Meningococcal B vaccine) administered via an intramuscular injection into the deltoid muscle. The study will assess safety, tolerability and immunogenicity of ChAdOx1 nCoV-19.

Dose justification is discussed more extensively in section 8.4. Preliminary phase IIII trial data of ChAdOx1 nCoV-19 at 5.0 x10^{10}vp /5.0 x10^{10}vp in adults aged 18 and over suggests a favourable safety, immunogenicity and efficacy profile [25]. Data on the use of adenovirus vectors in teenage cohorts is limited, however existing studies suggest an acceptable side effect profile at 5.0 x10^{10}vp in teenagers (Table 2). Multiple studies have indicated that adverse side effects associated with adenovirus vectors increase with age de-escalation. Therefore, age de-escalation is being explored in this study. Published immunogenicity data explored D28 and D56 boosting intervals and ChadOx1 nCov-19 has been approved for use with a booster interval between 4 and 12 weeks. To bring the results of this trial in line with current policy on adult vaccination, to facilitate direct comparison with adult data should a paediatric vaccination programme be implemented, this study was initially intended to explore both 4 and 12 week booster intervals. Following the updated advice from the JCVI dated 8th April, and a subsequent review of safety data following booster dose administration in the UK adult population, a decision has been taken to boost all remaining children in the trial at a 16 week interval. This means that all children in Groups 2, 3 and 4 will be boosted at D112.There is no intention to recruit further participants into this study.

The primary focus of this study is safety and no formal hypothesis is planned for primary outcome. The sample size has been chosen based on practical constraints. A sample size of 60 participants in each age and regime provides more than 90% probability of observing at least one participant with an adverse event if the underlying incident rate is 5%. Groups 1 and 2 have a ChAdOx1 nCoV-19 group comprising up to 60 participants and each Meningococcal B group comprising up to 15 participants. Groups 3 and 4 have 55/56 participants respectively, with the ChAdOx1 nCoV-19 and MenB allocation in a 4:1 ratio. As shown in Table 3, there are 4 study groups (ChAdOx1 nCoV-19 5.0 x10^{10}vp /5.0 x10^{10}vp or licensed Meningococcal B vaccine, boosted at either 28 days or 112 days, giving a total sample size of up to 261.

Table 3: Study groups

Group	N	Age	Schedule
1	75	12-17 years	ChAdOx1 nCoV-19 5.0 x10^{10}vp (N=60) OR MenB (N=15) with homologous boost at D28
2	75		ChAdOx1 nCoV-19 5.0 x10^{10}vp (N=60) OR MenB (N=15) with homologous boost at D112
3	56	6-11 years	ChAdOx1 nCoV-19 5.0 x10^{10}vp (N=44/46)or MenB homologous boost (N=10/11) at D112
4	55		

In both age groups, participants will be randomised 4:1:4:1 to receive ChAdOx1 nCoV-19 5.0x10^{10}vp/ 5.0 x10^{10}vp (boost at D28) or MenB/MenB (boost at D28) or ChAdOx1 nCoV-19 5.0x10^{10}vp/ 5.0 x10^{10}vp (boost at D112) or MenB/MenB (boost at D112). Due to the updated guidance by the MHRA and JCVI regarding the use of ChAdOx1 nCoV-19, all the 6-11 year olds children randomised to receive a D28 boost (group 3) will be boosted at D112.

Enrolment will begin with older participants aged 12-17 years in Groups 1&2 (150 participants, 120 of whom will receive the IMP. These 150 participants will be given their prime dose first. Safety and reactogenicity data from these 150 participants will be reviewed at D7 after the prime dose and reviewed by the CI, relevant investigators and the chair of DSMB and provided for review by the MHRA before age de-escalation with the recruitment of a sentinel group of the 6-11 year cohort (5 participants from Group 3 and 5 participants from Group 4) can take place. In particular, attention will be paid to whether safety and reactogenicity support proceeding with the same dose in younger children or whether dose de-escalation may be required. This D7 safety review will also allow boosting of the 150 participants in Groups 1 and 2.

Safety data from the sentinel group of 10 6-11 year olds will again be reviewed at D7 post-prime by the CI, relevant investigators and the chair of DSMB and again provided for review by the MHRA to allow enrolling and dosing of remaining participants.

Participants or parent/guardian will complete diary cards for adverse event reporting of both local and systemic adverse effects and these will be reviewed at Day 7, prior to administering a booster dose to this cohort. Safety data will be reviewed by the CI, trial clinicians and DSMB.

Following the updated safety guidance from the JCVI on 8th April 2021, a decision was taken to halt recruitment into the study (at 261 participants) and for all remaining participants who were yet to receive a booster dose, to receive this at the 16-week interval. This would enable a further review by the MHRA/DSMB of updated safety data regarding administration of booster doses in adults, to inform a decision as to whether to continue with boosting children in this study.

Figure 2: Staggered enrolment strategy

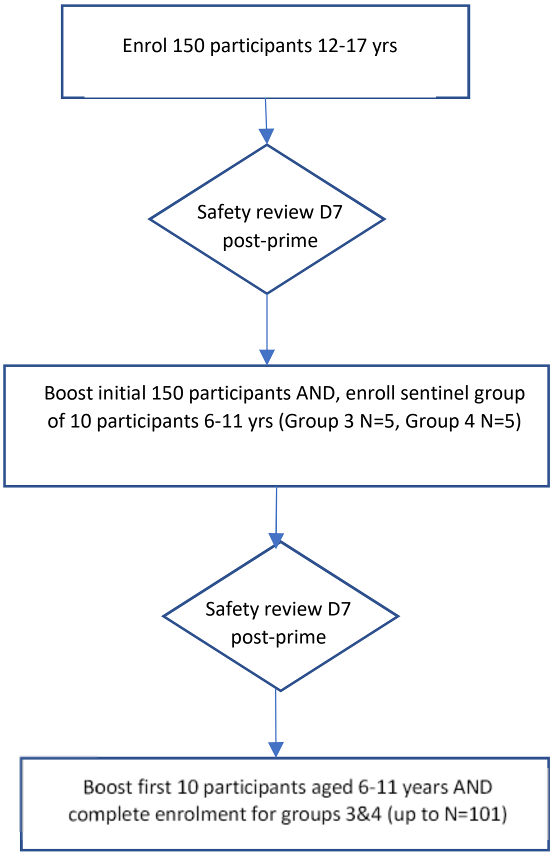

Participants will only be advised to take paracetamol for 24 hours from the time of vaccination if required for pain or other symptoms which should be recorded on their post-vaccination diary. They will not be advised to take prophylactic paracetamol.

Participants will be followed over the duration of the study to record adverse events and reported episodes of virologically confirmed symptomatic COVID-19 cases. Participants will be asked to inform the study team should they test positive in the community but no PCR testing will be done as part of this study. Participants will be provided with a phone number with which they can contact the study team clinician and the positive result together with symptoms will be entered electronically on the study database.

Mild, moderate and severe COVID-19 disease will be defined using clinical criteria, as outlined by the RCPCH [35]. These are likely to include, but are not limited to, oxygen saturation, need for oxygen therapy, respiratory rate and other vital signs, need for ventilatory support, chest imaging including CT scan, blood

test results and other relevant parameters. Should participants be admitted to hospital, participants will also be monitored for evidence of disease enhancement and evolution of PIMS-TS (via medical record review) during the study, as defined by RCPCH criteria [RCPCH, 2020].

5.1. Study groups

See Table 3.

5.2. Trial participants

Healthy children and adolescents aged 6-17 years of age will be recruited into this study. Participants will be considered enrolled immediately following administration of first vaccination.

5.3. Definition of End of Trial

The end of the trial is the date of the last assay conducted on the last sample collected.

5.4. Duration of study

The total duration of the study will be 12 months from the day of enrolment for all participants.

5.5. Potential Risks for participants

The potential risks are those associated with phlebotomy, vaccination and disease enhancement

Venepuncture

Localised bruising and discomfort can occur at the site of venepuncture. Infrequently fainting may occur. These will not be documented as AEs if they occur. Blood volumes collected will adhere to EC directive 2001/20/EC for paediatric blood volume sampling which states that 'per individual, the trial-related blood loss (including any losses in the manoeuvre) should not exceed 3 % of the total blood volume during a period of four weeks and should not exceed 1% at any single time'. Based on these calculations, we will take 10ml per sampling timepoint from participants aged 6-11 years and 15ml per sampling timepoint from participants aged 12-17 years on each visit for immunology bloods, and (an addition) 2.5ml per safety blood visit from the subgroup of participants aged 6-11 years.

Allergic reactions

Allergic reactions from mild to severe may occur in response to any constituent of a medicinal product's preparation. Anaphylaxis is extremely rare (about 1 in 1,000,000 vaccine doses) but can occur in response to any vaccine or medication.

Vaccination

Local reaction from IM vaccination

The most frequently reported adverse reactions are injection site tenderness (>60%); injection site pain, headache, fatigue (>50%); myalgia, malaise (>40%); pyrexia, chills (>30%); and arthralgia, nausea (>20%). The majority of adverse reactions are mild to moderate in severity and usually resolve within a few days of vaccination. By day 7 the incidence of subjects with at least one local or systemic reaction is 4% and 13% respectively. When compared with the first dose, adverse reactions reported after the second dose are milder and reported less frequently.

If required, analgesic and/or anti-pyretic medicinal products (e.g. paracetamol-containing products) will be advised to provide symptomatic relief from post-vaccination adverse reactions.

Systemic reactions

Short lived systemic symptoms including fatigue and headache are common but decrease with age, being reported in 86%, 77%, and 65% of those aged 18-55, 56-69 and 70 years or over respectively; most of these are classified as mild or moderate. These reactions are unusual after the second dose [25]. Mild fever (>38˚C) has been recorded in the first 48 hours for around a quarter of younger participants and but was not reported by those over 55 years of age or in any age group after the second dose [25]. Fever can be modified by the prophylactic use of paracetamol, which does not affect the immune response to this vaccine [24].

Published phase III data so far have shown only 1 serious adverse event was reported as possibly linked to the study vaccine. This was a case of transverse myelitis which occurred 14 days after dose 2 [26]. Very rare events of neuroinflammatory disorders have been reported following vaccination with COVID-19 Vaccine AstraZeneca. A causal relationship has not been established.

Control participants will receive two doses of a licensed MenB vaccine, the risks of which are described in this vaccine's SmPC.

Disease Enhancement

There were initial concerns of disease enhancement and lung immunopathology in the event of COVID-19 disease following ChAdOx1 nCoV-19 vaccination, due to an episode of disease enhancement observed in pre-clinical studies in a mouse model. However, since April 2020, ChAdOx1 nCoV-19 has been administered to over 20,000 adult participants in Phase I-III trials with as yet no evidence of disease enhancement.

Infection control procedures will be followed according to local SOP guidelines, which will be in line with local NHS Trust policy.

Following reports of blood clots with lowered platelets a review has been undertaken by the MHRA (Medicines and Healthcare products Regulatory Agency) and the EMA (European Medicines Agency). The reports were into a very rare type of blood clot in the brain, known as cerebral venous sinus thrombosis (CVST), and in some other organs together with low levels of platelets (thrombocytopenia) that might be associated with vaccination with ChAdOx1 nCoV-19. Up to and including 31 March 2021 there have been 79 UK reports of these blood clots and unfortunately 19 people died. By 31 March 2021, 20.2 million doses of the ChAdOx1 nCoV-19 vaccine had been given in the UK. This means the overall risk of these blood clots is extremely rare, approximately 4 people in a million who receive the vaccine.

5.6. Known Potential Benefits

Participants enrolled into the control groups will receive 2 doses of Meningococcal Group B vaccine, a licensed vaccine which since 2015 has been part of the routine immunisation schedule in the UK. Previous vaccination with MenB vaccine is an exclusion criterion for this study, therefore participants in this study will not have had this vaccine previously, and will gain the benefit of protection against group B meningococcus.

Recipients of the IMP, ChAdOx1 nCoV-19, may benefit from the protection offered by the vaccine. Interim phase III data from an adult study of ChAdOx1 nCoV-19 indicate that the vaccine is 60-90% effective at preventing COVID-19 when used in a homologous prime-boost regimen [26].

6. RECRUITMENT AND WITHDRAWAL OF TRIAL PARTICIPANTS

6.1. Identification of Trial Participants

Healthy children and adolescents will be recruited by use of an advertisement +/- registration form formally approved by the ethics committee(s) and distributed or posted in the places such as, but not limited to:

- In schools
- In public places, including buses and trains, with the agreement of the owner / proprietor.
- In newspapers or other literature for circulation.
- On radio via announcements.
- On a website or social media site operated by our group or with the agreement of the owner or operator (including on-line recruitment through our web-site).
- By email distribution to individuals who have already expressed an interest in taking part in any clinical trial at the Oxford Vaccine Centre and other trial sites.
- On stalls or stands at exhibitions or fairs.
- Direct mail-out using National Health Service databases: These include the National Health Applications and Infrastructure Services (NHAIS) via a NHAIS data extract or equivalent. Initial contact to potential participants will not be made by the study team. Instead invitation material (including the study reply slip and Participant Information Sheet) will be sent out on our behalf by an external company, CFH Docmail Ltd, in order to preserve the confidentiality of potential participants. CFH Docmail Ltd is accredited as having exceeded standards under the NHS Digital Data Security and Protection Toolkit (ODS ID – 8HN70).
- Oxford Vaccine Centre databases and other trial sites databases: We may contact individuals from databases of groups within the CCVTM (including the Oxford Vaccine Centre database) and other trial sites of previous trial participants who have expressed an interest in receiving information about all future studies for which they may be eligible.

6.2. Informed consent

Participants aged 16 years or over will be self-consenting as per the National Institute of Health Research guidelines. [33] However, with the participant's permission, parents/guardians will be provided with information about the study by the trial team and via information available on the trial website, and the study team will request a parent/guardian to be at the first visit. If a participant aged 16-17 years declines parental notification, this will necessitate contact with their named GP to corroborate their medical history before they are enrolled. This is to safeguard vulnerable young adults.

Consent will be taken by clinical staff (registered doctor or nurse) or non-clinical staff who have had appropriate experience and training. Where interested participants have vulnerabilities that may impair their

capacity to provide informed consent, additional input or support from the individual's parents/ guardians will be sought. If there is ongoing doubt about an individual's ability to provide informed consent, then they will not be enrolled in the study.

Children/adolescents aged under 16 of years will require full parental written consent as well as assent from the participant themselves. Participants aged 11 – 16 years will be asked to sign an assent form; in younger children a record of verbal assent will recorded. Individually each participant (and their parent/guardian) will have the opportunity to question an appropriately trained and delegated researcher before signing the consent form. Participants that turn 16 years during the course of the study will be required to sign a full informed consent form at the visit occurring after their 16th birthday.

Prior to consent, the participant (and their parent/guardian) will be fully informed of all aspects of the trial, the potential risks and their obligations. The following general principles will be emphasised:

- Participation in the study requires assent from both the child/adolescent and consent from their parent/legal guardian. In the case of adolescents aged 16 and over, we will provide information to parents and consent adolescents.
- Refusal to participate involves no penalty or loss of medical benefits
- The participant may withdraw or be withdrawn from the study at any time.
- The participant or their parent/guardian participant is free to ask questions at any time to allow him or her to understand the purpose of the study and the procedures involved
- The study involves research of an investigational vaccine
- Participants will benefit from either receiving the MenB vaccine (which enrolled participants will not have received) or potentially benefit from the ChAdOx1 nCoV-19 vaccine, with interim phase III data in adults showing 60-90% efficacy in a homologous prime-boost regimen.
- The participant's GP may be contacted to corroborate their medical history. Written or verbal information regarding the participant's medical history may be sought from the GP or other sources. This could either be via the study team accessing patient's electronic care summaries, GP and other medical records from local systems, by contacting the GP practice, or participants bringing their medical care summaries from the GP to the study clinicians. However, participants may be enrolled based on medical information obtained during screening only, at the physician's discretion.
- Blood samples taken as part of the study may be sent outside of the UK and Europe to laboratories in collaboration with the University of Oxford. These will be anonymised. Participants will be asked if they consent to indefinite storage of any leftover samples for use in other ethically approved research, this will be optional.

The aims of the study and all tests to be carried out will be explained. The participant (and their parent/guardian) will be given the opportunity to ask about details of the trial and will then have time to

consider whether or not to participate. If they do decide to participate, they or their parent/guardian and the appropriately trained and delegated researcher will sign and date the consent form. The participant (and their parent/guardian) would always have the opportunity to discuss the study with a medically qualified investigator if they wish. The participant (and their parent/guardian) will then be provided with a copy of the consent form to take away and keep, with the original being stored in the case report form (CRF). Re-consent, if required, will be taken by appropriately trained and delegated members of the team.

6.3. Inclusion and exclusion criteria

This study will be conducted in healthy children and young adults, who meet the following inclusion and exclusion criteria:

6.3.1. Inclusion Criteria

The participant must satisfy all the following criteria to be eligible for the study:

- Healthy child or adolescent aged 6-17 years (upper age limit is 17 years and 8 months so that both prime and booster are expected to take place prior to their 18th birthday)
- Able and willing (in the Investigator's opinion) to comply with all study requirements (participant's or their parents/guardians must not rely on public transport or taxis).
- Willing to allow the investigators to discuss the participant's medical history with their General Practitioner and access all medical records when relevant to study procedures.
- Parent/guardian to provide informed consent for participants under the age of 16; it will be assumed that participants aged 16 and over are able to provide consent for themselves, however parental support will be sought and investigators will reserve the right to refuse enrolment if concerns arise

6.3.2. Exclusion Criteria

The participant may not enter the study if any of the following apply:

- Child of an staff member of the Oxford Vaccine Group
- History of laboratory confirmed COVID-19 (A positive result on a validated test for SARS-CoV-2 or seropositivity for SARS-CoV-2 before enrolment)
- Chronic respiratory diseases, including mild asthma (resolved childhood asthma is allowed)
- Prior receipt of MenB vaccine
- Prior receipt of any vaccines (licensed or investigational) ≤30 days before enrolment
- Planned receipt of any vaccine other than the study intervention within 30 days before and after each study vaccination
- Prior receipt of an investigational or licensed vaccine likely to impact on interpretation of the trial data (e.g. Adenovirus vectored vaccines, any coronavirus vaccines)

- Administration of immunoglobulins and/or any blood products within the three months preceding the planned administration of the vaccine candidate
- Any confirmed or suspected immunosuppressive or immunodeficient state, including HIV infection; asplenia; recurrent severe infections and use of immunosuppressant medication within the past 6 months, except topical steroids or short-term oral steroids (course lasting <14 days)
- Any autoimmune conditions,
- History of allergic disease or reactions likely to be exacerbated by any component of the ChAdOx1 nCoV-19 or MenB vaccines
- Previous diagnosis of Kawasaki disease
- Any history of angioedema
- Any history of anaphylaxis
- Pregnancy, lactation or willingness/intention to become pregnant during the study
- Any history of cancer
- Bleeding disorder (e.g. factor deficiency, coagulopathy or platelet disorder), or prior history of significant bleeding or bruising following IM injections or venepuncture
- Any other serious chronic illness requiring hospital specialist supervision
- Congenital cardiovascular conditions
- Any other significant disease, disorder or finding which may significantly increase the risk to the participant because of participation in the study, affect the ability of the participant to participate in the study or impair interpretation of the study data.

6.3.3. Re-vaccination exclusion criteria

Participants who develop COVID-19 symptoms and have a positive PCR test after the first vaccination can only receive a booster dose after a minimum 4 weeks interval from their first PCR positive test, provided their symptoms have significantly improved. The decision to proceed with booster vaccinations in those cases will be at clinical discretion of the investigators. For participants who are asymptomatic and have a positive PCR test, a minimum of 2 weeks from first PCR positivity will be required before boosting.

The following AEs associated with any vaccine, or identified on or before the day of vaccination, constitute absolute contraindications to further administration of an IMP to the participant in question. If any of these events occur during the study, the participant will be withdrawn from the study and followed up by the clinical team or their GP until resolution or stabilisation of the event:

- Anaphylactic reaction following administration of vaccine
- Pregnancy

6.3.4. Effective contraception for female participants

All female participants aged 11 and over will be required to undergo urinary pregnancy testing at the V1 and booster visit. During the consent process, it will also be explained to all female participants aged 11 and over, that they are required to use contraception and avoid getting pregnant whilst enrolled in this study. To date, there have been more than 40 pregnancies declared by participants in COV001 and COV002, including data from South African and Brazilian sites. Green Book guidance reiterates that there are no immediate safety concerns arising from using adenovirus vectored vaccines in women of child-bearing age, however they are advised to postpone further immunisations should they fall pregnant during a course of immunisations and to postpone starting a schedule of immunisations until a pregnancy is completed [34].

6.3.5. Withdrawal of Participants

In accordance with the principles of the current revision of the Declaration of Helsinki and any other applicable regulations, a participant or their parent/guardian has the right to withdraw from the study at any time and for any reason, and is not obliged to give his or her reasons for doing so. The Investigator may withdraw the participant at any time in the interests of the participant's health and well-being. In addition, the participant may withdraw/be withdrawn by their parents/guardian for any of the following reasons:

- Administrative decision by the Investigator.
- Ineligibility (either arising during the study or retrospectively, having been overlooked at screening).
- Significant protocol deviation.
- Participant non-compliance with study requirements.
- An AE, which requires discontinuation of the study involvement or results in inability to continue to comply with study procedures.

The reason for withdrawal will be recorded in the CRF. If withdrawal is due to an AE, appropriate follow-up visits or medical care will be arranged, with the agreement of the participant (and their parents/guardian), until the AE has resolved, stabilised or a non-trial related causality has been assigned. The DSMB or DSMB chair may recommend withdrawal of participants.

Any participant who is withdrawn from the study may be replaced, if that is possible within the specified time frame. If a participant withdraws or their parent/guardian withdraws them from the study, data and blood samples collected before their withdrawal will still be used on the analysis. Storage of blood samples will continue unless the participant or their parent/guardian specifically requests otherwise. In all cases of participant's withdrawal, long-term safety data collection, including some procedures such as safety bloods, will continue as appropriate if participants have received one or more vaccine doses, unless they (and their parents/guardian) decline any further follow-up.

If a participant wishes to withdraw from the study because of the intensity of study visits or procedures and the commitment this requires, but are willing to remain in the study, they will be offered the option to discontinue some study procedures (e.g. booster dose in 2-dose groups, blood samples, vaccine diary). This

allows continued data collection on core study data for safety, immunogenicity and efficacy outcomes. This will be facilitated by having the option of visits conducted by phone remotely, and entering this data into the CRF.

6.3.6 Declining booster vaccinations

Following the updated guidance from the MHRA and JCVI on 7th April 2021 regarding the use of ChAdOx1 nCoV-19 in under 30-year olds, it may be expected that some participants or their parents wish to decline a booster vaccination. This should be recorded in the CRF and other study procedures may continue as scheduled. It will not automatically lead to withdrawal from the study unless explicitly stated by the participant or their parent.

6.4. Contraception

Female participants of childbearing potential are required to use an effective form of contraception from one month before prime until three months after boost immunisation. A participant of childbearing potential is defined as a pre-menopausal female who is capable of becoming pregnant, and for the purposes of this study all participants aged 11 and over will be asked to provide a sample for a urine pregnancy test. This will be made clear on the participant information sheets and also on the consent forms,

Acceptable forms of contraception for female volunteers include:

- Established use of oral, injected or implanted hormonal methods of contraception
- Placement of an intrauterine device (IUD) or intrauterine system (IUS)
- Total hysterectomy/Bilateral Tubal Occlusion
- Barrier methods of contraception (condom or occlusive cap with spermicide)
- Participants whom are not yet sexually active

6.5. Pregnancy

Should a participant become pregnant during the trial, no further study IMP will be administered. She will be followed up for clinical safety assessment with her ongoing consent and in addition will be followed until pregnancy outcome is determined. We would not routinely perform venepuncture in a pregnant participant unless there is clinical need.

7. TRIAL PROCEDURES

This section describes the trial procedures for evaluating study participants and follow-up after administration of study vaccine.

7.1. Schedule of Attendance

All participants will have clinic attendances and procedures as indicated in the schedule of attendances. Participants will either receive the ChAdOx1 nCoV-19 vaccine or MenB, and undergo follow-up for a total of 12 months from the first vaccination visit. Additional visits or procedures may be performed at the discretion of the investigators, e.g. further medical history and physical examination, or additional blood tests and other investigations if clinically relevant.

7.2. Observations

Temperature will be measured at the time-points indicated in the schedule of procedures. All participants will undergo an online screening questionnaire and targeted physical examination if considered necessary prior to vaccination on D0. The purpose of this examination is to assess and document the participant's baseline health status so that any later change can be determined. Temperature will be measured at pre-enrolment at visit 1 as part of baseline schedule. Further medical history, physical examination and observations may be done throughout the study based on clinical discretion.

7.3. Blood tests

Safety bloods will be taken for a subset of up to 40 participants in groups 3 and 4, as outlined in tables 5b and 5d. These will include a full blood count, urea and electrolytes, liver function tests, CRP, clotting screen and D-dimer. Reference ranges will be in accordance with local NHS guidelines. Abnormalities occurring outside this range will be graded in severity and those scoring a grade 3 or above will be classified as an AE.

Immunology; Immunogenicity will be assessed by a variety of immunological assays. This may include antibodies to SARS-CoV-Spike and non-Spike antigens by ELISA, ex vivo ELISpot assays for interferon gamma and flow cytometry assays, neutralising and other functional antibody assays and B cell analyses, virus neutralising Ab (NAb) assays against live and/or pseudotype SARS-CoV-2 virus. Other exploratory immunological assays including cytokine analysis and other antibody assays, DNA analysis of genetic polymorphisms potentially relevant to vaccine immunogenicity and gene expression studies amongst others may be performed at the discretion of the Investigators. Further exploratory immunology assays may be performed at the discretion of the Investigators on HIV and non-HIV cohorts, including, but not limited to: T cell Proliferative responses to SARS-CoV-2 antigen; T cell cross-reactivity to circulating common cold coronaviruses; Multiparameter immunophenotyping by CyTOF; BCR and TCR repertoire analysis; Serum analysis by Luminex (including inflammatory, anti-inflammatory and adaptive cytokines, chemokines, growth factors and antimicrobial proteins); HIV viral reservoir; amongst others.

Collaboration with other specialist laboratories in the UK, Europe and outside of Europe for further exploratory tests may occur. This would involve the transfer of serum or plasma, PBMC and/or other study samples to these laboratories, but these would remain anonymised. Informed consent for this will be gained from participants. This would be in line with the national guidance and policy for submitting samples for testing at reference labs. Immunological assays will be conducted according to local SOPs.

Participants or their parent/guardian will be informed that there may be leftover samples of their blood (after all testing for this study is completed), and that such samples may be stored indefinitely for possible

future research (exploratory immunology), including genotypic testing of genetic polymorphisms potentially relevant to vaccine immunogenicity. Participants or their parents/guardians will be able to decide if they will permit such future use of any leftover samples. With the participant's or parent/guardian's informed consent, any leftover cells and serum/plasma will be frozen indefinitely for future analysis of COVID-19 and other coronaviruses related diseases or vaccine-related responses. If a participant or their parent/guardian elects not to permit this, all of that participant's leftover samples will be discarded after the required period of storage to meet Good Clinical Practice (GCP) and regulatory requirements. Samples that are to be stored for future research will be transferred to the OVC Biobank (REC 16/SC/0141).

7.4. Study visits

The study visits and procedures will be undertaken by members of the clinical trials team. The procedures to be included in each visit are documented in the schedule of attendances. Each visit is assigned a time-point and a window period, within which the visit will be conducted. Visits may take place in outpatients departments or other sites which are not clinical settings such as village halls and schools as required.

7.4.1. Screening process

Initial screening

Parents/guardians or participants will be required to complete a 2-part online screening process. Firstly, they will be directed to an online questionnaire as an initial confirmation of eligibility and to screen for clear exclusion criteria. In part 2, they will be asked to indicate electronic consent for:

1. Providing details of their own or the child's s medical history
2. Permission for a telephone screening visit, if required Collection of further information from GPs where necessary, ahead of their first visit. We will aim to contact the participant's general practitioner with the permission of the participant (electronic consent) to corroborate medical history when necessary. GPs will be notified that the participant has volunteered for the study, with their parent/guardian's consent if applicable. This may require emailing confidential information to their GP and receiving medical records from their GP. This will only be done via secure NHS.net address.

Telephone screening

Where further clarification of eligibility is required, participants or their parent/guardian will be invited for a telephone screening visit. This will be recorded in a telephone screening CRF. The interval between the last screening process (whether online or telephone) and V1 will be a maximum of 90 days.

The eligibility of the participant will be reviewed electronically. Decisions to exclude the participant from enrolling in the trial will be at the discretion of the Investigator. Participants without a past medical history or drug history that requires further review may be invited directly to enrolment/vaccination visits. If eligible, a day 0 visit will be scheduled for the participant to gain informed consent, receive the vaccine and subsequent follow-up. They will also receive a link to a video with information to inform consent.

If a volunteer is not deemed eligible, their information will only be stored until the end of the trial, and this will be made clear in the PIS.

7.4.2. Day 0: Completion of screening, consent

At D0, the participant and/or parent/guardian of the participant will personally sign and date the latest approved version of the Informed Consent form. A written version and verbal explanation of the Study

Information leaflet and Informed Consent will be presented to the participant (if aged 16 and over) or their parent/guardian (if aged under 16) as well as an assent form and information leaflet for participants aged6 and over. The following information will be covered:

- The exact nature of the study
- What it will involve for the participant
- The implications and constraints of the protocol
- The known side effects and any risks involved in taking part
- The sample handling protocol – participants will be informed that anonymised samples taken during the study may be shared with study collaborators

The Participant Information Sheet will be made available to the participant for an appropriate amount of time (where possible this will be a minimum of 24 hours) prior to consent being obtained. A consent video may also be made available to participants prior to visit 1 to assist with the consent process. The following general principles will be emphasised:

- Participation in the study is entirely voluntary
- Refusal to participate involves no penalty or loss of medical benefits
- The participant may withdraw from the study at any time
- The participant is free to ask questions at any time to allow him or her to understand the purpose of the study and the procedures involved

The participant or their parent/guardian will be allowed as much time as they wish to consider the information, and the opportunity to question the Investigator, their GP or other independent parties to decide whether they will participate in the study. Written informed consent will then be obtained by means of the participant or their parent/guardian's dated signature, and dated signature of the person who presented and obtained the informed consent. The person who obtained the consent must be suitably qualified and experienced, and have been authorised to do so by the Chief/Principal Investigator and listed on the delegation log. A copy of the signed informed consent will be given to the participant. The original signed form will be retained at the research study site.

Participants will be considered enrolled into the trial at the point of vaccination. Before vaccination/trial intervention, the eligibility of the participant will be reviewed. Pulse and temperature will be observed and if necessary, a medical history and physical examination may be undertaken. Blood will be taken for safety bloods and immunology tests as per Table 5a and b. Anaesthetic cream will be offered to all participants. Vaccinations will be administered as described below.

7.4.2.1. Vaccination - D0

All vaccines will be administered intramuscularly according to specific SOPs. Observations will be taken 60 minutes after vaccination (+/- 30 minutes) and the sterile dressing removed and injection site inspected.

In all groups, participants or their parent/guardian will be given an oral thermometer, tape measure and diary card (paper or electronic), with instructions on use, along with the emergency 24-hour telephone

number on the contact card to contact the on-call study physician if needed. For participants aged under 16, it is expected that parents/guardians will communicate with the trial team by telephone and fill in the diary card. Participants aged 16 and over will be expected to fill in their own diary card and communicate with the study team directly.

Participants or their parent/guardian will be instructed on how to self-assess the severity of these AEs. There will also be space on the diary card to self-document unsolicited AEs, and whether medication was taken to relieve the symptoms. Diary cards will collect information on the timing and severity of the following solicited AEs.

In keeping with PHE guidance on vaccine associated fever during the pandemic [36], participants experiencing fever within the first 48 hours of vaccination, with no other primary symptom of COVID-19 (cough, anosmia, ageusia) will not be required to self-isolate unless there is otherwise clinical suspicion of COVID-19.

Table 4. ***Solicited AEs as collected on post vaccination diary cards***

Local solicited AEs	Systemic solicited AEs
Pain	Fever
Tenderness	Feverishness
Redness	Chills
Warmth	Joint pains
Itch	Muscle pains
Swelling	Fatigue
Induration	Headache
	Malaise
	Nausea
	Vomiting

7.4.2.2. Sequence of Enrolment and Vaccination of Participants

Please see Figure 2.

Staggered enrolment will result in 150 participants aged 12-17 being enrolled initially and given the prime dose (120 receiving the IMP and 30 receiving the control). Safety data at Day 7 will be reviewed by the CI, relevant investigators, chair of the DSMB, and the data will be provided to the MHRA for review. If there is no safety signal and no concerns from any of these reviewers, sequential enrolment will proceed as outlined in Figure 2. A full DSMB may also be consulted should safety concerns arise at this point. A second review will be conducted D7 after the first 10 participants have been enrolled in the younger age group (6-11 years).

Enrolment of the remaining participants will only proceed if the CI, and/or other designated relevant investigators and the chair of DSMB assess the data as indicating that it is safe to do so.

A further review will be conducted based on accumulated safety data once the trial is fully recruited.

7.4.3. Subsequent visits:

Follow-up visits will take place as per the schedule of attendances outlined in Tables 5a-d, with their respective windows. Safety bloods will be conducted in a subset of participants aged 6-11 years (to be determined by the sites); up to 40 participants will be allocated to have safety bloods taken at one of V3a, V3b, V4a or V4b, resulting in 6 study bloods being taken for this subgroup.. Participants will also have safety bloods taken on the day of the booster. This is to ensure accurate interpretation of any abnormal safety blood findings.

Participants will be assessed for local and systemic adverse events, interim history, physical examination, if required, review of diary cards (paper or electronic) and blood tests at these time points as detailed in the schedule of attendances. Blood will also be taken for immunology purposes and a booster vaccination given at Visit 2.

If participants experience adverse events (laboratory or clinical), which the investigator (physician), CI and/or DSMB chair determine necessary for further close observation, the participant may be admitted to an NHS hospital for observation and further medical management under the care of the Consultant on call.

7.4.4. Participants under quarantine

Given the evolving epidemiological situation both globally and in the UK, should a participant be under quarantine and unable to attend any of the scheduled visits, a telephone/video consultation will be arranged using smartphone or computer app if clinically appropriate in order to obtain core study data where possible.

Table 5a – Schedule of visits (Groups 1, D28 boost)

	Online survey to establish eligibility	V1	V2	V3	V4	V5
Study timeline	Up to D0 - 90	D0	D28	D56	D182	D364
Study windows		Within 90 days of screening	Day 28 – 42 post V1	Day 21 – 35 post V2	Day 140 - 168 post V2	Day 306 to day 366 post V2
Informed consent		X				
Medical history	X					
Urinary pregnancy test (if female > 11yrs)		X	X			
Physical examination		X				
Physical assessment (as required)		X	X	X	X	X
Vaccination		X	X			
COVID-19 immunogenicity bloods		X	X	X	X	X

Diary card review			X	X		
AE/SAE check		X	X	X	X	X

Table 5b – Schedule of visits (Groups 2, D112 boost)

	Online survey to establish eligibility	V1	V2	V3	V4	V5
Study timeline	Up to D0 - 90	D0	D84	D112	D140	D364
Study windows		Within 90 days of screening	Day 84-98 post V1	Day 112-140 post V1	Day 21-35 post V3	Day 334-394 post V1
Informed consent		X				
Medical history	X					
Urinary pregnancy test (if female > 11yrs)		X		X		
Physical examination		X		X		
Physical assessment (as required)		X	X	X	X	X
Vaccination		X		X		
COVID-19 immunogenicity bloods		X	X	X	X	X
Diary card review			X	X	X	
AE/SAE check		X	X	X	X	X

Table 5c – Schedule of visits (Groups 3, D112 boost)

	Online survey to establish eligibility	V1	V2	V3	V4	V4a* (n=10)	V4b* (n=10)	V5^{x}	V6^{x}
Study timeline	Up to D0 - 90	D0	D28	D84	D112	D2 Post Boost	D7 Post Boost	D140	D364
Study windows		Within 90 days of screening	Day 28 – 42 post V1	Day 84-98 post V1	D112-140 post V1	D2-4 after V4	D7-12 after V4	Day 21-35 post V4	Day 334-394 post V1
Informed consent		X							
Medical history	X								
Urinary pregnancy test (if female > 11yrs)		X			X				
Physical examination		X			X				
Physical assessment (as required)		X	X	X	X			X	X
Vaccination		X			X				
Safety bloods Group 3 subset only (n=20)					X – if allocated to V4a or V4b (n=20	X	X		
COVID-19 immunogenicity bloods		X	X	X	X			X	X
Diary card review			X	X	X			X	
AE/SAE check		X	X	X	X			X	X

Table 5d – Schedule of visits (Group 4 – D112 boost)

	Online survey to establish eligibility	V1	V2	V3	V3a* (n=10)	V3b* (n=10)	V4	V5
Study timeline	Up to D0 - 90	D0	84	D112	D2 Post Boost	D7 Post Boost	D140	D364
Study windows		Within 90 days of screening	Day 84-98 post V1	D112-140 post V1	D2-4 after V3	D7-12 after V3	Day 21-35 post V3	Day 334-394 post V1
Informed consent		X						
Medical history	X							
Urinary pregnancy test		X		X				

(if female > 11yrs)								
Physical examination		X		X				
Physical assessment (as required)		X	X				X	X
Vaccination		X		X				
Safety bloods Group 4 subset only (n=20)				X – if allocated to V3a or V3b (n=20)	X	X		
COVID-19 immunogenicity bloods		X	X	X			X	X
Diary card review			X				X	
AE/SAE check		X	X				X	X

In line with section 8.9, further additional visits may be required at the end of the trial, subject to approval of the IMP vaccine for use in this age group.

*a subset of participants will be allocated to have safety bloods taken at only one of timepoints V3a, V3b, V4a, V4b.

[x]participants in group 3 will be allocated to either V5 or V6. Total number of blood draws in this group will not exceed 6

7.4.5.'Positive Pathway' for SARS CoV-2 infection

The parents or guardians of participants who are under 16/participants who are 16 and over who test positive for SARS CoV-2 infection in the community during the follow-up period will be instructed to contact the study team, who will then log the case on the eCRF.

7.4.6.Medical notes review

With the participant's (if aged 16 and over) or parent/guardian's consent (and the participant's assent, if aged 11 and over), the study team will request access to medical notes or submit a data collection form for completion by attending clinical staff on any medically attended COVID-19 episodes. Any data which are relevant to disease enhancement (AESI) will be collected. These are likely to include, but not limited to, information on ICU admissions, clinical parameters such as oxygen saturation, respiratory rates and vital signs, need for oxygen therapy, need for ventilatory support, imaging and blood tests results, amongst others.

7.4.7.Randomisation, blinding and code-breaking

Participants will be randomised on a 4:1:4:1 basis to receive ChAdOx1 nCoV-19 $5.0x10^{10}$vp/ $5.0x10^{10}$vp (boost at D28) or MenB/MenB (boost at D28) or ChAdOx1 nCoV-19 $5.0x10^{10}$vp/ $5.0x10^{10}$vp (boost at D84) or MenB/MenB (boost at D84). A block randomisation with block size of 10 will be applied. The randomisation will be stratified by recruitment site and age group.

The trial staff administering the vaccine will not be blinded. Vaccines will be prepared out of sight of the participant and syringes will be covered with an opaque object/material for administration to ensure blinding.

If the clinical condition of a participant necessitates breaking the code, this will be undertaken according to a trial specific working instruction and group allocation sent to the attending physician, if unblinding is thought to be relevant and likely to change clinical management. If a participant becomes eligible for routine immunisation then they will need to be unblinded, and if they were in the control group they will be advised to receive a COVID-19 vaccine as per NHS guidelines. They will also be offered the opportunity to complete their course of MenB vaccination if they have not already received their second dose. If participants were in the ChAdOx1 arm, they will be offered the opportunity, in the trial, to complete their course of ChAOx1 vaccination, if they have not already received their second dose. All unblinded participants will be allowed to stay on in the trial for follow up visits, both for safety and immunogenicity data.

8. Investigational Product

8.1. Description of ChAdOx1 nCoV-19

ChAdOx1 nCoV-19 vaccine consists of the replication-deficient simian adenovirus vector ChAdOx1, containing the structural surface glycoprotein (Spike protein) antigens of SARS-CoV-2.

8.2. Supply

ChAdOx1 nCoV-19 has been formulated and vialed by AstraZeneca.

The vaccine is stored at 2-8C in a secure refrigerator, at the clinical site. If stored at this temperature after the first vial puncture, it can be used within up to 48 hours thereafter. All movements of the study vaccines will be documented in accordance with existing standard operating procedure (SOP). Vaccine accountability, storage, shipment and handling will be in accordance with relevant SOPs and forms. To allow for large number of participants to receive the vaccine in a short time period, additional clinic locations may be used. In this instance vaccines will be transported in accordance with local SOP's and approvals as required.

8.3. Administration

On vaccination day, the vaccine will be administered intramuscularly into the deltoid of the non-dominant arm (preferably). All participants will be observed in the unit for 1 hour (±30 minutes) after vaccination. During administration of the investigational products, Advanced Paediatric Life Support drugs and resuscitation equipment will be immediately available for the management of anaphylaxis. Vaccination will be performed and the IMPs handled according to the relevant SOPs.

8.4. Rationale for selected dose

The dose to be administered in this trial have been selected on the basis of:

1. Pre-clinical immunogenicity data
2. Safety and immunogenicity data from adenoviral vector trials in teenagers and children (Table 2]
3. Safety and immunogenicity data using ChAdOx1 nCov-19 in adults [24,25]
4. Interim efficacy data from ongoing phase III trials of ChAdOx1 nCov-19 in adults [26]

Simian adenovirus vector (ChAd63) has been safely administered in adults at doses up to 2 x 10^{11} vp, with an optimal dose of 5 x 10^{10} vp, balancing immunogenicity and reactogenicity. MERS001 was the first clinical trial of a ChAdOx1 vectored expressing the full-length Spike protein from a separate, but related betacoronavirus. ChAdOx1 MERS has been given to 31 participants to date at doses ranging from 5x10^{9} vp to 5x10^{10} vp. Despite higher reactogenicity observed at the 5x10^{10} vp, this dose was safe, with self-limiting AEs and no SARs recorded. The 5x10^{10} vp was the most immunogenic, in terms of inducing neutralising antibodies against MERS-CoV using a live virus assay [24]. Given the immunology findings and safety profile observed with a ChAdOx1 vectored vaccine against MERS-CoV, the 5x10^{10} vp dose was chosen for ChAdOx1 nCoV-1 in adults. Data on immunogenicity of ChAd-vectored homologous prime-boost regimens in children and young adults is limited. Single dosing at 1x10^{11} has been shown to be immunogenic [27], with serological responses similar to those seen in adults.

Phase I/II clinical trial data of ChAdOx1 nCoV-19 in adults demonstrate that when given as a homologous prime-boost, a dose of 5x10^{10} vp demonstrates an acceptable side effect profile (most commonly pain,

feeling feverish, chills, muscle ache, headache, malaise) which can be ameliorated with the use of prophylactic paracetamol. [24] No SAEs related to the vaccination were noted, and immunogenicity (neutralising antibody responses) were present in 100% post boost, which correlated strongly with antibody levels measured by ELISA. Interim analyses of phase III data from an ongoing adult study suggest that a LD/SD regimen (where low dose is approximately half the standard dose of $5x10^{10}$ vp) may have a greater efficacy than SD/SD regime, however confidence intervals for efficacy estimates of these two groups overlap [26].

Given that a SD/SD ($5x10^{10}$ vp homologous prime-boost) regimen of ChAdOx1 nCoV-19 now has emergency approval for use in adults in the UK (as of December 2020) and similar doses of a ChAdOx viral vector have previously been administered to children and infants, this study will explore the same dosing regimen in children. However, evidence [unpublished data] suggests that a longer interval between doses of up to 12 weeks may be associated with acceptable immunogenicity, therefore this study will explore 2 different boosting strategies, one 4 weeks post-prime and one 12 weeks post-prime. Minimising environmental contamination with genetically modified organisms (GMO)

The study will be performed in accordance with the current version of the UK Genetically Modified Organisms (Contained Use) Regulations. Approved SOPs will be followed to minimise dissemination of the recombinant vectored vaccine virus into the environment. GMO waste will be inactivated according to approved SOPs.

8.5. Control Vaccine

Participants who are allocated to the control groups will receive two injections of MenB vaccine instead of ChAdOx1 nCoV-19.

Participants will be blinded as to which intervention they are receiving. A vaccine accountability log of MenB will be maintained. There will be no additional labelling of these vaccines beyond their licensed packaging.

MenB will be stored in a locked (or access controlled) refrigerator (2°C – 8°C) at the sites, as per SmPC.

8.6. Compliance with Trial Treatment

All vaccinations will be administered by the research team and recorded in the CRF. The study medication will be at no time in the possession of the participant and compliance will not, therefore, be an issue.

8.7. Accountability of the Trial Treatment

Accountability of the IMP and control vaccine will be conducted in accordance with the relevant SOPs.

8.8. Concomitant Medication

As set out by the exclusion criteria, participants may not enter the study if they have received: any vaccine in the 30 days prior to enrolment or there is planned receipt of any other vaccine within 30 days of each vaccination, any investigational product within 30 days prior to enrolment or if receipt is planned during the study period, or if there is any use of immunosuppressant medication within 6 months prior to enrolment or if receipt is planned at any time during the study period (except topical steroids and short course of low dose steroids < 14 day). Participants will be advised to take paracetamol post-vaccination only if they develop clinical symptoms, and these should be recorded in the e-diary. They will not be advised to take prophylactic paracetamol.

8.9. Provision of Treatment for Controls

If any other vaccine shown to be efficacious at preventing COVID-19 disease is licensed for this age group during the course of this study, then participants will not be disadvantaged by their participation in the trial. Unblinding will be offered upon discussion, to facilitate participation in any national immunisation programme that is implemented. All those in the control group will be offered a full course of the IMP (ChAd0x1 nCoV-19) at the end of the study if they are not eligible for a vaccine under national immunisation guidelines, should it be approved for use in the age group. All those in the intervention arms will be offered Bexsero at the end of the study.

9. Assessment of safety

Safety will be assessed by the frequency, incidence and nature of AEs, SAEs and AESIs arising during the study. Events recorded on the diary card will be used for D7 and subsequent safety reviews by the CI, relevant investigators, DSMB and MHRA, and de-identified made available to AstraZeneca. Solicited (up to day 7) and unsolicited (up to day 28) events will be recorded on diary cards.

9.1. Adverse Event (AE)

An AE is any untoward medical occurrence in a participant, which may occur during or after administration of an IMP and does not necessarily have a causal relationship with the intervention. An AE can therefore be any unfavourable and unintended sign (including any clinically significant abnormal laboratory finding or change from baseline), symptom or disease temporally associated with the study intervention, whether or not considered related to the study intervention.

9.2. Adverse Reaction (AR)

An AR is any untoward or unintended response to an IMP. This means that a causal relationship between the IMP and an AE is at least a reasonable possibility, i.e., the relationship cannot be ruled out. All cases judged by the reporting medical Investigator as having a reasonable suspected causal relationship to an IMP (i.e. possibly, probably or definitely related to an IMP) will qualify as AR.

Adverse events that may be related to the IMP are listed in the Investigator’s Brochure for ChAdOx1 nCoV-19 and the SmPC for Meningococcal Group B vaccine (Bexsero®).

9.3. Serious Adverse Event (SAE)

An SAE is an AE that results in any of the following outcomes, whether or not considered related to the study intervention.

- Death
- Life-threatening event (i.e., the participant was, in the view of the Investigator, at immediate risk of death from the event that occurred). This does not include an AE that, if it occurred in a more severe form, might have caused death.
- Persistent or significant disability or incapacity (i.e., substantial disruption of one’s ability to carry out normal life functions).

- Hospitalisation or prolongation of existing hospitalisation, regardless of length of stay, even if it is a precautionary measure for continued observation. Hospitalisation (including inpatient or outpatient hospitalisation for an elective procedure) for a pre-existing condition that has not worsened unexpectedly does not constitute a serious AE.
- An important medical event (that may not cause death, be life threatening, or require hospitalisation) that may, based upon appropriate medical judgment, jeopardise the participant and/or require medical or surgical intervention to prevent one of the outcomes listed above. Examples of such medical events include allergic reaction requiring intensive treatment in an emergency room or clinic, blood dyscrasias, or convulsions that do not result in inpatient hospitalisation.
- Congenital malformations or birth defects – structural, functional, genetic or behavioural anomalies, including metabolic disorders that occur during fetal development and can be diagnosed prenatally, at birth or later in life

9.4. Serious Adverse Reaction (SAR)

An AE that is both serious and, in the opinion of the reporting Investigator or Sponsors, believed to be possibly, probably or definitely due to an IMP or any other study treatments, based on the information provided.

9.5. Suspected Unexpected Serious Adverse Reaction (SUSAR)

A SAR, the nature and severity of which is not consistent with the information about the medicinal product in question set out in the IB.

9.6. Expectedness

No IMP related SAEs are expected in this study. All SARs will therefore be reported as SUSARs.

9.7. Foreseeable Adverse Reactions:

The foreseeable ARs following vaccination with ChAdOx1 nCoV-19 include injection site pain, tenderness, erythema, warmth, itch, swelling, fever/feverishness, chills, joint pain, muscle pain, fatigue, headache, malaise, nausea and vomiting.

9.8. Adverse Events of Special Interest (AESI)

Severe COVID-19 cases

Severe COVID-19 disease will be defined using clinical criteria as outlined by the RCPCH [35]. Detailed clinical parameters will be collected from medical records and aligned with agreed definitions as they emerge. These are likely to include, but are not limited to, oxygen saturation, need for oxygen therapy, respiratory rate, need for ventilatory support, imaging and blood test results, amongst other clinically relevant parameters.

Acute respiratory distress, pneumonitis, acute cardiac injury, arrhythmia, septic-shock like syndrome and acute kidney injury related with COVID-19 disease will be monitored from medical records review of hospitalised participants.

PIMS-TS

The Royal College of Paediatrics and Child Health has outlined a case definition for PIMS-TS.

1. A child presenting with persistent fever, inflammation (neutrophilia, elevated CRP and lymphopaenia) and evidence of single or multi-organ dysfunction (shock, cardiac, respiratory, renal, gastrointestinal or neurological disorder) with additional features (Table 8) This may include children fulfilling full or partial criteria for Kawasaki disease.
2. Exclusion of any other microbial cause, including bacterial sepsis, staphylococcal or streptococcal shock syndromes, infections associated with myocarditis such as enterovirus (waiting for results of these investigations should not delay seeking expert advice).
3. SARS-CoV-2 PCR testing may be positive or negative

Table 8: Additional features associated with PIMS-TS [RCPCH, 2020]

**Clinical**	**Laboratory**	**Imaging and ECG**
**All:**<br>Persistent fever >38.5<br><br>**Most:**<br>Oxygen requirement<br>Hypotension<br><br>**Some:**<br>Abdominal pain<br>Confusion<br>Conjunctivitis<br>Cough<br>Diarrhoea<br>Headache<br>Lymphadenopathy<br>Mucus membrane changes<br>Neck swelling<br>Rash<br>Respiratory symptoms<br>Sore throat<br>Swollen hands and feet<br>Syncope<br>Vomiting	**All:**<br>Abnormal fibrinogen<br>Absence of potential causative organisms<br>High CRP<br>High D-Dimers<br>High ferritin<br>Hypoalbuminaemia<br>Lymphopenia<br>Neutrophilia in most – normal neutrophils in some<br><br>**Some:**<br>Acute kidney injury<br>Anaemia<br>Coagulopathy<br>High IL-10/6<br>Neutrophilia<br>Proteinuria<br>Raised CK, LDH, triglycerides<br>Raised troponin<br>Thrombocytopenia<br>Transaminitis	Echo and ECG – myocarditis, valvulitis, pericardial effusion, coronary artery dilatation<br><br>CXR – patchy symmetrical infiltrates, pleural effusion<br><br>Abdo USS – colitis, ileitis, lymphadenopathy, ascites, hepatosplenomegaly<br><br>CT chest – as for CXR – may demonstrate coronary artery abnormalities if with contrast

The Safety Platform for Emergency vaccines has outlined AESI guidelines for the development of COVID-19 vaccines. Whilst these may be more geared towards adult trial participants, they are likely to be relevant to some of the older participants in this trial.

Following updated safety guidance from the JCVI and MHRA dated 8th April 2021 [39], addition of Cerebral Venous Sinus Thrombosis, Heparin-Induced Thrombocytopenia and major thrombosis with concurrent thrombocytopenia were added to the AESIs of special interest list. Participants have been asked to be alert to the following symptoms in the first 28 days following vaccination and to seek medical attention if they occur. This warning has been inserted into patient information sheets as appropriate.

- Sudden severe headache that does not improve with usual pain killers or is getting worse
- An unusual headache which seems worse when lying down or bending over, or may be accompanied by blurred vision, nausea and vomiting, difficulty with speech, weakness, drowsiness or seizures
- New and unexplained pinprick bruising or bleeding
- Shortness of breath, chest pain, leg swelling or persistent abdominal pain

Table 9: SPEAC COVID-19 vaccine AESIs of special interest

Neurological	Isolated anosmia/ageusia*<br>Guillain-Barre Syndrome<br>Encephalitis<br>Encephalopathy<br>Brain haemorrhage<br>Seizure<br>ADEM<br>Myelitis
Haematological	Thrombosis**<br>Stroke<br>Coagulopathy<br>Pulmonary embolus<br>Other thromboembolism<br>Ischemia<br>Endothelial dysfunction<br>Thrombocytopaenia***<br>Eosinophilia****<br>Cerebral Venous Sinus Thrombosis<br>Heparin-Induced Thrombocytopenia<br>Major thrombosis with concurrent thrombocytopenia
Cardiac	Myocarditis<br>Arrhythmia<br>Heart failure

	Acute coronary syndrome<br>Ruptured aneurysm<br>Sudden cardiac death
Dermatological	Chilblain<br>Cutaneous vasculitis<br>Erythema multiforme<br>Alopecia
Gastrointestinal	Acute liver injury †† †<br>Gastrointestinal ischaemia/thrombosis<br>Appendicitis
Respiratory	ARDS††
Renal	Acute kidney injury
Other	COVID-19 disease†<br>Vasculitides<br>Anaphylaxis
*In the absence of COVID-19<br>** Excluding superficial thrombophlebitis (including line-associated)<br>*** G3 or above<br>**** This will be used as a marker skewed Th2 responses and will be routinely monitored in participants attending the COVID-19 Pathway and follow-up visits. Only G2 and above.<br>† In particular, any occurrence of suspected vaccine associated enhanced disease (VAED) as defined by most recent Brighton Collaboration Case Definition<br>†† In the absence of an infective aetiology (including COVID-19)<br>†† † As defined in Hy's Law (see **Cases falling under the Hy's Law should be reported as SAEs.** 0)	

9.9. Causality

For every AE, an interpretation of the causal relationship of the intervention to the AE in question will be made, based on the type of event; the relationship of the event to the time of vaccine administration; and the known biology of the vaccine therapy (**Error! Reference source not found.**10). Alternative causes of the AE, such as the natural history of pre-existing medical conditions, concomitant therapy, other risk factors and the temporal relationship of the event to vaccination will be considered and investigated. Causality assessment will take place during planned safety reviews, interim analyses (e.g. if a holding or stopping rule is activated) and at the final safety analysis, except for SAEs, which should be assigned by the reporting investigator, immediately, as described in SOP OVC005 Safety Reporting for CTIMPs.

Table 10: Assessment of causality

0	**No Relationship**	No temporal relationship to study product ***and***<br>Alternate aetiology (clinical state, environmental or other interventions); ***and***<br>Does not follow known pattern of response to study product
1	**Unlikely**	Unlikely temporal relationship to study product ***and***<br>Alternate aetiology likely (clinical state, environmental or other interventions) ***and***<br>Does not follow known typical or plausible pattern of response to study product.
2	**Possible**	Reasonable temporal relationship to study product; ***or***<br>Event not readily produced by clinical state, environmental or other interventions; ***or***<br>Similar pattern of response to that seen with other vaccines
3	**Probable**	Reasonable temporal relationship to study product; ***and***<br>Event not readily produced by clinical state, environment, or other interventions ***or***<br>Known pattern of response seen with other vaccines
4	**Definite**	Reasonable temporal relationship to study product; ***and***<br>Event not readily produced by clinical state, environment, or other interventions; ***and***<br>Known pattern of response seen with other vaccines

9.10. Reporting Procedures for All Adverse Events

All local and systemic AEs occurring in the 28 days following each vaccination observed by the Investigator or reported by the participant, whether or not attributed to study medication, will be recorded in electronic diaries or study database. All AEs that result in a participant's withdrawal from the study will be followed up until a satisfactory resolution occurs, or until a non-study related causality is assigned (if the participant consents to this). SAEs and Adverse Events of Special Interest (as outlined in Table 9) will be collected throughout the entire trial period.

The severity of clinical adverse events will be assessed according to scales based on FDA toxicity grading scales for healthy adolescents and children enrolled in preventive vaccine clinical trials. Severity grading criteria for temperature readings are given for adolescents. The normal ranges for a tympanic temperature in children aged 6-11 years is ***35.8-38.0 C*** (severity grading criteria not available).

Safety bloods will be assessed in accordance with normal ranges. Local sites will be responsible for following up any abnormal blood results the reference range which are of clinical concern. Severity grading scores are shown in Table 11, based on guidance from the Department of Aids (DAIDs) and OUH NHS Trust [37-38]

Test		Units	Normal range	Grade 0	Grade 1	Grade 2	Grade 3
FBC							
Haemoglobin		g/l	115-155	109-155	100-108	85-99	<85
White Blood Cells	Low	x10*9/L	5 - 13	4.51- 13.49	13.5 – 17.00	17.01 -22.00	>22.00
	High	x10*9/L			3.5-4.5	2.5-3.49	<2.5
Platelets		x10*9/L	150-450	136-400	123-135	100-124	<100
Neutrophils		x10*9/L	2.0 -8.0	1.5-8.0	1.0-1.49	0.5-0.99	<0.5
Lymphocytes		x10*9/L	1.0 -5.0	1.0-5.0	0.75-0.99	0.50-0.74	<0.5
Eosinophils		x10*9/L	0.1-1.0	0.1-1.15	1.15-2.0	2.0 – 5.0	>5.0
INR			1.0-1.1	1.1 to < 1.5 x ULN	1.5 to < 2.0 x ULN	2.0 < x ULN	
D Dimer			<500ng/ml				>0.5ng/ml
Biochemistry							
Sodium	Low	mmol/l	135 - 145	135 -146	147-148	149-150	> 150
	High	mmol/l			132-134	130-131	< 130
Potassium	Low	mmol/l	3.5 - 5.0	3.4 - 5.0	3.2-3.3	3.1	2.5-3.0
	High	mmol/l			5.1-5.2	5.3-5.4	5.5-6.5
Urea		mmol/l	2.5 – 6.0	6.0-6.8	6.8-7.9	8.0-9.5	>9.5
Creatinine		µmol/l	5-6 yrs: 25-42<br>7-8 yrs: 30-48<br>9-10 yrs: 28-57<br>11 yrs: 36-64	Up to 1.1x ULN	1.1-1.5 x ULN	>1.5 – 2 x ULN	>2-3xULN
Bilirubin		µmol/l	0 -21	Up to 1.1x ULN	1.1 – 1.6 x ULN	1.6-2.6 x ULN	>2.6xULN
ALT		IU/L	10 - 45	1.1x ULN 45-49	1.1-2.5xULN 49-112	>2.-5xULN 113-225	>5-10xULN 226-450

ALP		IU/L	6-10 yrs: 135-557<br>11-14yr (Boy) 92-549<br>11-14yr (Girl) 50-415	1-1.1 x ULN	1.1 – 2x ULN	>2.5 – 5 x ULN	>5x ULN
Albumin		g/l	32-50	28-32	28-32	25-27	<25
CRP		mg/l	<10	<10	10-3-	31-100	>100

Table 11: severity grading scales for abnormal blood results , ULN = upper limit of normal

Table 12. Severity grading criteria for local adverse events *erythema ≤2.5cm is an expected consequence of skin puncture and will therefore not be considered an adverse event

Adverse Event	Grade	Intensity
Pain at injection site	1	Pain that is easily tolerated
	2	Pain that interferes with daily activity
	3	Pain that prevents daily activity
	4	A&E visit or hospitalization
Tenderness	1	Mild discomfort to touch
	2	Discomfort with movement
	3	Significant discomfort at rest
	4	A&E visit or hospitalization
Erythema at injection site*	1	2.5 - 5 cm
	2	5.1 - 10 cm
	3	>10 cm
	4	Necrosis or exfoliative dermatitis
Induration/Swelling at injection site	1	2.5 – 5 cm and does not interfere with activity
	2	5.1 - 10 cm or interferes with activity
	3	>10 cm or prevents daily activity
	4	Necrosis

Vital Signs	Grade 1 (mild)	Grade 2 (moderate)	Grade 3 (severe)	Grade 4 Potentially Life threatening
Fever (oral)	38.0°C - 38.4°C	38.5°C – 38.9°C	39.0°C - 40°C	> 40°C

Table 13 ***Severity grading criteria for temperature (adolescents aged 13 and above).***

**GRADE 0**	None
**GRADE 1**	Mild: Transient or mild discomfort (< 48 hours); No interference with activity; No medical intervention/therapy required
**GRADE 2**	Moderate: Mild to moderate limitation in activity – some assistance may be needed; no or minimal medical intervention/therapy required

**GRADE 3**	Severe: Marked limitation in activity, some assistance usually required; medical intervention/therapy required.
**GRADE 4**	Potentially Life-threatening: requires assessment in A&E or hospitalisation

Table 14. Severity grading criteria for local and systemic AEs.

9.11. Reporting Procedures for Serious AEs

In order to comply with current regulations on SAE reporting to regulatory authorities, the event will be documented accurately and notification deadlines respected. SAEs will be reported on the SAE forms to members of the study team immediately the Investigators become aware of their occurrence, as described in SOP OVC005 Safety Reporting for CTIMPs. Copies of all reports will be forwarded for review to the Chief Investigator (as the Sponsor's representative) within 24 hours of the Investigator being aware of the suspected SAE. The DSMB will be notified of SAEs that are deemed possibly, probably or definitely related to study interventions; the chair of DSMB will be notified immediately (within 24 hours) of the Sponsor being aware of their occurrence. SAEs will not normally be reported immediately to the ethical committee(s) unless there is a clinically important increase in occurrence rate, an unexpected outcome, or a new event that is likely to affect safety of trial participants, at the discretion of the Chief Investigator and/or DSMB. In addition to the expedited reporting above, the Investigator shall include all SAEs in the annual Development Safety Update Report (DSUR) report.

All AESIs not already reported as SAEs will be outlined in the DSMB reports.

9.12. Reporting Procedures for SUSARS

All SUSARs will be reported by the sponsor delegate to the relevant Competent Authority and to the REC and other parties as applicable. For fatal and life-threatening SUSARS, this will be done no later than 7 calendar days after the Sponsor or delegate is first aware of the reaction. Any additional relevant information will be reported within 8 calendar days of the initial report. All other SUSARs will be reported within 15 calendar days.

Principal Investigators will be informed of all SUSARs for the relevant IMP for all studies with the same Sponsor, whether or not the event occurred in the current trial.

9.13. Development Safety Update Report

A Development Safety Update Report (DSUR) will be prepared annually, within 60 days of the anniversary of the first approval date from the regulatory authority for each IMP. The DSUR will be submitted by the CI to the Competent Authority, Ethics Committee, HRA (where required), Host NHS Trust and Sponsor.

9.14. Procedures to be followed in the event of abnormal findings

Abnormal clinical findings from medical history or examination will be assessed as to their clinical significance throughout the trial. Decisions to exclude the participant from enrolling in the trial or to withdraw a participant from the trial will be at the discretion of the Investigator.

9.15. Interim Reviews

The safety profile will be assessed on an on-going basis by the Investigators. The CI and relevant Investigators (as per the trial delegation log) will also review safety issues and SAEs as they arise.

An interim safety review of the first 7 days of safety data is planned after prime dose administration in all 12-17 year olds. 150 participants aged 12-17 years will have been recruited (of which 120 will be given a dose of the IMP). The data will be reviewed by the CI, relevant investigators and the chair of DSMB, as well as being provided to the MHRA, before proceeding with boosters in Groups 1 & 2, This safety review, if satisfactory, will allow age de-escalation. A sentinel group of 10 participants from the 6-11 year age groups (5 from each Group 3 & 4) will also be enrolled, given a prime dose and safety reviews by the CI, relevant investigators and the chair of DSMB and provided for review by the MHRA at D7 post prime in this sentinel group. If there is no safety signal from the D7 data in this sentinel group of 6-11 year olds, then recruitment of the remaining 111 participants in groups 3&4 will be allowed.

The DSMB will review safety data accumulated when the study is fully recruited. The DSMB will evaluate frequency of events, safety and efficacy data every 4-8 weeks and/or as required. The DSMB will make recommendations concerning the conduct, continuation or modification of the study.

9.16. Data Safety Monitoring Board

A Data Safety Monitoring Board will be appointed to:

a) periodically review and evaluate the accumulated study data for participant safety, study conduct, progress, and efficacy.

b) make recommendations concerning the continuation, modification, or termination of the trial.

There will be a minimum of three appropriately qualified committee members of whom one will be the designated chair. The DSMB will operate in accordance with the trial specific charter, which will be established before recruitment starts.

The chair of the DSMB may be contacted for advice and independent review by the Investigator or trial Sponsor in the following situations:

- Following any SAE deemed to be possibly, probably or definitively related to a study intervention.
- Any other situation where the Investigator or trial Sponsor feels independent advice or review is important.

The DSMB will review SAEs deemed possibly, probably or definitively related to study interventions. The DSMB will be notified within 24 hours of the Investigators' being aware of their occurrence. The DSMB can recommend to place the study on hold if deemed necessary following a study intervention-related SAE.

9.17. Safety Group Holding Rules

Safety holding rules have been developed considering the fact that this is the first time ChAdOx1 nCoV-19 is being administered to children.

Solicited AEs are those listed as foreseeable ARs in section 9.7 of the protocol, occurring within the first 7 days after vaccination (day of vaccination and six subsequent days). 'Unsolicited adverse events' are adverse events other than the foreseeable ARs occurring within the first 7 days, or any AEs occurring after the first 7 days after vaccination

9.17.1. Group holding rules

For safety reasons, vaccination will commence in the oldest age group, (150 participants aged 12-17 years, 120 of whom will receive the IMP). Group holding rules mentioned below will apply to all study Groups

- **Thrombosis**

  Thrombosis of any kind including Cerebral Venous Sinus Thrombosis, Heparin-Induced Thrombocytopenia, major thrombosis with concurrent thrombocytopenia, stroke, pulmonary embolus or any other thromboembolism. If a thrombotic event occurs in any age group on any further vaccinations the trial will be stopped.

- **Solicited local adverse events:**
  - If more than 25% of doses of the vaccine at a given time point (e.g. Day 0, Day 28, Day 112) in a study group are followed by the same Grade 3 solicited local adverse event beginning within 2 days after vaccination (day of vaccination and one subsequent day) and persisting at Grade 3 for >72 hrs
- **Solicited systemic adverse events:**
  - If more than 25% of doses of the vaccine at a given time point (e.g. Day 0, Day 28, Day 112) in a study group are followed by the same Grade 3 solicited systemic adverse event beginning within 2 days after vaccination (day of vaccination and one subsequent day) and persisting at Grade 3 for >72 hrs
- **Unsolicited adverse events:**
  - If more than 25% of doses of the vaccine at a given time point (e.g. Day 0, Day 28, D112) in a study group are followed by the same Grade 3 unsolicited adverse event beginning within 2 days after vaccination (day of vaccination and one subsequent day) and persisting at Grade 3 for >72 hrs
- **A serious adverse event considered possibly, probably or definitely related to vaccination occurs**

If a holding rule is activated, then further vaccinations in any group will not occur until a safety review by the DSMB, study sponsor and the chief investigator has been conducted and it is deemed appropriate to restart dosing. The Regulatory Authority will be informed and a request to restart dosing with pertinent data will be submitted as a substantial amendment. The safety review will consider:

- The relationship of the AE or SAE to the vaccine.
- The relationship of the AE or SAE to the vaccine dose, or other possible causes of the event.
- If appropriate, additional screening or laboratory testing for other participants to identify those who may develop similar symptoms and alterations to the current Participant Information Sheet (PIS) are discussed.
- New, relevant safety information from ongoing research programs on the various components of the vaccine.

The local ethics committee and vaccine manufacturers will also be notified if a holding rule is activated or released.

All vaccinated participants will be followed for safety until resolution or stabilisation (if determined to be chronic sequelae) of their AEs.

9.17.2. Individual stopping rules

In addition to the above stated group holding rules, stopping rules for individual participants will apply (i.e., indications to withdraw individuals from further vaccinations). Study participants who present with at least one of the following stopping rules will be withdrawn from further vaccination in the study:

- **Local reactions:** Injection site ulceration, abscess or necrosis
- **Systemic solicited adverse events:**
    - the participant develops a Grade 3 systemic solicited AE considered possibly, probably or definitely related within 2 days after vaccination (day of vaccination and one subsequent day) and persisting continuously at Grade 3 for > 72hrs.
- **Unsolicited adverse events:**
    - the participant has a Grade 3 adverse event, considered possibly, probably or definitely related to vaccination, persisting continuously at Grade 3 for >72hrs.
    - the participant has a SAE considered possibly, probably or definitely related to vaccination.
    - the participant has an acute allergic reaction or anaphylactic shock following the administration of vaccine investigational product.

If a participant has an acute respiratory illness (moderate or severe illness with or without fever) or a fever (oral temperature greater than 37.8°C) at the scheduled time of administration of investigational product/control, the participant will not be enrolled and will be temporarily excluded from the study. They will be permitted to return in 10 days after recovery, in line with current self-isolation guidelines.

All vaccinated participants will be followed for safety until the end of their planned participation in the study or until resolution or stabilisation (if determined to be chronic sequelae) of their AEs, providing they consent to this.

In addition to these pre-defined criteria, the study can be put on hold upon advice of the DSMB, Chief Investigator, Study Sponsor, regulatory authority, Ethical Committee(s), for any single event or combination of multiple events which, in their professional opinion, jeopardise the safety of the participants or the reliability of the data.

10. STATISTICS

10.1. Sample size

The total sample size of this trial will be 261 with 60 participants in ChAdOx1 nCoV-19 groups and 15 in each of the Meningococcal B vaccine groups for Groups 1 and 2. With a sample size of 60 participants, the probability of observing at least one participant with an adverse event will be more than 90%, if the underlying incident rate is 5%. The analysis for primary endpoints will be descriptive and there has been no formal power calculation to determine the sample size of the trial. The numbers have been therefore chosen based on practical constraints.

Following the updated safety guidance from the JCVI on 8th April 2021 [39], no more participants have been recruited. All the participant aged 6-11 years who randomised to group3 (boosted at 28 days) will be boosted at 112 days. The planned sample size of 75 has been achieved in Groups 1 and 2, and recruitment in Groups 3 and 4 stands at 55 and 56 respectively. As Groups 3 and 4 will both be boosted at D112 post prime, the two groups will be combined for all the safety and immunogenicity analysis. Therefore, the analysis of the younger age cohort will have increased power.

Description of Statistical Methods

A fully detailed statistical analysis plan will be developed and signed by the chief investigator prior to any data analysis being conducted. In brief, the analysis will incorporate the following;

10.1.1. Safety and reactogenicity

All SAEs will be presented for each group using descriptive analyses, including any evidence of disease enhancement, hospital admissions and cases of suspected PIMS-TS.

Counts and percentages of each local and systemic solicited adverse reaction from diary cards, and all unsolicited AEs, and SAEs of special interest will be presented for each group.

10.1.2. Immunogenicity

Highly skewed ELISA and neutralising antibody data will be log-transformed prior to analysis. The geometric mean concentration (GMC) and associated 95% confidence interval (CI) will be summarised by computing the anti-log of the mean of the log-transformed data.

The GMCs and CIs at day 28 post prime and boost, and the proportion of participants seroconverting (defined as a greater than a four-fold rise) from day 0 to day 28 post boost will be computed.

Spike-specific T cell responses (ELISPOT) will be presented as means and confidence intervals, or medians and interquartile ranges if non-normally distributed at all post vaccination time points.

Comparisons of continuous immunogenicity data between groups or different time points will be made using a Mann Whitney U test because the low titres below detection threshold are expected, which will cause a non-normal distribution. Comparison of seroconversion rates will be conducted using a Chi-squared test.

8.1.3 Subgroup analyses

Comparisons will include:

1. Males versus females
2. Age (6-11 versus 12-17 years)

3. SARS-CoV-2 antibody positive versus non-positive at baseline

10.2. Analysis population

All participants received at least one dose of study vaccines will be included in the analysis of safety and will be analysed according to vaccine received.

The immunogenicity analyses will include participants who are seronegative to the spike protein at baseline and received both prime and boost vaccine. Sensitivity analyses of immunogenicity data will be conducted including all vaccinated participants regardless of seropositivity status and protocol deviations.

10.3. Timing for Analysis

We will carry out an interim analysis once the immunogenicity data at D28 post boost becomes available, and a final analysis will be done at the end of the study when immunogenicity at all time points are available.

10.4. The Level of Statistical Significance

There will be no statistical significance testing for primary endpoints of safety. For secondary endpoints of immunogenicity, the level of significant will be two-sided 0.05.

10.5. Procedure for Accounting for Missing, Unused, and Spurious Data.

All available data will be included in the analysis.

© Copyright: The University of Oxford

11. DATA MANAGEMENT

Direct access will be granted to authorised representatives from the Sponsor, host institution and the regulatory authorities to permit trial-related monitoring, audits and inspections. Safety data will be made available to AstraZeneca to facilitate pharmacovigiliance monitoring of this vaccine. The study protocol, documentation, data and all other information generated will be held in strict confidence. No information concerning the study or the data will be released to any unauthorised third party, without prior written approval of the sponsor.

11.1. Data Handling

The Chief Investigator will be responsible for all data that accrues from the study.

All study data including participant diary will be recorded directly into an Electronic Data Capture (EDC) system (e.g. OpenClinica, REDCap, or similar) or onto a paper source document for later entry into EDC if direct entry is not available. This includes safety data, laboratory data and outcome data. Any additional information that needs recording but is not relevant for the CRF (such as signed consent forms etc.) will be recorded on a separate paper source document. All documents will be stored safely and securely in confidential conditions.

All adverse event data (both solicited and unsolicited) reported by the participant will be entered onto a participant's electronic diary card (eDiary) for a maximum of 28 days following administration of the IMP. The eDiary provides a full audit trial of edits and will be reviewed at each review time-points indicated in the schedule of events. Any adverse event continuing beyond the period of the diary will be copied into the eCRF and followed to resolution, if there is a causal relationship to the IMP, or to the end of the study if there is no causal relationship.

The participants will be identified by a unique trial specific number and code in any database. The name and any other identifying detail will NOT be included in any trial data electronic file.

The EDC system (CRF data) uses a relational database (MySQL/ PostgreSQL) via a secure web interface with data checks applied during data entry to ensure data quality. The database includes a complete suite of features which are compliant with GCP, EU and UK regulations and Sponsor security policies, including a full audit trail, user-based privileges, and integration with the institutional LDAP server. The MySQL and PostgreSQL database and the webserver will both be housed on secure servers maintained by the University of Oxford IT personal. The servers are in a physically secure location in Europe. Backups will be stored in accordance with the IT department schedule of daily, weekly, and monthly retained for one month, three months, and six months, respectively. The IT servers provide a stable, secure, well-maintained, and high capacity data storage environment. REDCap and OpenClinica are widely-used, powerful, reliable, well-supported systems. Access to the study's database will be restricted to the members of the study team by username and password.

11.2. Record Keeping

The Investigators will maintain appropriate medical and research records for this trial, in compliance with GCP and regulatory and institutional requirements for the protection of confidentiality of participants. The Chief Investigator, co-Investigators and clinical research nurses will have access to records. The Investigators will permit authorised representatives of the Sponsor(s), as well as ethical and regulatory agencies to

© Copyright: The University of Oxford

examine (and when required by applicable law, to copy) clinical records for the purposes of quality assurance reviews, audits and evaluation of the study safety and progress.

Following completion of the study, the trial master file, CRFs and all personal data such as contact details will be kept for a minimum of 5 years and until the youngest participant turns 21 years old , at Ardington Archives storage (Faringdon, Oxford) according to the relevant OVG/OVC SOP. Storage of this data will be reviewed every 5 years and files will be confidentially destroyed if storage is no longer required. Electronic data will be stored securely for the same period in University of Oxford electronic archives.

If participants consent to have their samples stored and used in future research, their consent form as well as the information stored within it, will be recorded, retained and stored securely as per Biobanking procedures and SOP.

11.3. Source Data and Case Report Forms (CRFs)

All protocol-required information will be collected in CRFs designed by the Investigator. All source documents will be filed in the CRF. Source documents are original documents, data, and records from which the participant's CRF data are obtained. For this study, these will include, but are not limited to, participant consent form, blood results, GP response letters, laboratory records, diaries, medical records and correspondence. In the majority of cases, CRF entries will be considered source data as the CRF is the site of the original recording (i.e. there is no other written or electronic record of data). In this study this will include, but is not limited to medical history, medication records, vital signs, physical examination records, urine assessments, blood results, adverse event data and details of vaccinations. All source data and participant CRFs will be stored securely.

Source data verification requirements will be defined in the trial risk assessment and monitoring plan.

11.4. Data Protection

The study protocol, documentation, data and all other information generated will be held in strict confidence. No information concerning the study or the data will be released to any unauthorised third party, without prior written approval of the sponsor.

11.5. Data Quality

Data collection tools will undergo appropriate validation to ensure that data are collected accurately and completely. Datasets provided for analysis will be subject to quality control processes to ensure analysed data is a true reflection of the source data.

Trial data will be managed in compliance with local data management SOPs. If additional, study specific processes are required, an approved Data Management Plan will be implemented

11.6. Archiving

Study data may be stored electronically on a secure server, and paper notes will be kept in a key-locked filing cabinet at the site. All essential documents will be retained for a minimum of 5 years after the study has finished. The need to store study data for longer in relation to licensing of the vaccine will be subject to ongoing review. For effective vaccines that may be licensed, we may store research data securely at the site at least 15 years after the end of the study, subject to adjustments in clinical trials regulations.

General archiving procedures will be conducted in compliance to SOP OVC020 Archiving.

© Copyright: The University of Oxford

12. QUALITY CONTROL AND QUALITY ASSURANCE PROCEDURES

12.1. Investigator procedures

Approved site-specific standard operating procedures (SOPs) will be used at all clinical and laboratory sites.

12.2. Monitoring

Regular monitoring will be performed according to GCP by the monitor. Following written SOPs and an approved, risk-based monitoring plan, the monitor will verify that the clinical trial is conducted and data are generated, documented and reported in compliance with the protocol, GCP and the applicable regulatory requirements. The site will provide direct access to all trial related source data/documents and reports for the purpose of monitoring and auditing by the Sponsor and inspection by local and regulatory authorities.

12.3. Protocol deviation

Any deviations from the protocol will be documented in a protocol deviation form and filed in the trial master file. Each deviation will be assessed as to its impact on participant safety and study conduct. Significant protocol deviations will be listed in the end of study report.

12.4. Audit & inspection

The QA manager conducts systems based internal audits to check that trials are being conducted according to local procedures and in compliance with study protocols, departmental SOPs, GCP and applicable regulations.

The Sponsor, trial sites, and ethical committee(s) may carry out audit to ensure compliance with the protocol, GCP and appropriate regulations.

GCP inspections may also be undertaken by the MHRA to ensure compliance with protocol and the Medicines for Human Use (Clinical Trials) Regulations 2004, as amended. The Sponsor will assist in any inspections and will support the response to the MHRA as part of the inspection procedure.

© Copyright: The University of Oxford

13. SERIOUS BREACHES

The Medicines for Human Use (Clinical Trials) Regulations contain a requirement for the notification of "serious breaches" to the MHRA within 7 days of the Sponsor becoming aware of the breach.

A serious breach is defined as “A breach of GCP or the trial protocol which is likely to effect to a significant degree

(a) the safety or physical or mental integrity of the participants of the trial; or

(b) the scientific value of the trial”.

In the event that a potential serious breach is suspected the Sponsor will be informed as soon as possible, to allow preliminary assessment of the breach and reporting to the MHRA within the required timelines.

© Copyright: The University of Oxford

14. ETHICS AND REGULATORY CONSIDERATIONS

14.1. Declaration of Helsinki

The Investigators will ensure that this study is conducted according to the principles of the current revision of the Declaration of Helsinki.

14.2. Guidelines for Good Clinical Practice

The Investigator will ensure that this trial is conducted in accordance with relevant regulations and with Good Clinical Practice.

14.3. Ethical and Regulatory Approvals

Following Sponsor approval, the protocol, informed consent form, participant information sheet and any proposed advertising material will be submitted to an appropriate Research Ethics Committee (REC), HRA (where required), regulatory authorities (MHRA in the UK), and host institution(s) for written approval. No amendments to this protocol will be made without consultation with, and agreement of, the Sponsor.

The Investigator is responsible for ensuring that changes to an approved trial, during the period for which regulatory and ethical committee(s) approval has already been given, are not initiated without regulatory and ethical committee(s)' review and approval except to eliminate apparent immediate hazards to the participant (i.e as an Urgent Safety Measure).

14.4. Participant Confidentiality

The study will comply with the UK General Data Protection Regulation (UKGDPR) and Data Protection Act 2018, which require data to be de-identified as soon as it is practical to do so. The processing of personal data of participants will be minimised by making use of a unique participant study number only on all study documents and any electronic database(s), with the exception of informed consent forms and participant ID logs. All documents will be stored securely and only accessible by study staff and authorised personnel. The study staff will safeguard the privacy of participants' personal data. A separate confidential file containing identifiable information will be stored in a secured location in accordance with the current data protection legislation. Photographs taken of vaccination sites (if required, with the participant's parent/guardian's written, informed consent) will not include the participant's face and will be identified by the date, trial code and participant's unique identifier. Once developed, photographs will be stored as confidential records, as above. This material may be shown to other professional staff, used for educational purposes, or included in a scientific publication.

© Copyright: The University of Oxford

15. FINANCING AND INSURANCE

15.1. Financing

The study is funded by the UK Government through the National Institute for Health Research and AstraZeneca15.2. Insurance

The University has a specialist insurance policy in place which would operate in the event of any participant suffering harm as a result of their involvement in the research (Newline Underwriting Management Ltd, at Lloyd's of London). NHS indemnity operates in respect of the clinical treatment which is provided.

15.2. Compensation

Participants will be offered £10 per visit for travel expenses incurred whilst participating in the study.

© Copyright: The University of Oxford

16. Publication Policy

The Investigators will be involved in reviewing drafts of the manuscripts, abstracts, press releases and any other publications arising from the study. Data from the study may also be used as part of a thesis for a PhD or MD.

© Copyright: The University of Oxford

18. References

1. Zhu, N., et al., A Novel Coronavirus from Patients with Pneumonia in China. 2019, NEJM, 2020. 382(8): 727-733.
2. Lu, R et al. Genomic characterisation and epidemiology of 2019 novel coronavirus: implications for virus origins and receptor binding. Lancet , 2020. 395(10224): 565-574.
3. World Health Organisation COVID-19 Dashboard. Accessed 5th Dec 2020: https://covid19.who.int/
4. Li, F., Structure, Function, and Evolution of Coronavirus Spike Proteins. Annual review of virology, 2016. 3(1): 237-261.
5. Zhou, P., et al., A pneumonia outbreak associated with a new coronavirus of probable bat origin. Nature, 2020.
6. Alharbi, N.K., et al. ChAdOx1 and MVA based vaccine candidates against MERS-CoV elicit neutralising antibodies and cellular immune responses in mice. Vaccine, 2017. 35(30): 3780-3788.
7. Munro APS and Faust SN. COVID-19 in children: current evidence and key questions. Curr Opin Infect Dis. 2020. 33(6):540-547
8. Overview of the UK population, Office for National Statistics. Accessed 5th December 2020. https://www.ons.gov.uk/peoplepopulationandcommunity/populationandmigration/populationestimates/articles/overviewoftheukpopulation/august2019
9. Swann OV et al. Clinical characteristics of children and young people admitted to hospital with covid-19 in United Kingdom: prospective multicentre observational cohort study. BMJ 2020. (370)m3249 doi: 10.1136/bmj.m3249.
10. Sisk B et al. National Trends of Cases of COVID-19 in Children Based on US State Health Department Data. Pediatrics, 2020.146(6)e2020027425. doi: 10.1542/peds.2020-027425.
11. Davies P, et al. Intensive care admissions of children with paediatric inflammatory multisystem syndrome temporally associated with SARS-CoV-2 (PIMS-TS) in the UK: a multicentre observational study. Lancet Child Adolesc Health, 2020. 4(9):669-677. doi: 10.1016/S2352-4642(20)30215-7.
12. Whittaker E,et al. Clinical Characteristics of 58 Children With a Pediatric Inflammatory Multisystem Syndrome Temporally Associated With SARS-CoV-2. JAMA, 2020. 324(3):259-269. doi: 10.1001/jama.2020.10369.
13. Carter MJ et al. Peripheral immunophenotypes in children with multisystem inflammatory syndrome associated with SARS-CoV-2 infection. Nat Med, 2020. 26(11):1701-1707. doi: 10.1038/s41591-020-1054-6.
14. Viner RM et al. Susceptibility to SARS-CoV-2 Infection Among Children and Adolescents Compared With Adults: A Systematic Review and Meta-analysis. JAMA Pediatr, 2020. e20457. doi: 10.1001/jamapediatrics.2020.4573.
15. Coronavirus (COVID-19) Infection Survey, UK: 4 December 2020. ONS. Accessed 5th December 2020. https://www.ons.gov.uk/peoplepopulationandcommunity/healthandsocialcare/conditionsanddiseases/bulletins/coronaviruscovid19infectionsurveypilot/4december2020
16. REACT-1 round 7 interim report. Real-time Assessment of Community Transmission Programme 2020. Accessed 5th December 2020. https://www.imperial.ac.uk/media/imperial-college/institute-of-global-health-innovation/imperial_react1_r7_interim.pdf
17. Zimmermann P, Curtis N. Why is COVID-19 less severe in children? A review of the proposed mechanisms underlying the age-related difference in severity of SARS-CoV-2 infections. Arch Dis Child, 2020. doi: 10.1136/archdischild-2020-320338.

© Copyright: The University of Oxford

18. Liu, L. et al, Anti–spike IgG causes severe acute lung injury by skewing macrophage responses during acute SARS-CoV infection. JCI insight, 2019. 4(4).

19. TsengC.T., et al., Immunization with SARS coronavirus vaccines leads to pulmonary immunopathology on challenge with the SARS virus. PLoS One, 2012. 7(4): e35421.

20. Weingartl H., et al., Immunization with modified vaccinia virus Ankara-based recombinant vaccine against severe acute respiratory syndrome is associated with enhanced hepatitis in ferrets. J Virol, 2004. 78(22): 12672-6.

21. Agrawal, A.S., et al., Immunization with inactivated Middle East Respiratory Syndrome coronavirus vaccine leads to lung immunopathology on challenge with live virus. Hum Vaccin Immunother, 2016. 12(9): 2351-6.

22. Alharbi, N.K., et al., Humoral Immunogenicity and Efficacy of a Single Dose of ChAdOx1 MERS Vaccine Candidate in Dromedary Camels. Scientific reports, 2019. 9(1): 1-11.

23. Munster, V.J., et al., Protective efficacy of a novel simian adenovirus vaccine against lethal MERS-CoV challenge in a transgenic human DPP4 mouse model. npj Vaccines, 2017. 2(1): 1-4.

24. Folegatti, P.M., et al., Safety and immunogenicity of a candidate Middle East respiratory syndrome coronavirus viral-vectored vaccine: a dose-escalation, open-label, non-randomised, uncontrolled, phase 1 trial. The Lancet Infectious Diseases, 2020.

25. Ramasamy MN et al. Safety and immunogenicity of ChAdOx1 nCoV-19 vaccine administered in a prime-boost regimen in young and old adults (COV002): a single-blind, randomised, controlled, phase 2/3 trial. Lancet, 2020. doi: 10.1016/S0140-6736(20)32466-1.

26. Merryn Voysey et al. Safety and efficacy of the ChAdOx1 nCoV-19 vaccine (AZD1222) against SARS-CoV-2: an interim analysis of four randomised controlled trials in Brazil, South Africa, and the UK. Lancet, 2020. doi: 10.1016/S0140-6736(20)32661-1. Online ahead of print.

27. Tapia MD, et al. Safety, reactogenicity, and immunogenicity of a chimpanzee adenovirus vectored Ebola vaccine in children in Africa: a randomised, observer-blind, placebo-controlled, phase 2 trial. Lancet Infect Dis, 2020. 20(6):719-730. doi: 10.1016/S1473-3099(20)30019-0.

28. Tiono AB et al. First field efficacy trial of the ChAd63 MVA ME-TRAP vectored malaria vaccine candidate in 5-17 months old infants and children. PLos One, 2018.13(12):e0208328. doi: 10.1371/journal.pone.0208328

29. Mensah VM et al. Safety and Immunogenicity of Malaria Vectored Vaccines Given with Routine Expanded Program on Immunization Vaccines in Gambian Infants and Neonates: A Randomized Controlled Trial. Front Immunol, 2017. 20(8)1551. doi: 10.3389/fimmu.2017.01551.

30. Afolabi MO et al. Safety and Immunogenicity of ChAd63 and MVA ME-TRAP in West African Children and Infants. Mol Ther, 2016. 24(8):1470-7. doi: 10.1038/mt.2016.83.

31. Bliss CM, et al. Viral Vector Malaria Vaccines Induce High-Level T Cell and Antibody Responses in West African Children and Infants. Mol Ther, 2017. 25(2):547-559. doi: 10.1016/j.ymthe.2016.11.003.

32. Pfizer and BioNTech Conclude Phase 3 study of COVID-19 Vaccine Candidate, Meeting All Primary Efficacy Endpoints. Press release. Pfizer. Accessed 5th December 2020 https://www.pfizer.com/news/press-release/press-release-detail/pfizer-and-biontech-conclude-phase-3-study-covid-19-vaccine32. Modjarrad, K., MERS-CoV vaccine candidates in development: The current landscape. Vaccine, 2016. 34(26): 2982-7.

33. NIHR guiding principles for ethical research. National Institute for Health Research. Accessed 5th December 2020. https://www.nih.gov/health-information/nih-clinical-research-trials-you/guiding-principles-ethical-research

34. Chapter 14a - COVID-19. The Green Book, 2020. Accessed online 5th December 2020. https://www.gov.uk/government/publications/covid-19-the-green-book-chapter-14a

© Copyright: The University of Oxford

35. COVID-19 - guidance for management of children admitted to hospital. Royal College of Paediatrics and Child Health, 2020. Accessed 5th December 2020. https://www.rcpch.ac.uk/resources/covid-19-guidance-management-children-admitted-hospital
36. COVID-19 vaccination programme: Information for healthcare practitioners (page 21) Accessed 12th March2021https://assets.publishing.service.gov.uk/government/uploads/system/uploads/ attachment_data/file/965177/COVID19_vaccination_programme_guidance_for_healthcare_workers 26_February_2021 v3.4.pdf
37. Division of Aids severity grading scales for Paediatric and Adult Adverse events. Accessed 29th March 2021 https://rsc.niaid.nih.gov/sites/default/files/daidsgradingcorrectedv21.pdf
38. Oxford University Hospitals NHS Trust normal ranges for paediatric blood results. Available on request. Personal communication 26th March 2021.
39. JCVI Guidance 8th April 2021. https://www.gov.uk/government/news/new-jcvi-advice-on-use-of-the-astrazeneca-covid-19-vaccine

APPENDIX A: AMENDMENT HISTORY

Amendment No.	Protocol Version No.	Date issued	Author(s) of changes	Details of Changes made
1	2.0	9th February 2021	Grace Li	Reply to Berkshire Ethics Committee, changes made to exclusion criteria to exclude children of staff member; Bexsero to be offered to all intervention arm; financial reimbursement added. Correction to name of Bristol PI.
2	3.0	1st March 2021	Grace Li, David Smith	Correction of visit date windows. Rewording the recruitment target to say 'up to' 300 participants. Repositioning of the Principal investigator signing page to appendix B. Minor typographic and word spacing corrections.
4	4.0	15th March 2021	Grace Li, David Smith	Addition of PHE guidance about the expectation of fever in the 48 hours following vaccination and minor typographic word spacing corrections.
5	5.0	25th March 2021	Grace Li	Addition of safety bloods for a subset of the participants aged 6-11 years, at the request of the DSMB. Following a review of the reactogenicity profiles from the D7 data, the DSMB have listed no formal safety concerns but have recommended the inclusion of safety bloods. This means that approximately 80 participants will undergo one additional blood test.

© Copyright: The University of Oxford

Amendment No.	Protocol Version No.	Date issued	Author(s) of changes	Details of Changes made
6	6.0	10th April 2021	Andrew Pollard, Grace Li, Rinn Song, Xinxue Liu	Changes to sample size from 300 to 261 following updated safety guidance dated 8 April 2021 from the JCVI and move to D84 booster for all Group 3 and 4 participants. Addition of clotting and D Dimer to safety bloods and amendment of severity grading scale table to incorporate clotting results. Clarification of wording around participant randomisation to reflect that stratification will occur by age group as not all sites have the same number of participants.
8	7.0	10th May 2021	Andrew Pollard, Grace Li	Change to booster interval from 84 days to 112 days for participants who are yet to receive booster vaccines.
9	8.0	28th May 2021	Andrew Pollard, Grace Li	Addition of a group holding rule for Thrombosis.<br><br>Clarification that participants unblinded due to being offered vaccine in national roll out who received a single dose of ChAdOx1 nCov-19 can receive the second dose in the trial.<br><br>Staggered booster dosing over a two week period per age group.
10	8.1	16th July 2021	Grace Li	Addition of text allowing participants to withdraw from study procedures (e.g. blood taking) but remain a participant in the trial particularly to facilitate collection of safety data. These visits may be conducted by telephone.
11	8.2	14th September 2021	Grace Li	Re-inserting deleted values in Table 11, inserting reference ranges for CRP. Following a government announcement on 13th September 2021 that teenagers aged 12-15 years of age will be offered one dose

© Copyright: The University of Oxford

**Amendment No.**	**Protocol Version No.**	**Date issued**	**Author(s) of changes**	**Details of Changes made**
				of Pfizer COVID-19 vaccine, we have updated the unblinding letter to make it clearer to participants about the next steps they need to take after being unblinded in the study.
12	8.3	21st October 2021	Grace Li	Removal of symptomatic diaries

© Copyright: The University of Oxford

APPENDIX B

Investigator Agreement and Notification of Conflict of Interest

I approve this protocol for use in the above named clinical trial and agree to abide by all provisions set forth therein.

According to the Declaration of Helsinki, 2008, I have read this protocol, and declare no/~~the following (delete as appropriate)~~ conflict of interest

Professor Sir Andrew J Pollard		05/11/2021
Chief Investigator	Signature	Date

© Copyright: The University of Oxford

Site: **Oxford University**
I have read this protocol and agree to abide by all provisions set forth therein.

According to the Declaration of Helsinki, 2008, I have read this protocol, and declare no/~~the following (delete as appropriate)~~ conflict of interest

Principal Investigator<br><br>Dr Rinn Song	Signature	Date:

Site: **St Georges University Hospital NHS Foundation Trust**
I have read this protocol and agree to abide by all provisions set forth therein.

According to the Declaration of Helsinki, 2008, I have read this protocol, and declare no/~~the following (delete as appropriate)~~ conflict of interest

Principal Investigator<br><br>Prof Paul Heath	Signature	Date:

Site: **University Hospitals Bristol and Weston NHS Foundation Trust**
I have read this protocol and agree to abide by all provisions set forth therein.

According to the Declaration of Helsinki, 2008, I have read this protocol, and declare no/~~the following (delete as appropriate)~~ conflict of interest

Principal Investigator<br><br>Dr Marion Roderick	Signature	Date:

Site: **University Hospital Southampton NHS Foundation Trust**
I have read this protocol and agree to abide by all provisions set forth therein.

According to the Declaration of Helsinki, 2008, I have read this protocol, and declare no/~~the following (delete as appropriate)~~ conflict of interest

Principal Investigator<br><br>Prof Saul Faust	Signature	Date:

© Copyright: The University of Oxford
